# Reliability of the pelvis and femur anatomical landmarks and geometry with the EOS system before and after total hip arthroplasty

**DOI:** 10.1038/s41598-022-25997-3

**Published:** 2022-12-11

**Authors:** Xavier Gasparutto, Pauline Besonhe, Peter Luca DiGiovanni, Matthieu Zingg, Sana Boudabbous, Stéphane Armand, Didier Hannouche

**Affiliations:** 1 Laboratoire de Cinésiologie Willy Taillard, Geneva University Hospitals and University of Geneva, Rue Gabrielle-Perret-Gentil 4, 1211 Geneva 14, Switzerland; 2 Division of Orthopaedic Surgery and Musculoskeletal Trauma Care, Surgery Department, Geneva University Hospitals and University of Geneva, Geneva, Switzerland; 3 Division of Radiology, Diagnosis Department, Geneva University Hospitals and University of Geneva, Geneva, Switzerland

**Keywords:** Osteoarthritis, Bone imaging, Radiography, Three-dimensional imaging, Whole body imaging

## Abstract

Bi-plane X-ray provides 3D measurements of the lower limb based on the identification of anatomical landmarks in sagittal and frontal X-rays. In clinical practice, such measurements involve multiple operators and sessions. This study aimed at evaluating the reliability of anatomical landmarks identification and geometric parameters of the pelvis and femur measured with bi-plane X-rays before and after total hip arthroplasty (THA). Twenty-eight patients undergoing primary THA were selected retrospectively. Two operators performed three reconstructions for each patient before and after THA. Intraclass correlation (ICC) and smallest detectable change (SDC) were computed for intra-operator, inter-operator, and test–retest conditions. Most anatomical landmark positions had good to excellent SDC (< 5 mm) apart from the centre of the sacral slope, greater trochanter, and anterior superior iliac spines (up to 7.1, 16.9, and 21.5 mm respectively). Geometric parameters had moderate to excellent SDC, apart from femoral and stem torsion, pelvic incidence, and APP inclination with poor SDC (9–12°). The sagittal view had significantly higher measurement errors than the frontal view. Test–retest and inter-operator conditions had no significant differences suggesting a low influence of patient posture. Osteoarthritis and the presence of implants did not seem to influence reliability and measurement error. This study could be used as a reference when assessing lower limb structure with bi-plane X-rays.

## Introduction

Bi-plane X-ray is a low dose imaging system that provides 3D parameters of the spine and lower limb based on two X-ray images that are synchronised, spatially calibrated, and orthogonal (frontal and sagittal view). The 3D parameters are computed with four steps: (1) anatomical landmarks are manually identified on the images by a trained operator (e.g. acetabulum), (2) a statistical model is fitted to those landmarks, (3) the model is refined manually, and (4) the software computes predefined parameters based on the 3D geometry of the final model. This imaging method and associated software have been mainly designed to study global sagittal spinal balance, scoliosis and lower limb measurements for total hip arthroplasty (THA) (EOS imaging, Paris, France).

In the context of THA, measurements with bi-plane X-rays typically include measurements of multiple radiological parameters such as pelvic incidence, cup orientation, or femoral offset. These measurements involve multiple operators and sessions, i.e. measurements before and after surgery, with the identifications of anatomical landmarks on the pelvis, femur, and tibia. Quantifying the reliability of measurements is critical in clinical settings or when performing research studies on large databases. Indeed, the analysis of the results will be done according to this reliability. Reliability was defined by the COSMIN taxonomy^1^ as the “degree to which the measurement is free from measurement error” and “the extent to which scores for patients who have not changed are the same for repeated measurement under several conditions”, e.g. multiple operators or multiple sessions. Since the present study will focus on measurements with bi-plane X-rays and not on the definition of a specific clinical outcome, there are two relevant properties of the reliability domain: the reliability (proportion of total variance) and the measurement error (systematic and random error). For clarity, the reliability property of the reliability domain will be referred to as reliability in the following sections.

Several studies have evaluated the reliability domain of the lower limb parameters measured with bi-plane X-rays with mainly good to excellent reliability. Measurements of pelvic parameters (pelvic incidence, sacral slope, pelvic tilt) have shown excellent intra-operator and inter-operator reliability in adolescents with idiopathic scoliosis (intraclass correlation coefficient [ICC] > 0.9)^2^ and good to excellent ICC (ICC > 0.795) for gross pelvic and acetabular morphology in one dry pelvis^3^. The measurement error of pelvis axial rotation was found to be lower than 1° on one dry pelvis^4^ and a decrease in intra-operator reliability was linked to an increase in axial rotation in six patients^5^. Regarding the acetabular cup orientation, the intra- and inter-operator reliabilities were found to be excellent (ICC > 0.9) with a confidence interval lower than 5° in ten patients with THA selected from a population of 44 patients^6^. The intra and inter-operator measurement errors of the cup inclination and anteversion were lower than 2.5° on one dry pelvis^7^. Finally, lower limb measurements (3D geometry of femur and tibia) were found to have good to excellent intra and inter-observer reliability (ICC > 0.75) in 25 patients planned for THA^8^ and lower limb length showed excellent reliability (ICC > 0.90) with one phantom limb^9^. Likewise, femoral torsion showed good to excellent inter-operator reliability (ICC > 0.86) in 30 patients with planned or completed THA^10^ and in cadaveric femur^11^ while being slightly lower for a different cohort of patients with hip osteo-arthritis^12^ (ICC of 0.711–0.769). Finally, the femoral offset showed good intra and inter-operator reliability (ICC > 0.75) in 110 patients with THA^13^.

These studies show encouraging results, but some limitations remain present in the context of THA. There is a lack of knowledge about the reliability of pelvic parameters for patients with THA. Moreover, ten patients seem like a small population to identify the intra and inter-operator reliability of cup orientation. Additionally, although phantoms and dry bones can be relevant for proofs of concept, the visibility of anatomical landmarks on such images is considerably higher than in clinical practice with THA patients. Such studies will likely provide the best-case scenario but it is expected that acquisitions performed in clinal practice will lead to lower levels of reliability and higher measurement errors. Furthermore, to the best of our knowledge, there is currently no information on test–retest reliability with patients or on the reliability of anatomical landmark identification, the only manual step performed by the operator.

Most importantly, most studies focus on reliability with ICC but a clear evaluation of measurement errors seems to be missing. To our knowledge, there is no report of smallest detectable changes (SDC) for the various measurements of positions, lengths, and angles performed with such technology.

Thus, this study aimed at evaluating the multiple properties of the reliability domain for the anatomical landmarks and geometry of the pelvis and femur measured with bi-plane X-rays in patients with THA. Tibia parameters were not included since they are not directly affected by the surgery.

## Methods

### Design of experiment

**Figure 1:**
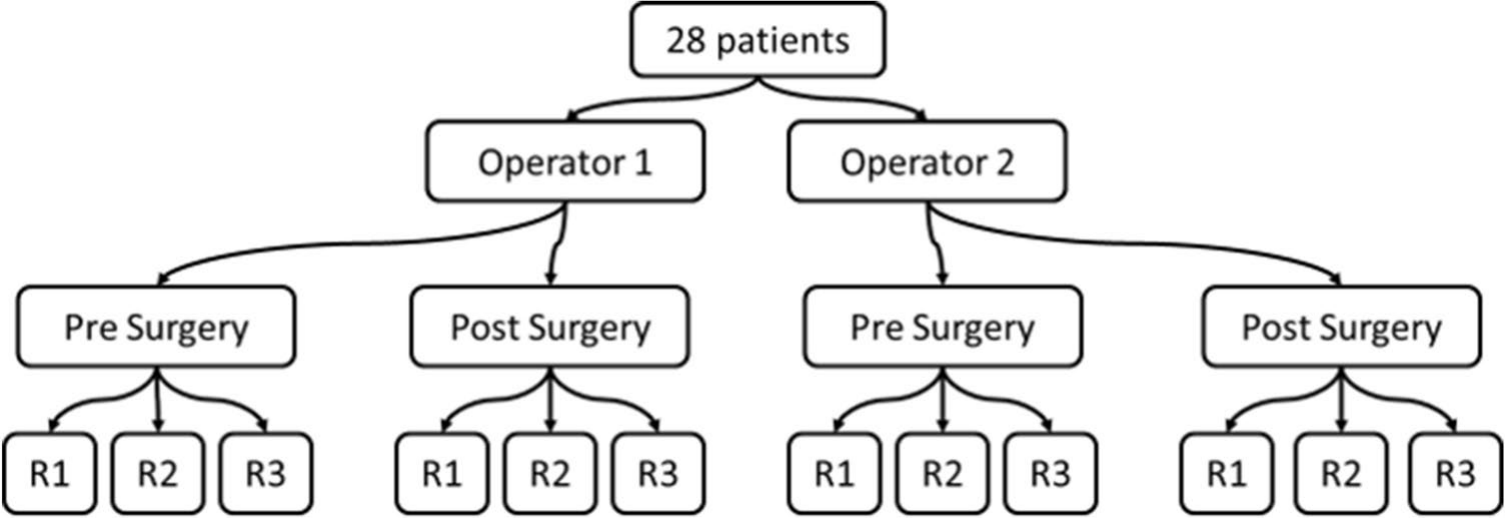
Measurement plan of the study.

Two operators performed three reconstructions at each session for each patient (Fig. 1) with a research version of the sterEOS software (EOS imaging, Paris, France). Twenty-eight patients were selected to allow the computations of ICC as low as 0.3 with a power of 80%^14^. Indeed, Guenoun et al.^8^ found ICC as low as 0.443. The reconstructions of both sessions were performed simultaneously and the delay between each reconstruction for a patient was 20 days. The operators were one trained researcher specialized in movement analysis with two years of experience in 3D reconstruction of the lower limb and one orthopaedic surgeon trained for 3D reconstructions and with extensive experience in the analysis of X-rays. This design has four degrees of freedom, i.e. sources of variance: the operators, the visits, the reconstructions, and the patients.

### Population

Twenty-eight patients were randomly selected retrospectively from a database of 439 patients with end-stage primary osteoarthritis that underwent primary unilateral THA at the Geneva University Hospitals between 2016 and 2019 and that had a bi-plane X-ray before and two months after surgery. Random selection of patients was constrained to avoid differences in distribution (Kolmogorov-Smirnoff test) and mean (Student’s t-test) for age, height, and weight, and in sex distribution (χ^2^ test) (Table 1). This study was approved by the local ethics committee (CCER Geneva, Switzerland). Patient consent was obtained and protected by the Geneva Arthroplasty Registry and all experiments were performed in accordance with relevant guidelines and regulations. Only one acquisition was done per session (before and after surgery) to avoid increasing the radiation dose exposure of the patients.

**Table 1:** Patient characteristics.

	Database	Subset	p-value	
			Student’s	KS-test
N patients	439	28	–	–
Age (years)	66.6 (12.7)	65.7 (11.7)	0.714	0.899
Height (cm)	166.8 (9.4)	168 (9.3)	0.521	0.992
Weight (kg)	76.2 (16.6)	77.8 (17.5)	0.634	0.960
Sex (% female)	56.70%	60.70%	0.828	
Side OA (% left)	46%	46.40%	1.000	

Age, height, weight are described as: mean (SD). Age, height and weight differences and distributions were tested with unpaired Student’s t-test and Kolmogorov-Smirnoff (KS) test, and sex and side of osteoarthritis (OA) distributions were tested with χ^2^.

### Anatomical landmarks

**Figure 2:**
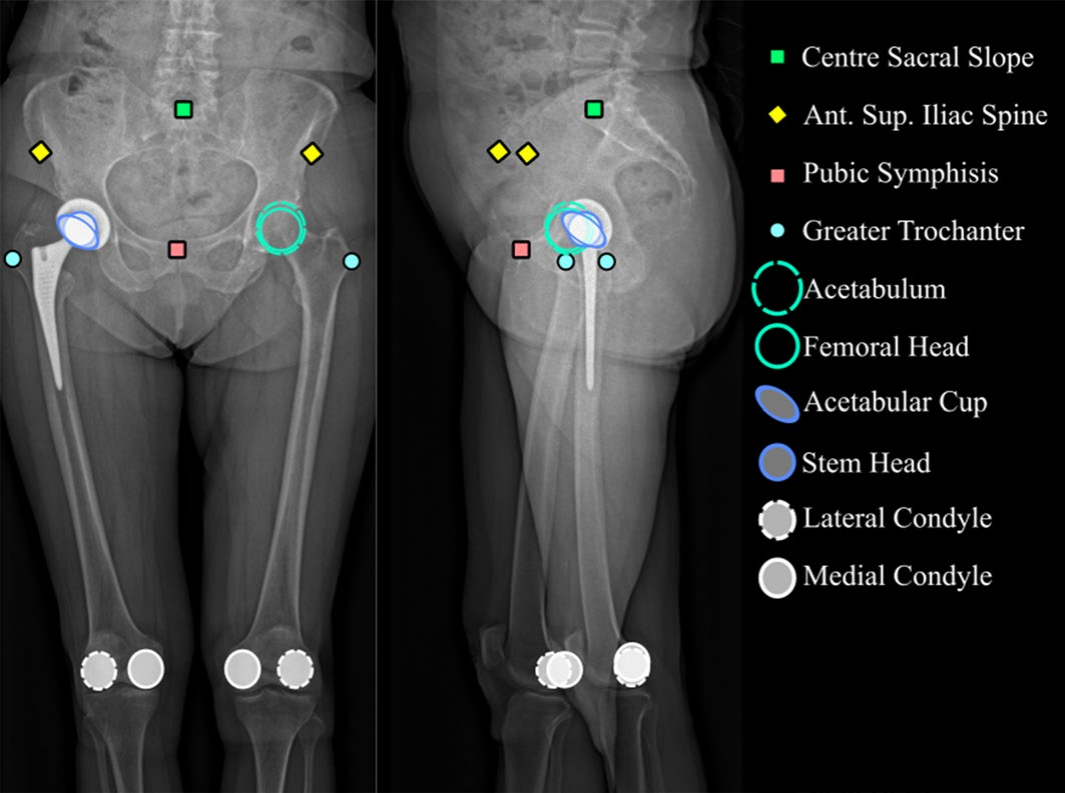
Anatomical landmarks. *Ant. Sup* anterior superior.

The considered anatomical landmarks (Fig. 2) were the points used to define the geometric model.

### Pelvis and femur geometry

**Figure 3:**
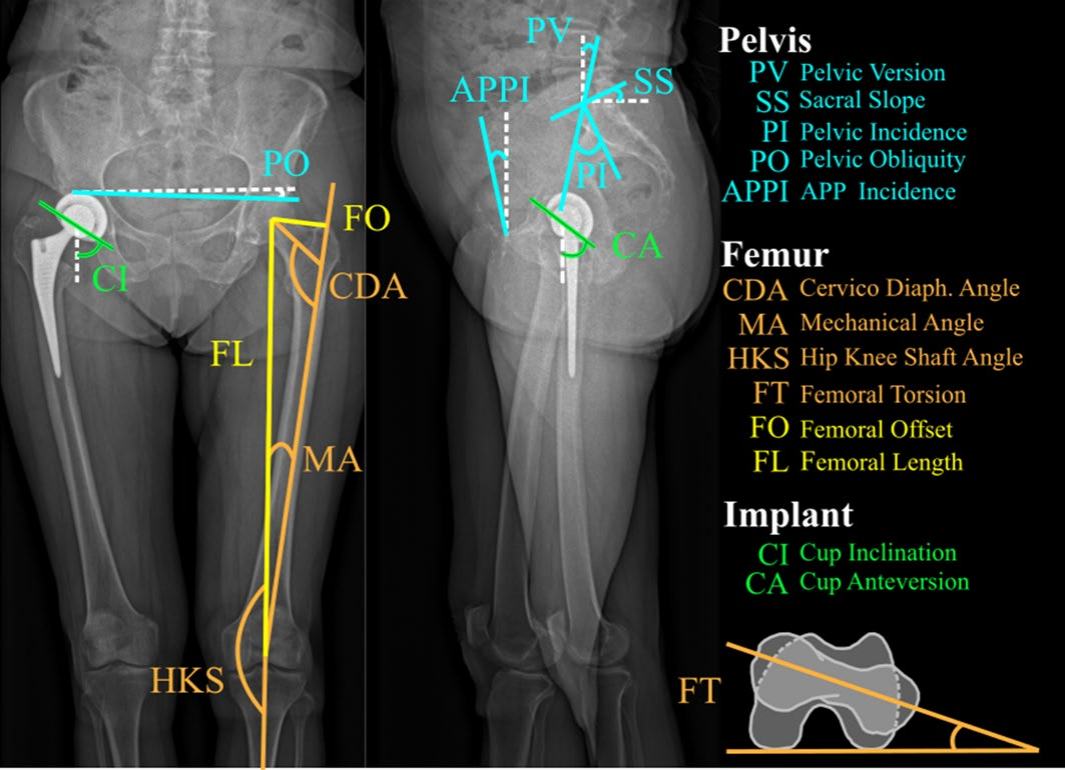
Pelvis and femur geometry parameters. *Diaph *diaphyseal.

**Figure 4:**
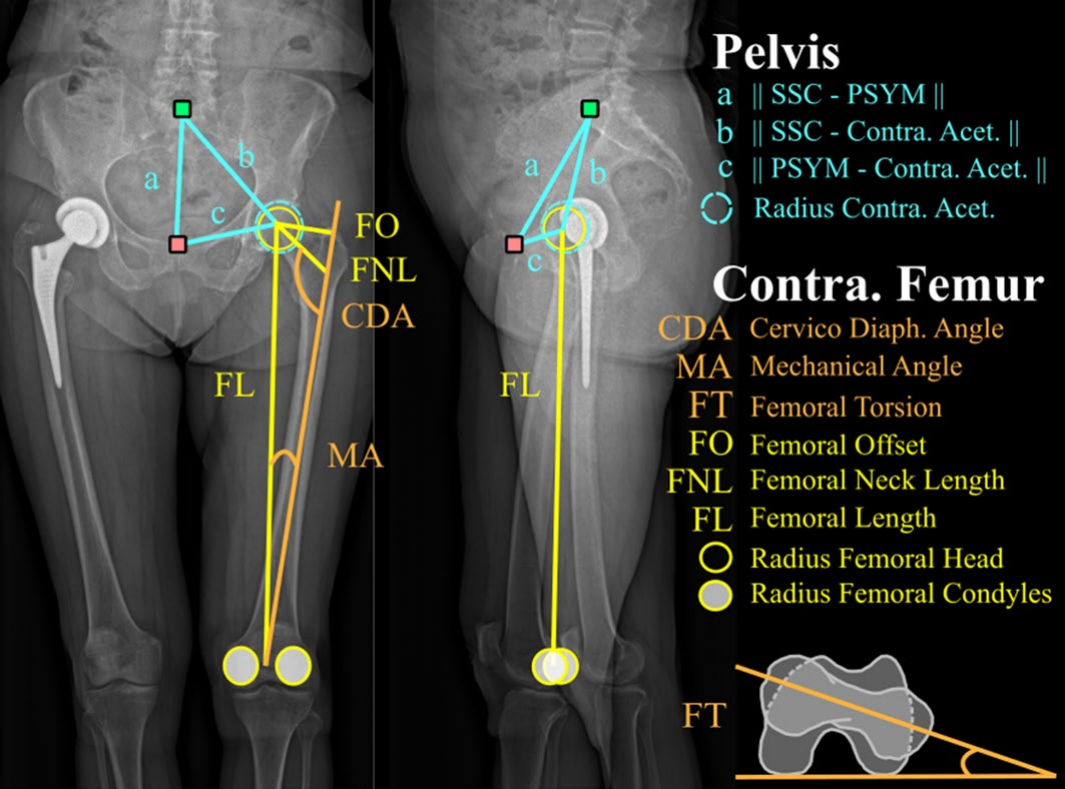
Test–retest parameters. *SSC* centre of sacral slope, *PSYM *pubic symphysis, *Contra* contralateral, *Acet* acetabulum centre, *Diaph* diaphyseal.

The selected 3D geometric parameters of the pelvis and femur were the standard output of sterEOS (Fig. 3). To assess reliability between sessions, parameters that did not depend on posture and that were not affected by surgery were selected (Fig. 4).

### Statistical analysis

According to the COSMIN checklist^1^, reliability was assessed using ICC and measurement error with SDC. The ICC is defined as the correlations between two measurements that have exactly the same components except for the ‘class’ component^15^. Variance of class components was computed from a single measure, two-way mixed effects model^16,17^ with the lme4 package^18^ of R^19^. The total variance was computed as the sum of the variance of class components:

$${\sigma }_{Total}^{2}={\sigma }_{p}^{2}+{\sigma }_{po}^{2}+{\sigma }_{pv}^{2}+{\sigma }_{pm}^{2}+{\sigma }_{residual}^{2}$$

where each term refers to a variance component: *p* = patient, *o* = operator, *v* = visit, *m* = repeated measure.

Intra-rater, inter-rater and test–retest ICCs were computed using the following formula^20^:

Intra-rater reliability:

$${ICC}_{Intra}=\frac{{\sigma }_{Total}^{2}-({\sigma }_{pm}^{2}+{\sigma }_{residual}^{2})}{{\sigma }_{Total}^{2}}$$

Inter-rater reliability:

$${ICC}_{inter}=\frac{{\sigma }_{Total}^{2}-({\sigma }_{po}^{2}+{\sigma }_{residual}^{2})}{{\sigma }_{Total}^{2}}$$

Test–retest reliability:

$${ICC}_{test-retest}=\frac{{\sigma }_{Total}^{2}-({\sigma }_{pv}^{2}+{\sigma }_{residual}^{2})}{{\sigma }_{Total}^{2}}$$

The standard error of measurement was then calculated as $SEM=\sqrt{{\sigma }^{2}*\left(1-\rho \right)}$^16^ where *σ*^2^ represents the total variance in the ICC formula, and *ρ* is the ICC. Finally, the SDC was calculated as $SDC=1.96* \sqrt{2}*SEM$^16^.

The ICC were classified as: poor (< 0.5), moderate (0.5–0.75), good (0.75–0.9), and excellent (> 0.9)^21^. Victor et al.^22^ found a standard deviation around 1 mm and 1 degree for intra and inter-operator measurement error in femoral landmarks and anatomical axes identified with CT scan, the gold-standard method. This is equivalent to a SDC of 2.8 mm/° which led us to define the following classification of SDC levels: poor (> 10 mm/°), moderate (5–10 mm/°), good (3–5 mm/°), excellent (< 3 mm/°).

To refine the understanding of the anatomical landmarks’ measurement errors, paired Student’s t-tests (p < 0.05) were performed between the SDC obtained in different directions (antero-posterior, medial–lateral, vertical). Welch two-sample t-tests were performed between directions and the radiuses (p < 0.05). To evaluate the consistency between different conditions, Student’s t-tests were performed between intra-operator, inter-operator, and test–retest SDC obtained for parameters independent of posture and surgery. Additionally, Student’s t-tests were performed between parameters of the right and left lower limbs pre-surgery to assess potential systematic bias since the lateral source of radiation is on the left-hand side of the patient.

## Results

Values of parameters for all patients are reported in supplementary files S1–S8. Two patients (P03 and P09) presented inversion of condyles on the sagittal view and were excluded from the analysis of the related parameters. Results including these two patients are reported in Supplementary file S9. A graphical overview of the results is presented in Supplementary file S10.

### Anatomical landmarks

The SDCs were good to excellent for most anatomical landmarks apart from the centre of the sacral slope (good to moderate), the anterior superior iliac spines (moderate to poor), and the greater trochanter (moderate to poor) (Table 2).

**Table 2:**
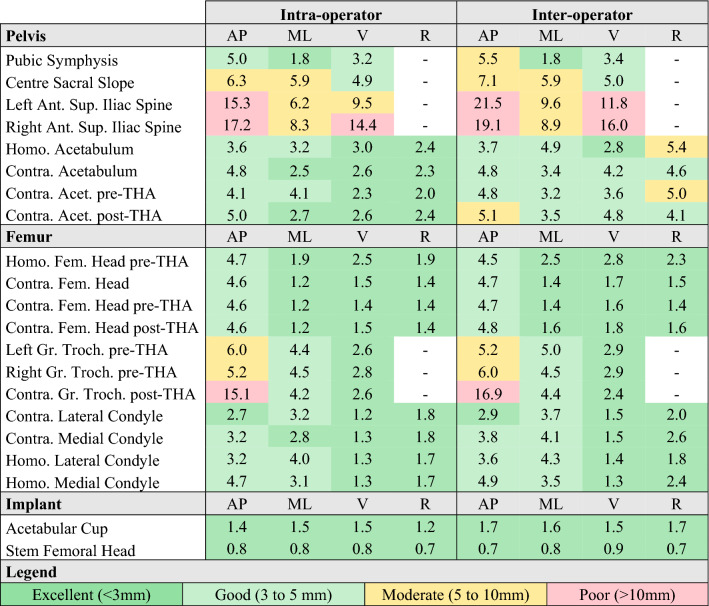
Intra and inter-operator smallest detectable change (SDC) of the position of anatomical points (in mm).

*AP* anterior–posterior direction, *ML* medio-lateral direction, *V* vertical direction, *R* radius, *Ant* anterior, *Sup* superior, *Homo* homolateral side, *Contra* contralateral side, *Acet* acetabulum, *Fem* femoral, *THA* total hip arthroplasty, *Gr. Troch* Greater Trochanter.

The anterior–posterior direction had significantly higher SDC than medio-lateral (p_intra_ = 0.002, p_inter_ = 0.006), vertical (p_intra_ < 0.001, p_inter_ < 0.001) and radiuses (p_intra_ < 0.001, p_inter_ = 0.006) in both intra and inter-operator conditions. Medio-lateral intra-operator SDC was significantly higher than radiuses (p_intra_ = 0.002). Other differences were not significant.

The intra and inter-operator reliability of the position of all anatomical landmarks was excellent (Table 3). The intra-operator reliability of the radius of the condyles was good but the contralateral medial condyle radius had moderate reliability. The radius of the acetabulum had poor inter-operator reliability.

**Table 3:**
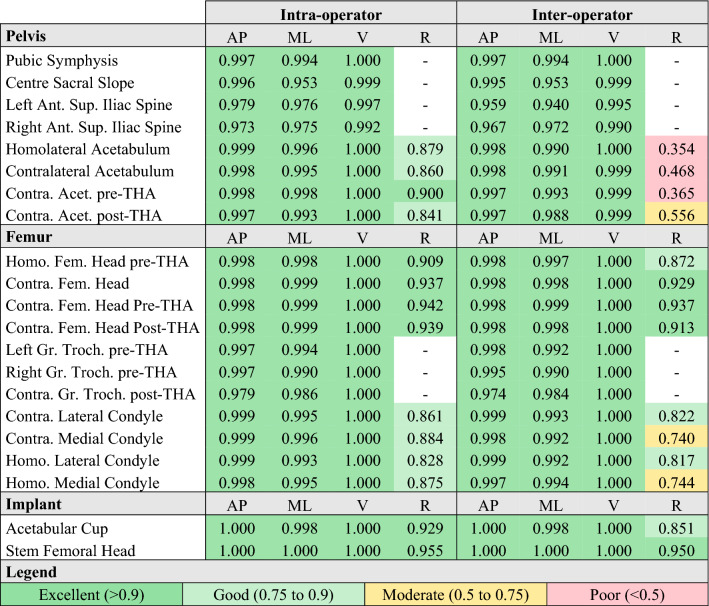
Intra and inter-operator intra-class correlation (ICC) of the position of anatomical points.

*AP* anterior–posterior direction, *ML* medio-lateral direction, *V* vertical direction, *R* radius, *Ant* anterior, *Sup* superior, *Homo* homolateral side, *Contra* contralateral side, *Acet* acetabulum, *Fem* femoral, *THA* total hip arthroplasty, *Gr. Troch* Greater Trochanter.

### Pelvis and femur geometry

Most parameters had good to excellent intra and inter-operator reliability (Tables 4 and 5), apart from APP inclination (moderate inter), femoral torsion and cup inclination (moderate intra and inter), and the diameter of the acetabulum (poor inter). Test–retest reliabilities (Table 4) were good to excellent apart from the cervico-diaphyseal angle, the mechanical angle of the femur, and the femoral torsion (moderate).

**Table 4:**
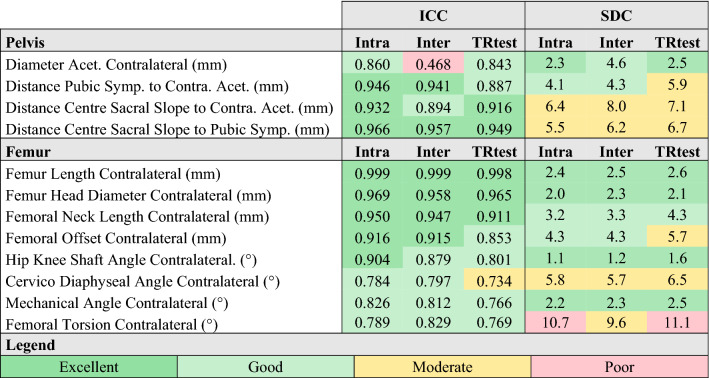
Intra-operator (Intra), intra-operator (Inter) and test–retest (TRtest) ICC and SDC of pelvis and femur geometrical parameters independent of posture and surgery.

*Symp* symphysis, *Contra* contralateral, *Acet* acetabulum.

**Table 5:**
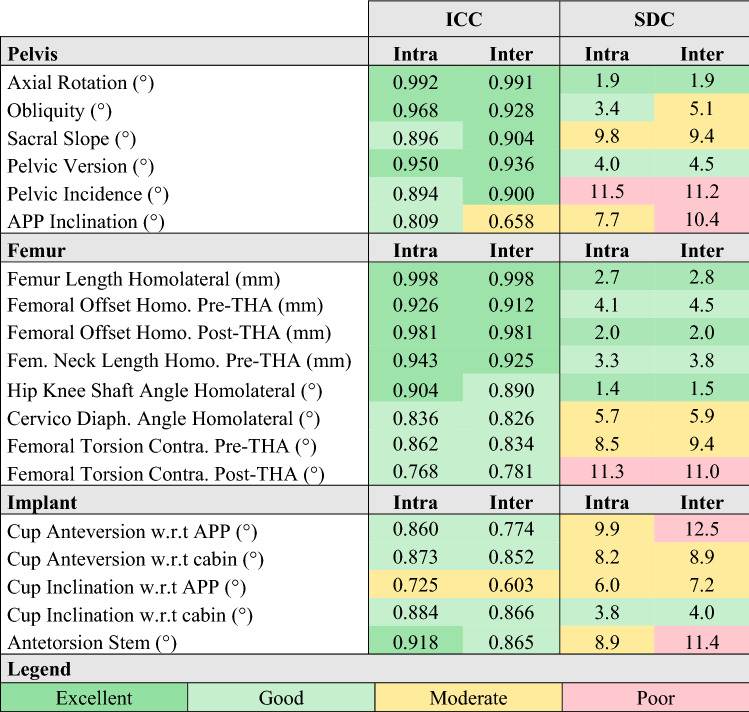
Intra-operator (Intra) and inter-operator (Inter) ICC and SDC of pelvis, femur and implant geometrical parameters dependent of posture and/or surgery.

*APP* anterior pelvic plane, *Homo* homolateral side, *Fem* femoral, *THA* total hip arthroplasty, *Diaph* diaphiseal, *Contra* contralateral, *w.r.t* with respect to.

The SDCs were moderate to excellent for most parameters but the femoral torsion post-THA, and pelvic incidence were poor for both inter and intra-operator conditions while stem antetorsion, cup anteversion with respect to the APP, and APP inclination were poor for the inter-operator condition only. Test–retest SDCs were moderate to excellent apart from the femoral torsion (poor).

### Consistency between conditions

The intra-operator SDCs and ICCs were significantly lower than the test–retest condition (p < 0.001 and p = 0.001 respectively). There were no significant differences in ICCs or SDCs between the inter-operator and test–retest conditions and between the right and left sides (Supplementary file S11).

## Discussion

The aim of this study was to evaluate the reliability and measurement error of the anatomical landmarks and geometry of the pelvis and femur evaluated with bi-plane X-rays in patients before and after THA.

The intra and inter-operator reliabilities were similar to the literature with most ICC above 0.75^8,23,24^, apart from the inter-operator acetabulum radius. This consistency with the literature shows that the level of training of the operators met the standard for such measurements.

The poor inter-operator ICC for the acetabulum radius indicates a slightly different practice between operators for this parameter since intra-operator ICC was good. This indicates that the variability between patients was low, leading to a poor ICC despite a good SDC. Conversely, the pelvic incidence and antero-posterior position of the anterior–superior iliac spines had poor SDC but excellent ICC. This indicates a high variability between patients leading to an excellent ICC despite the poor SDC. Those two results underline the importance of reporting both ICC and SDC for a full understanding of the reliability domain.

Test–retest reliability and measurement error were assessed for the first time with patients and were moderate to excellent, apart from the femoral torsion (poor). They were rated as good as the inter-operator condition, thus, the change in posture of patients between measurements does not seem to have a strong influence on parameters independent of posture, as suggested previously^25,26^. This underlines that bi-plane X-rays are relevant to compare pre- and post-THA measurements.

Likewise, during bi-plane X-rays, the position of the patient in the cabin is standardised and the left lower limb is closer to the source of radiation. This results in a larger magnification of the left side compared to the right side on the images which is often used during reconstruction. There were no differences between parameters assessed on the left and right lower limb which indicates the absence of systematic bias on the measurement error and reliability associated with the side.

The SDC and ICC of the contralateral acetabulum and contralateral femoral head were similar before and after THA. This suggests that the presence of the hip implant does not impede the quality of the reconstruction, apart from cases where the implant would completely overlap with the contralateral hip, and strengthens the validity of the test–retest evaluation presented in this study. To avoid overlapping issues, patients were positioned with one foot slightly forward as recommended^27^. Still, the influence of the implant might not be negligible for all parameters, as seen by the slight increase in SDC for contralateral femoral torsion post-THA and especially with the 10 mm increase in SDC of the greater trochanter anterior–posterior position.

Likewise, there was no difference between the contralateral and homolateral acetabulum and femoral head ICC and SDC before surgery. This suggests that the measurements remain reliable with the presence of coxarthrosis.

The position of the stem femoral head had the best SDC which resulted in higher reliability of the implant’s femoral offset when compared to the native hip.

Anterior superior iliac spines were the least reliable points followed by the centre of the sacral slope indicating that pelvic anatomical landmarks are more difficult to identify than femoral landmarks. These results were expected since the visibility on the pelvis can be low, especially on the sagittal view, due to the superposition of pelvic bone structures, and/or low bone density seen in elderly patients and inherent to this technology. Since the pubic symphysis had good to excellent intra-operator SDC and moderate to excellent inter-operator SDC, the identification of the anterior superior iliac spines seems to be responsible for the difficulties in defining the APP, also reported by Tokunaga et al.^28^.

Positions in the anterior–posterior direction showed significantly higher SDC than in the other two directions. This indicates that the anatomical landmark identification on the sagittal view is more critical than on the frontal view. Errors on this view could be responsible for the poor SDC obtained for APP inclination, pelvic incidence, femoral torsion, and stem antetorsion. Indeed, the normal to the sacral slope, used to compute pelvic incidence, is identified on this view. The poor SDC measured for pelvic incidence was consistent with a previous study that found within-subject standard deviation of 4.12°^23^. Since these parameters characterised the standard error of measurements, it would be equivalent to a within-subject SDC of 11.6°, consistent with the intra- and inter-operator SDC of 11.5° and 11.2° found in the present study. The sacral slope was identified as the main factor for this variability on sagittal X-rays^29^ which is consistent with the SDC of 9.8° and 9.4° measured for this parameter.

Femoral torsion and stem antetorsion had moderate to poor SDC (8.9 to 11.4°). These SDC could be linked to the moderate to poor SDC in the anterior–posterior direction of the greater trochanter. Indeed, the SDC of the femoral torsion was slightly higher after surgery while the SDC of the greater trochanter increased by 10 mm post-surgery. Another candidate could be the anterior–posterior position of the condyles. Although those SDC are good, these points are close to each other in the medial–lateral direction (mean of 48.5 ± 3.7 mm in the present study) and a small position error could lead to a significant angular error. Vanhove et al.^30^ found mean differences of 1.3° between two radiologists for the measurements of femoral torsion with the CT scan based Weiner method. These results are equivalent to an SDC of 3.9° which is lower than our results. Comparisons between bi-plane X-ray measurements and CT scan showed differences of 1.3 ± 6.5° with 31% of patients over 5° of differences^28^, of 4 ± 4°^31^, between − 5 and 7°^32^, and finally limits of agreements of − 5.0 to 4.2°^33^ and − 17.53° to 10.82°^12^. Interestingly, the limits of agreement presented by Cho et al.^12^ have the same order of magnitude as the SDC found in the present study. Correlations between measurement of femoral torsion with CT scan and bi-plane X-rays were found to be strong (R > 0.8)^10,34^ but Mayr et al. showed that for patients with decreased (< 10°) and increased (> 20°) anteversion the correlation was only moderate to low, respectively^34^. Although it was shown that CT scan was influenced by femur position, contrary to bi-plane X-rays^26,34^, the higher measurement error with bi-plane X-rays tempers the recommendation of using this method over CT scan for accurate measurement of femoral torsion. Novel methods of automatic reconstruction of the femur with bi-plane X-rays might improve the reliability and measurement errors of this parameter^35^.

Cup anteversion had moderate to poor SDC (8.2–12.5°). Cup anteversion measured with CT scan was found to have intra- and inter-operator measurement error of 0.52° and 3.2° respectively^36^ corresponding to SDC of 1.5 and 9.0° respectively. Intra and inter-operator SDC with bi-plane X-rays in the present study were higher than SDC reported for CT scans^36^ and seem to contradict a previous study showing equivalence between CT and bi-plane X-rays^7^. However, Journé et al.^7^ performed a comparison on one dry pelvis which represents an ideal case with much better visibility than the one obtained with patients. Comparison of cup inclination and anteversion measured in THA patients with EOS and CT scan showed mean differences below 3° in two studies^28,31^. However, 17% and 34% of patients had more than 5° of difference for inclination and anteversion respectively, indicating that differences at the patient level can be large between CT and EOS measurements^28^. Moreover, limits of agreement of − 5.4° to 6.6° were found in a third study between CT and EOS measurements^33^. Still, correlations above 0.7 were found when comparing cup angles measured with CT and EOS^28^.

Tokunaga et al.^28^ found that stem antetorsion and cup anteversion had almost two times more patients above 5° of difference with CT scans than cup inclination. Interestingly, our results showed that these two first parameters were found to have higher SDC than cup inclination. The larger measurement errors observed in our study could be linked to the larger discrepancy observed by Tokunaga et al.^28^.

Our study focused on reliability and measurement error, but high reliability does not necessarily translate to high validity. As presented in the previous sections, the validity of bi-plane X-rays has been assessed for multiple key parameters. Future studies should continue exploring the validity of this measurement device and compare its reliability and measurement errors to the ones of the gold-standard CT scans.

Despite warnings from the software, two patients presented inversion of condyles on the sagittal view. The error was identified by comparing the multiple reconstructions to each other. Those patients were excluded from the analysis, however, in clinical settings, this error could remain undetected and lead to a biased comparison of pre- and post-THA measurements. Keeping in mind the normal range of femoral torsion may help identify this issue^37^. This outcome underlines the fact that the sagittal view seems to be the main source of error with bi-plane X-rays.

This study has some limitations. The number of patients could have been increased for a higher statistical power, but 80% seems reasonable. Only two operators were considered while the number of operators is frequently larger in a radiological department. Also, the time constraint being lower in research studies than in clinical practice, the present results might represent a best-case scenario. Our study only included patients with end-stage primary osteoarthritis and excluded patients with different pathologies such as severe hip dysplasia, post-traumatic arthritis or post-infection sequelae. Those pathologies can lead to severe deformities of the hip joint and may lead to higher measurement error and lower reliability. Indeed, the identification of structure may be more difficult and those deformities may reach the limits of the generic geometric statistical model used within the reconstruction software. Future studies should explore those limitations and could use the present methodology.

## Conclusion

This study assessed the reliability and measurement error of anatomical landmark position and standard geometrical parameters for the pelvis and femur measured by bi-plane X-rays (EOS system). The sagittal view had significantly higher measurement errors than the frontal view. Osteoarthritis and the presence of an implant on the lateral view did not seem to have a strong influence on reliability and measurement errors. There were no significant differences between the test–retest and inter-operator reliability and measurement error which suggests that this technology is suitable to compare pre- and post-THA parameters.

Pelvic incidence and position of the anterior superior iliac spines had poor inter and intra-operator measurement errors that could be linked to higher errors on the sagittal view. The femoral and stem torsion, cup anteversion, and inclination had moderate to poor measurement errors. Interestingly, based on the literature, high reliability is challenging with CT scan for those parameters. Parameters with good SDC and poor ICC and vice-versa shows the importance of considering the whole domain of reliability to fully understand the measurement.

This study can help radiographers better understand their tool and define more accurate guidelines for parameters with lower reliability. It can also be used by clinicians when analysing bi-plane X-rays and in future longitudinal studies using this technology to assess the structure of the lower limb.

## Supplementary Information

Supplementary Information 1.

Supplementary Information 2.

Supplementary Information 3.

Supplementary Information 4.

Supplementary Information 5.

Supplementary Information 6.

Supplementary Information 7.

Supplementary Information 8.

Supplementary Information 9.

Supplementary Information 10.

Supplementary Information 11.

## Data Availability

Data is available on Yareta: https://doi.org/10.26037/yareta:2al7pd6y7rgkzkd6nolwp6ucji.

## References

[1] Mokkink LB (2010). The COSMIN checklist for assessing the methodological quality of studies on measurement properties of health status measurement instruments: An international Delphi study. Qual. Life Res..

[2] Ilharreborde B, Ferrero E, Alison M, Mazda K (2016). EOS microdose protocol for the radiological follow-up of adolescent idiopathic scoliosis. Eur. Spine J..

[3] Bittershol B (2013). EOS imaging of the human pelvis: Reliability, validity, and controlled comparison with radiography. J. Bone Joint Surg..

[4] Rousseau M-A, Brusson A, Lazennec J-Y (2014). Assessment of the axial rotation of the pelvis with the EOS® imaging system: Intra- and inter-observer reproducibility and accuracy study. Eur. J. Orthop. Surg. Traumatol..

[5] Ghostine B (2017). Influence of patient axial malpositioning on the trueness and precision of pelvic parameters obtained from 3D reconstructions based on biplanar radiographs. Eur. Radiol..

[6] Barbier O, Skalli W, Mainard L, Mainard D (2014). The reliability of the anterior pelvic plane for computer navigated acetabular component placement during total hip arthroplasty: Prospective study with the EOS imaging system. Orthop. Traumatol. Surg. Res..

[7] Journé A, Sadaka J, Bélicourt C, Sautet A (2012). New method for measuring acetabular component positioning with EOS imaging: Feasibility study on dry bone. Int. Orthop..

[8] Guenoun B, Zadegan F, Aim F, Hannouche D, Nizard R (2012). Reliability of a new method for lower-extremity measurements based on stereoradiographic three-dimensional reconstruction. Orthop. Traumatol. Surg. Res..

[9] Escott BG (2013). EOS low-dose radiography: A reliable and accurate upright assessment of lower-limb lengths. J. Bone Joint Surg. Am..

[10] Folinais D (2013). Measuring femoral and rotational alignment: EOS system versus computed tomography. Orthop. Traumatol. Surg. Res..

[11] Pomerantz ML, Glaser D, Doan J, Kumar S, Edmonds EW (2015). Three-dimensional biplanar radiography as a new means of accessing femoral version: A comparitive study of EOS three-dimensional radiography versus computed tomography. Skelet. Radiol..

[12] Cho BW (2021). Evaluation of the reliability of lower extremity alignment measurements using EOS imaging system while standing in an even weight-bearing posture. Sci. Rep..

[13] Lazennec JY, Brusson A, Dominique F, Rousseau MA, Pour AE (2015). Offset and anteversion reconstruction after cemented and uncemented total hip arthroplasty: an evaluation with the low-dose EOS system comparing two- and three-dimensional imaging. Int. Orthop..

[14] Bujang MA, Baharum N (2017). A simplified guide to determination of sample size requirements for estimating the value of intraclass correlation coefficient: A review. Arch. Orofac. Sci..

[15] Chia K, Sangeux M (2017). Quantifying sources of variability in gait analysis. Gait Posture.

[16] De Vet, H. C., Terwee, C. B., Mokkink, L. B. & Knol, D. L. *Measurement in medicine: a practical guide*. (Cambridge university press, 2011).

[17] McGraw KO, Wong SP (1996). Forming inferences about some intraclass correlation coefficients. Psychol. Methods.

[18] Bates D, Mächler M, Bolker B, Walker S (2015). fitting linear mixed-effects models using lme4. J. Stat. Softw..

[19] R: A Language and Environment for Statistical Computing (R Foundation for Statistical Computing, 2021).

[20] Van Lummel RC (2016). Intra-Rater, inter-rater and test-retest reliability of an instrumented timed up and go (iTUG) Test in patients with Parkinson’s disease. PLoS ONE.

[21] Koo TK, Li MY (2016). A guideline of selecting and reporting intraclass correlation coefficients for reliability research. J. Chiropr. Med..

[22] Victor J (2009). How precise can bony landmarks be determined on a CT scan of the knee?. Knee.

[23] Demzik AL (2016). Inter-rater and intra-rater repeatability and reliability of EOS 3-dimensional imaging analysis software. J. Arthroplasty.

[24] Clavé A (2018). Reproducibility of length measurements of the lower limb by using EOS™. Musculoskelet. Surg..

[25] Melhem E, Assi A, El Rachkidi R, Ghanem I (2016). EOS(®) biplanar X-ray imaging: Concept, developments, benefits, and limitations. J. Child. Orthop..

[26] Morvan G, Guerini H, Carré G, Vuillemin V (2017). Femoral torsion: Impact of femur position on CT and stereoradiography measurements. Am. J. Roentgenol..

[27] Chaibi Y (2012). Fast 3D reconstruction of the lower limb using a parametric model and statistical inferences and clinical measurements calculation from biplanar X-rays. Comput. Methods Biomech. Biomed. Engin..

[28] Tokunaga K, Okamoto M, Watanabe K (2018). Implant orientation measurement after THA using the EOS X-ray image acquisition system. Adv. Exp. Med. Biol..

[29] Yamada K (2015). Accuracies in measuring spinopelvic parameters in full-spine lateral standing radiograph. Spine.

[30] Vanhove F (2019). Standardization of torsional CT measurements of the lower limbs with threshold values for corrective osteotomy. Arch. Orthop. Trauma Surg..

[31] Esposito CI (2020). Biplanar low-dose radiography is accurate for measuring combined anteversion after total hip arthroplasty. HSS J..

[32] Buck FM, Guggenberger R, Koch PP, Pfirrmann CWA (2012). Femoral and tibial torsion measurements with 3D models based on low-dose biplanar radiographs in comparison with standard CT measurements. Am. J. Roentgenol..

[33] Ma Z (2022). Assessing component orientation of total hip arthroplasty using the low-dose bi-planar radiographs. BMC Musculoskelet. Disord..

[34] Mayr HO (2021). Anteversion angle measurement in suspected torsional malalignment of the femur in 3-dimensional EOS vs computed tomography—a validation study. J. Arthroplasty.

[35] Girinon F (2020). Quasi-automated reconstruction of the femur from bi-planar X-rays. Comput. Methods Biomech. Biomed. Eng. Imaging Vis..

[36] Ghelman B, Kepler CK, Lyman S, DellaValle AG (2009). CT outperforms radiography for determination of acetabular cup version after THA. Clin. Orthopaed. Relat. Res..

[37] De Pieri E (2021). Subject-specific modeling of femoral torsion influences the prediction of hip loading during gait in asymptomatic adults. Front. Bioeng. Biotechnol..

