# Supplementary material for: Reliability of the pelvis and femur anatomical landmarks and geometry with the EOS system before and after total hip arthroplasty
Source: Sci Rep. 2022 Dec 11;12:21420. doi: 10.1038/s41598-022-25997-3 (PMC9742167; doi:10.1038/s41598-022-25997-3)
Supplement: Supplementary file 1 — Supplementary Information 1. [file 41598_2022_25997_MOESM1_ESM.pdf]

Anatomical Points of the Pelvis

- **Center Sacral Slope** (p.2-4)
- **Contralateral Cotyle** (p.5-8)
- **Homolateral Cotyle** (p.9-12)
- **Left Anterior Superior Iliac Spine** (p.13-15)
- **Pubic Symphisis** (p.16-18)
- **Right Anterior Superior Iliac Spine** (p.19-21)

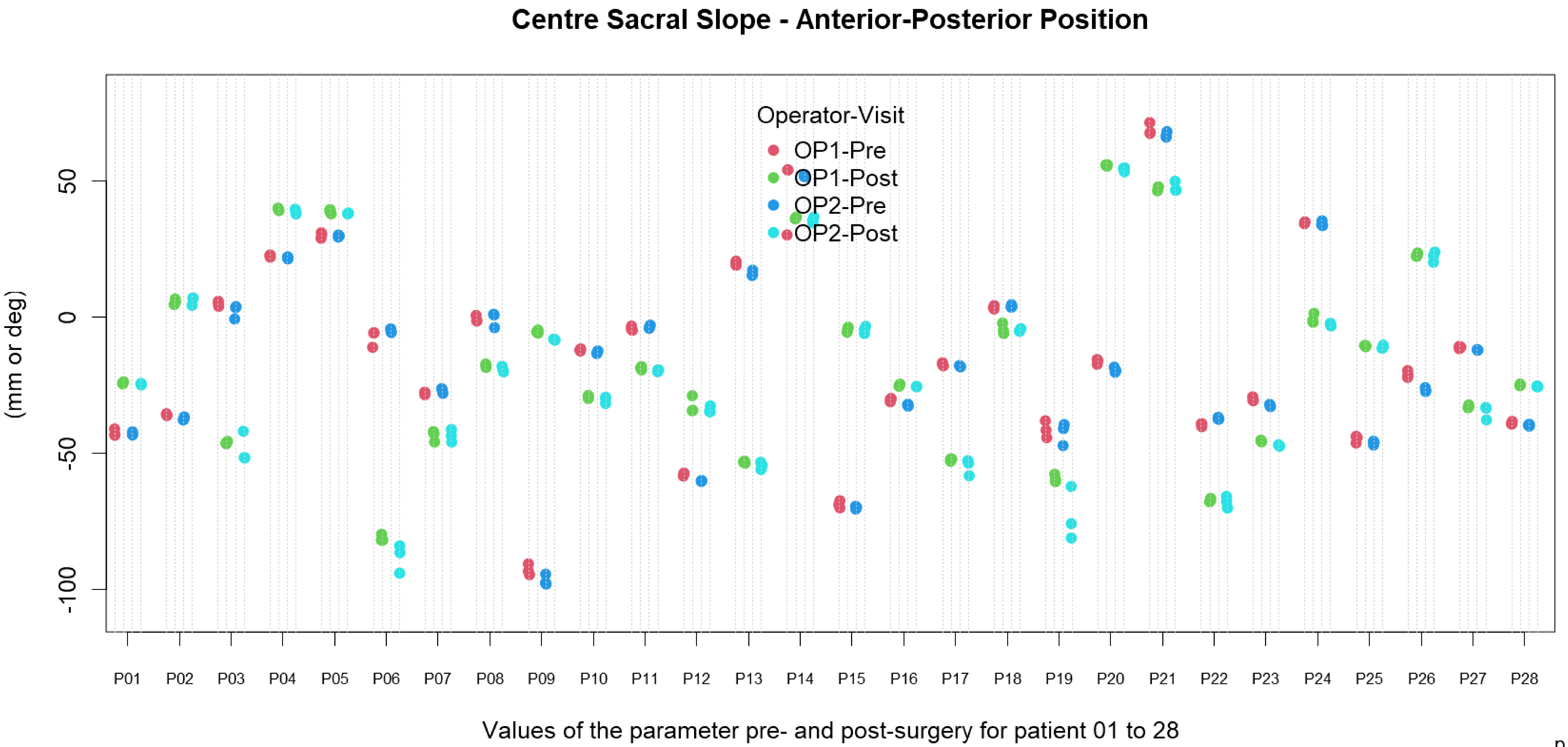
Centre Sacral Slope - Anterior-Posterior Position
Operator-Visit
OP1-Pre
OP1-Post
OP2-Pre
OP2-Post
(mm or deg)
50
0
-50
-100
P01
P02
P03
P04
P05
P06
P07
P08
P09
P10
P11
P12
P13
P14
P15
P16
P17
P18
P19
P20
P21
P22
P23
P24
P25
P26
P27
P28
Values of the parameter pre- and post-surgery for patient 01 to 28

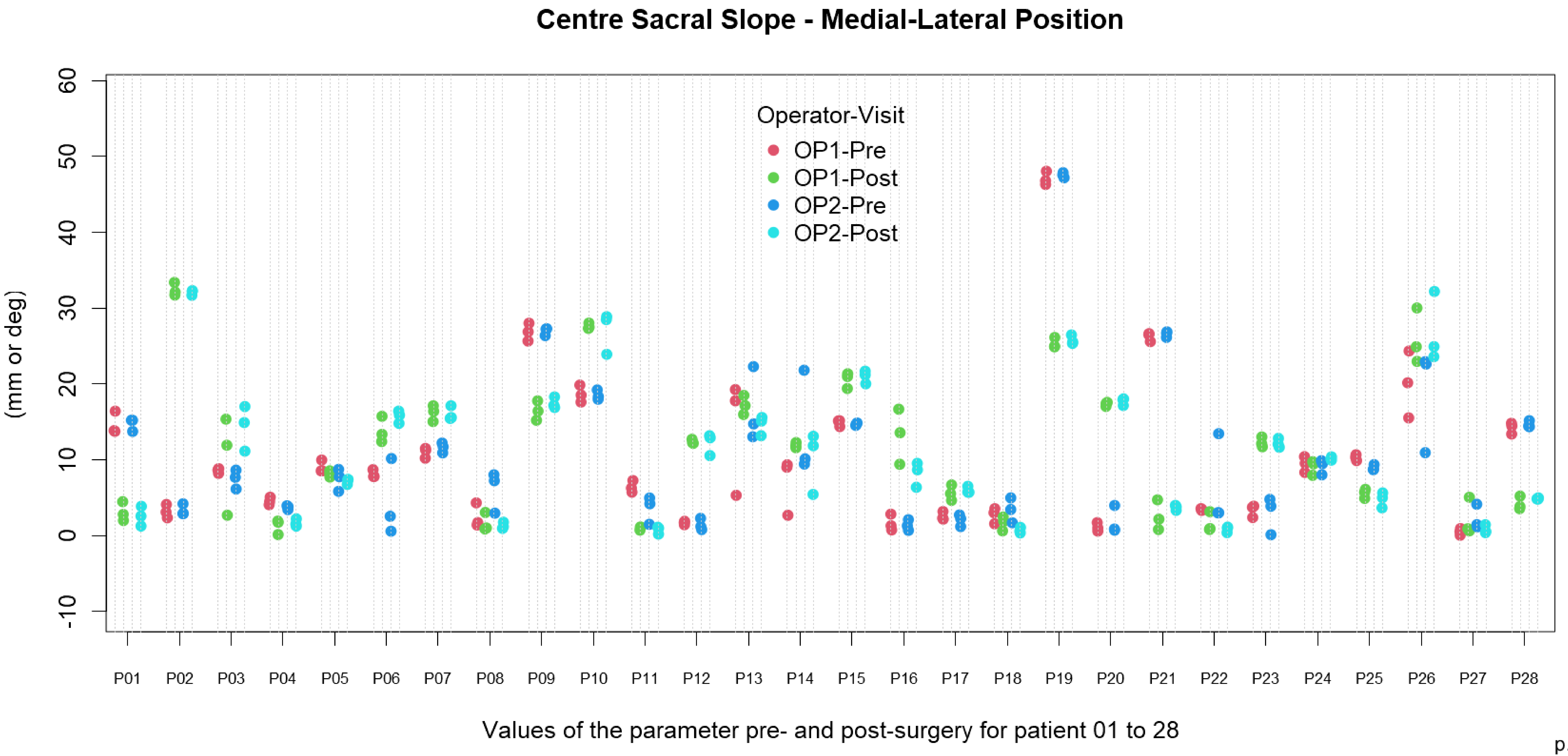

Centre Sacral Slope - Medial-Lateral Position
Operator-Visit
OP1-Pre
OP1-Post
OP2-Pre
OP2-Post
(mm or deg)
-10
0
10
20
30
40
50
60
P01
P02
P03
P04
P05
P06
P07
P08
P09
P10
P11
P12
P13
P14
P15
P16
P17
P18
P19
P20
P21
P22
P23
P24
P25
P26
P27
P28
Values of the parameter pre- and post-surgery for patient 01 to 28

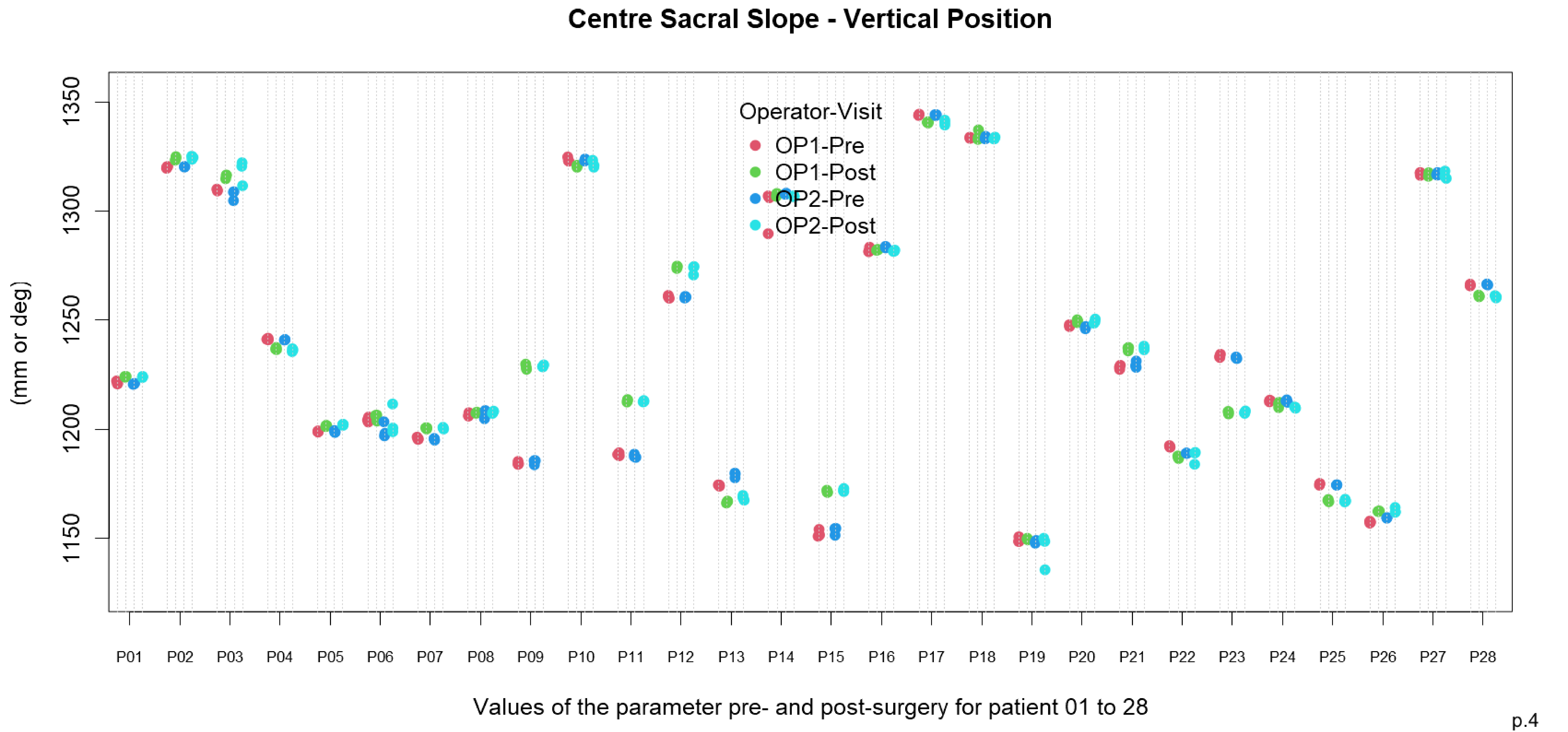
Centre Sacral Slope - Vertical Position
Operator-Visit
OP1-Pre
OP1-Post
OP2-Pre
OP2-Post
(mm or deg)
1150
1200
1250
1300
1350
P01
P02
P03
P04
P05
P06
P07
P08
P09
P10
P11
P12
P13
P14
P15
P16
P17
P18
P19
P20
P21
P22
P23
P24
P25
P26
P27
P28
Values of the parameter pre- and post-surgery for patient 01 to 28

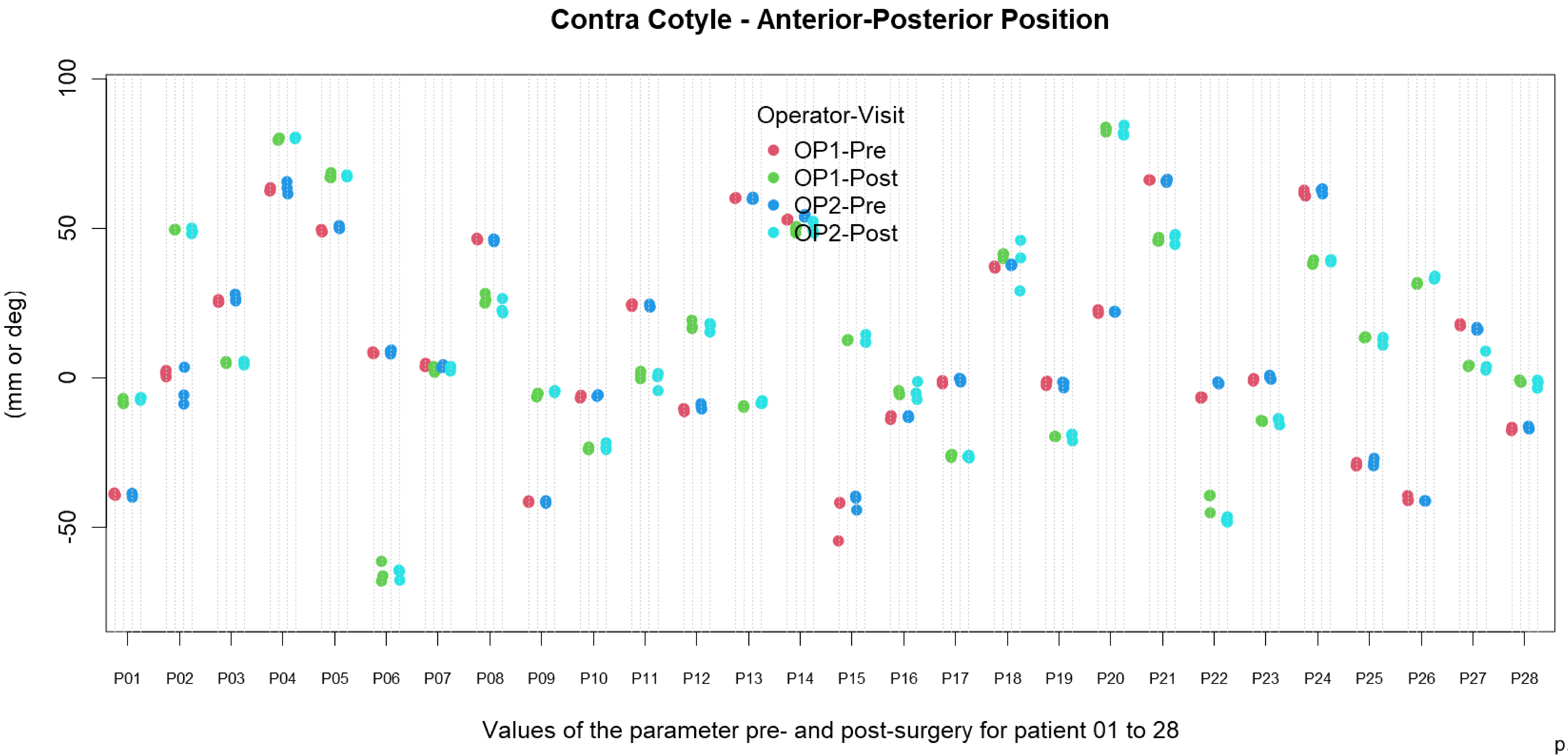
Contra Cotyle - Anterior-Posterior Position
Operator-Visit
OP1-Pre
OP1-Post
OP2-Pre
OP2-Post
100
50
0
-50
(mm or deg)
P01
P02
P03
P04
P05
P06
P07
P08
P09
P10
P11
P12
P13
P14
P15
P16
P17
P18
P19
P20
P21
P22
P23
P24
P25
P26
P27
P28
Values of the parameter pre- and post-surgery for patient 01 to 28

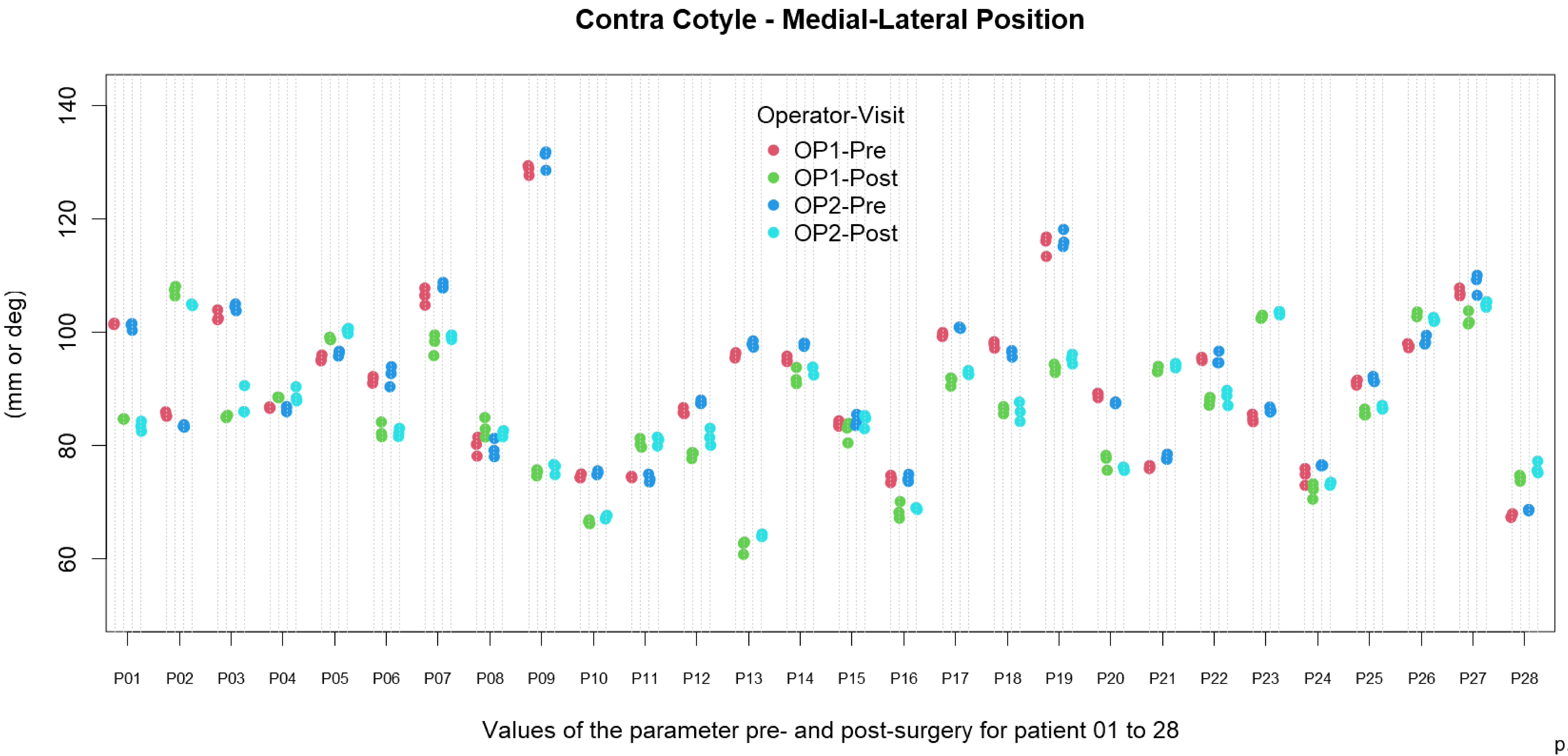

Contra Cotyle - Medial-Lateral Position
Operator-Visit
OP1-Pre
OP1-Post
OP2-Pre
OP2-Post
(mm or deg)
60
80
100
120
140
P01
P02
P03
P04
P05
P06
P07
P08
P09
P10
P11
P12
P13
P14
P15
P16
P17
P18
P19
P20
P21
P22
P23
P24
P25
P26
P27
P28
Values of the parameter pre- and post-surgery for patient 01 to 28

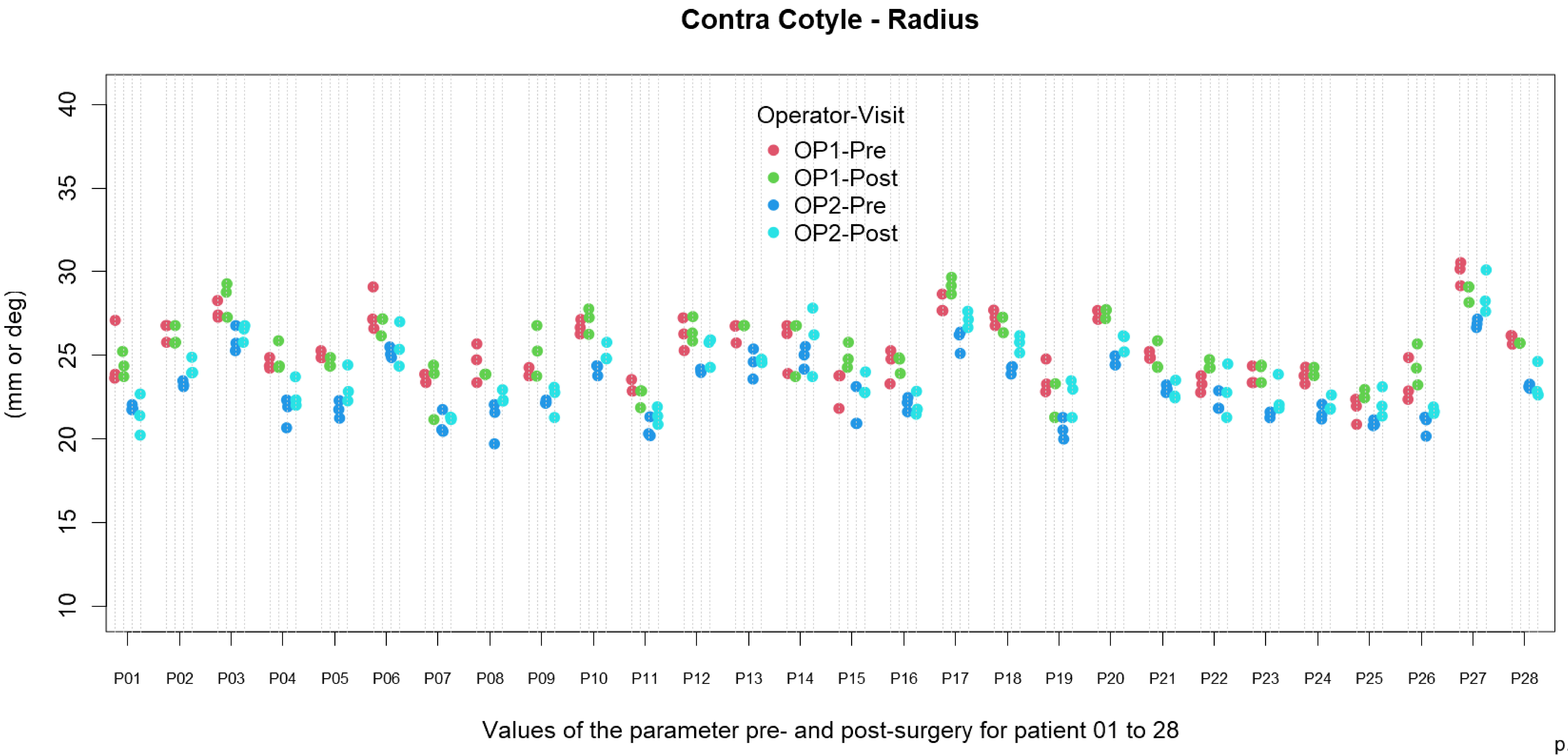
Contra Cotyle - Radius
Operator-Visit
OP1-Pre
OP1-Post
OP2-Pre
OP2-Post
(mm or deg)
10
15
20
25
30
35
40
P01
P02
P03
P04
P05
P06
P07
P08
P09
P10
P11
P12
P13
P14
P15
P16
P17
P18
P19
P20
P21
P22
P23
P24
P25
P26
P27
P28
Values of the parameter pre- and post-surgery for patient 01 to 28

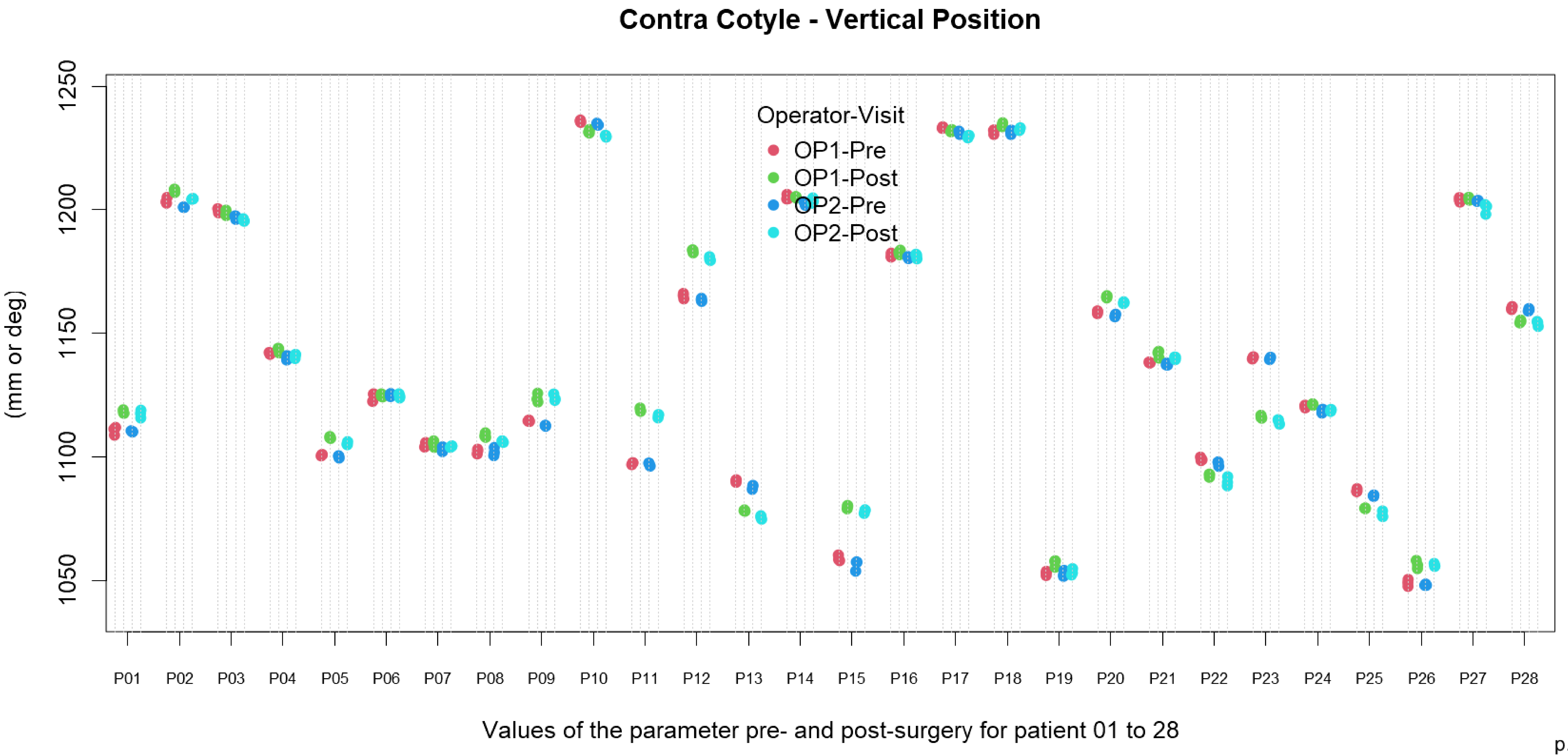
Contra Cotyle - Vertical Position
Operator-Visit
OP1-Pre
OP1-Post
OP2-Pre
OP2-Post
1250
1200
1150
1100
1050
(mm or deg)
P01 P02 P03 P04 P05 P06 P07 P08 P09 P10 P11 P12 P13 P14 P15 P16 P17 P18 P19 P20 P21 P22 P23 P24 P25 P26 P27 P28
Values of the parameter pre- and post-surgery for patient 01 to 28

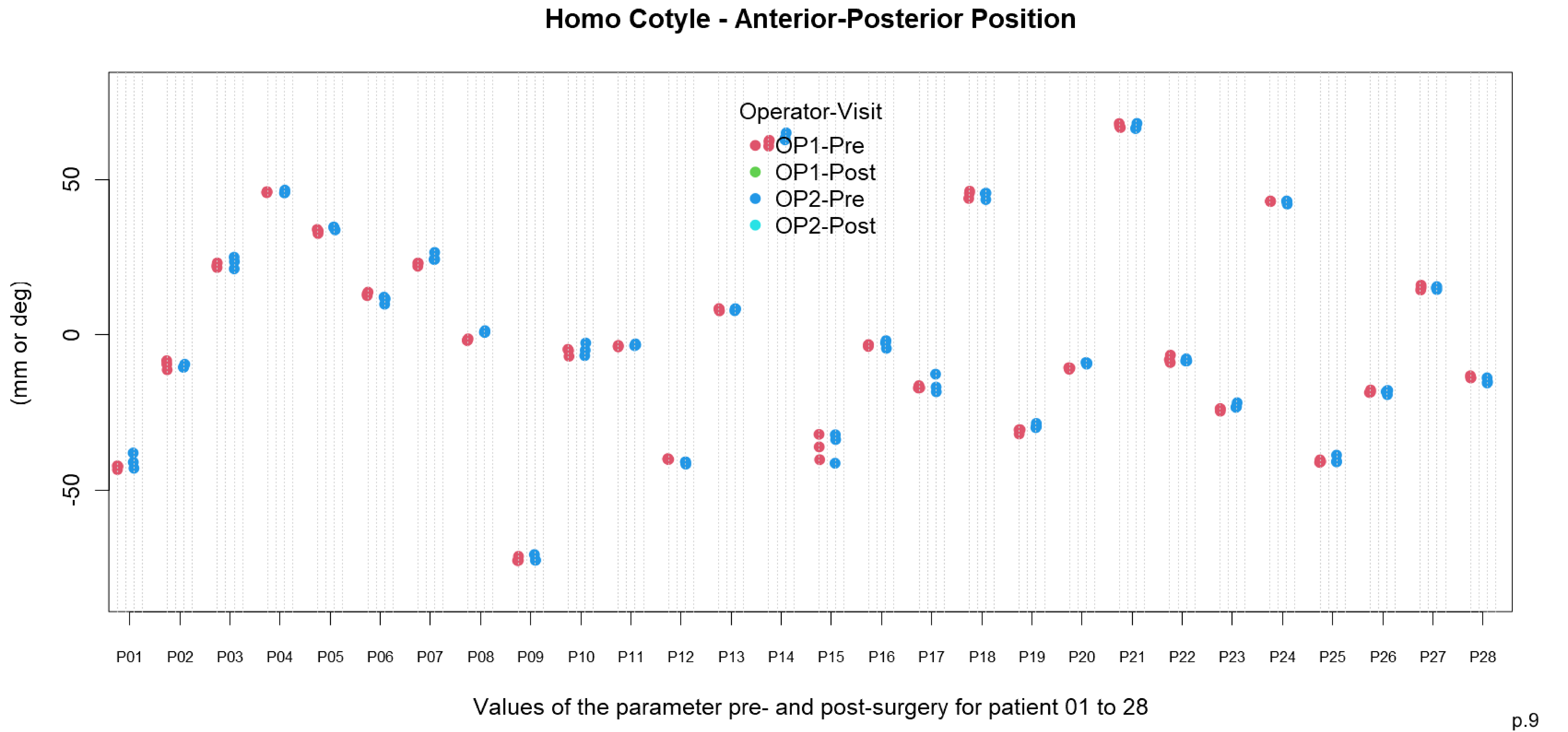
Homo Cotyle - Anterior-Posterior Position
Operator-Visit
OP1-Pre
OP1-Post
OP2-Pre
OP2-Post
50
0
-50
(mm or deg)
P01
P02
P03
P04
P05
P06
P07
P08
P09
P10
P11
P12
P13
P14
P15
P16
P17
P18
P19
P20
P21
P22
P23
P24
P25
P26
P27
P28
Values of the parameter pre- and post-surgery for patient 01 to 28

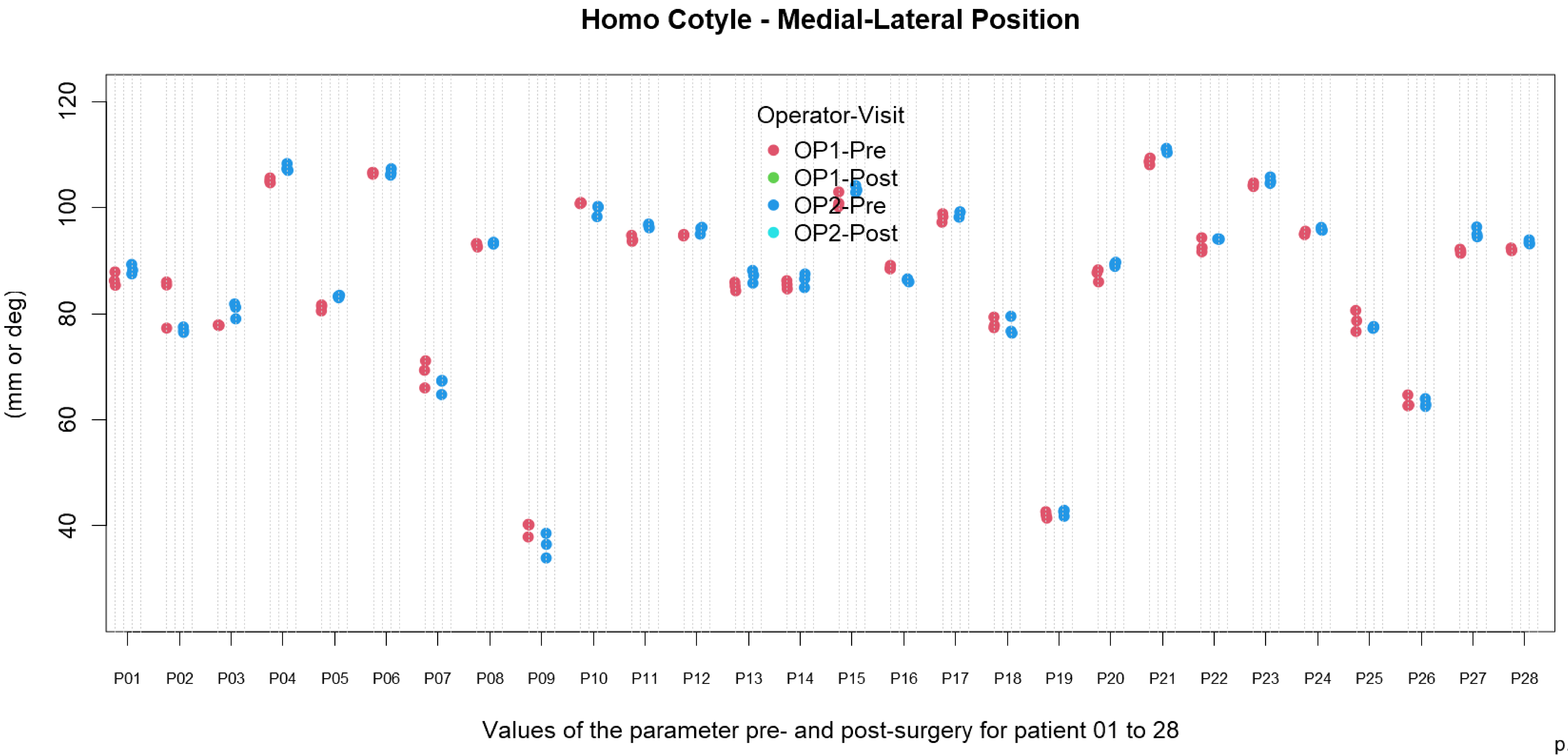
Homo Cotyle - Medial-Lateral Position
Operator-Visit
OP1-Pre
OP1-Post
OP2-Pre
OP2-Post
(mm or deg)
120
100
80
60
40
P01
P02
P03
P04
P05
P06
P07
P08
P09
P10
P11
P12
P13
P14
P15
P16
P17
P18
P19
P20
P21
P22
P23
P24
P25
P26
P27
P28
Values of the parameter pre- and post-surgery for patient 01 to 28

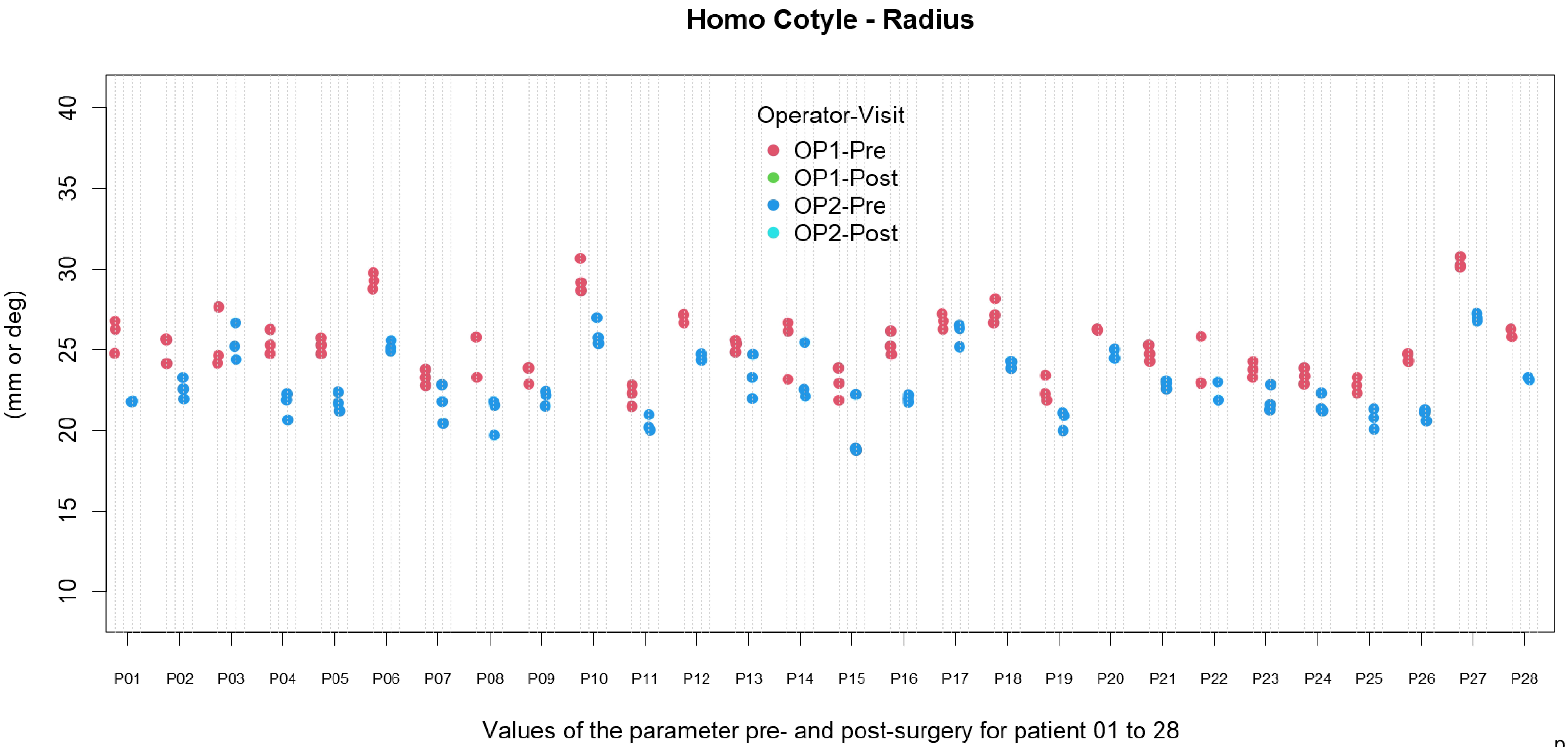
Homo Cotyle - Radius
Operator-Visit
OP1-Pre
OP1-Post
OP2-Pre
OP2-Post
(mm or deg)
10
15
20
25
30
35
40
P01 P02 P03 P04 P05 P06 P07 P08 P09 P10 P11 P12 P13 P14 P15 P16 P17 P18 P19 P20 P21 P22 P23 P24 P25 P26 P27 P28
Values of the parameter pre- and post-surgery for patient 01 to 28

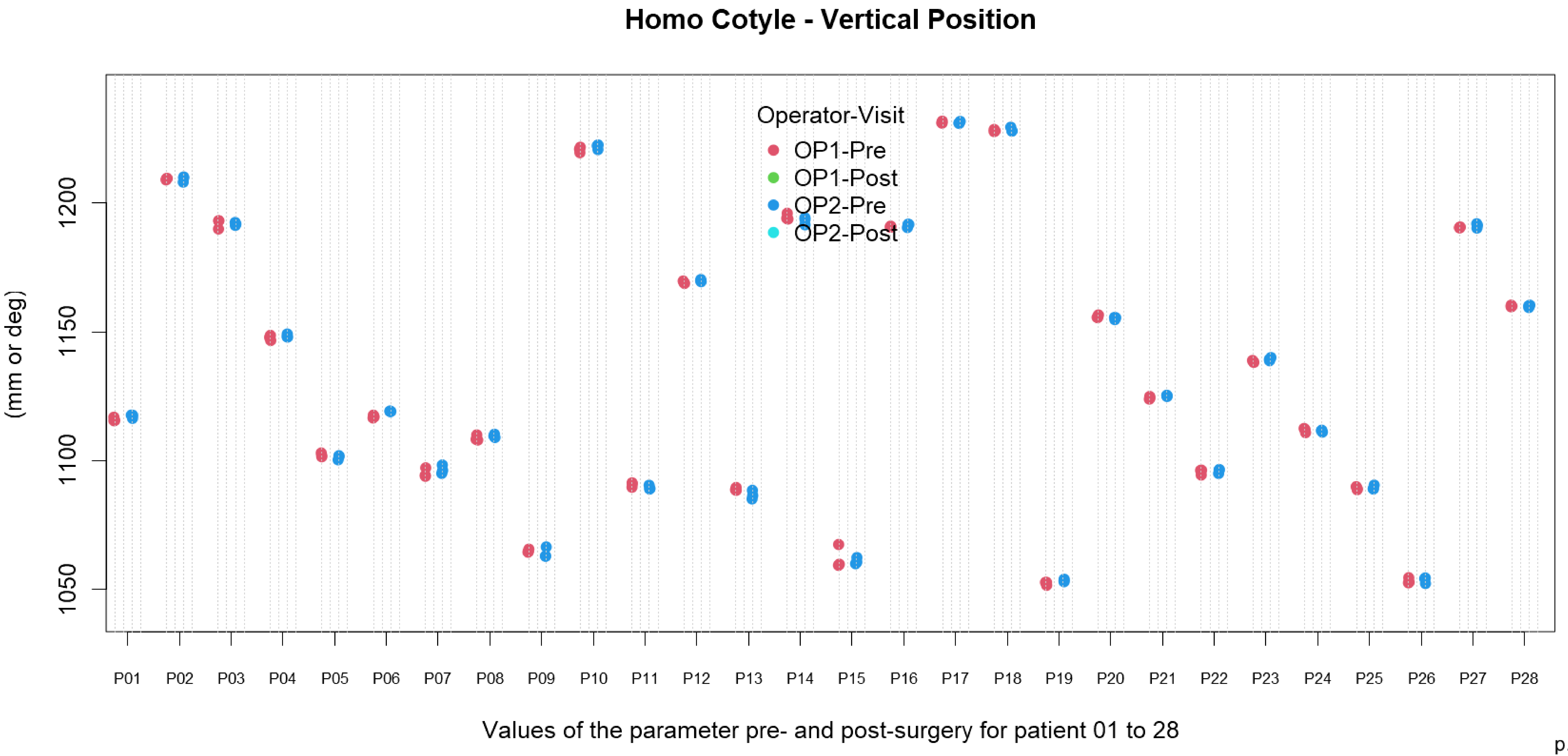

Homo Cotyle - Vertical Position
Operator-Visit
OP1-Pre
OP1-Post
OP2-Pre
OP2-Post
(mm or deg)
1050
1100
1150
1200
P01
P02
P03
P04
P05
P06
P07
P08
P09
P10
P11
P12
P13
P14
P15
P16
P17
P18
P19
P20
P21
P22
P23
P24
P25
P26
P27
P28
Values of the parameter pre- and post-surgery for patient 01 to 28

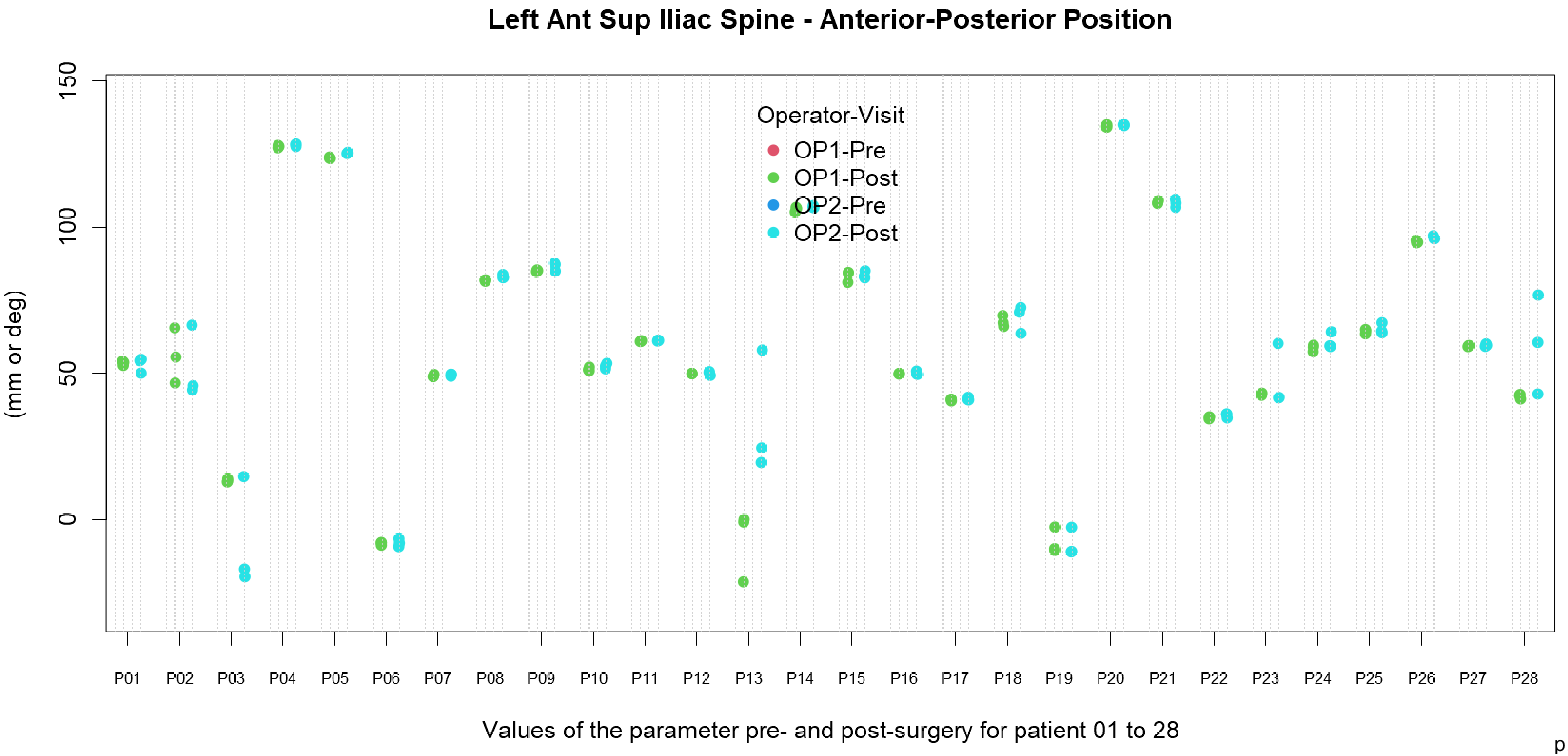

Left Ant Sup Iliac Spine - Anterior-Posterior Position
Operator-Visit
OP1-Pre
OP1-Post
OP2-Pre
OP2-Post
(mm or deg)
150
100
50
0
P01
P02
P03
P04
P05
P06
P07
P08
P09
P10
P11
P12
P13
P14
P15
P16
P17
P18
P19
P20
P21
P22
P23
P24
P25
P26
P27
P28
Values of the parameter pre- and post-surgery for patient 01 to 28

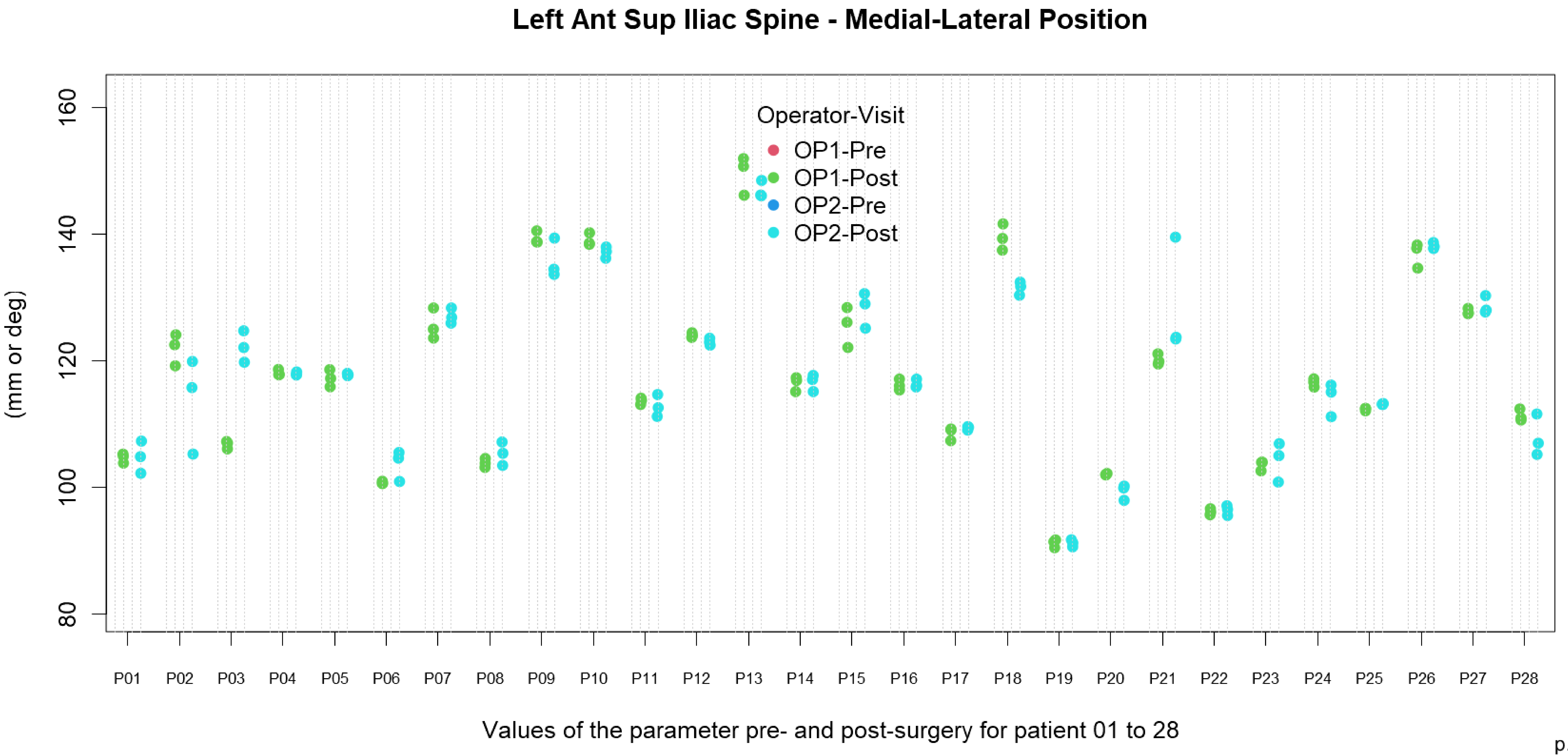

Left Ant Sup Iliac Spine - Medial-Lateral Position
Operator-Visit
OP1-Pre
OP1-Post
OP2-Pre
OP2-Post
(mm or deg)
80
100
120
140
160
P01
P02
P03
P04
P05
P06
P07
P08
P09
P10
P11
P12
P13
P14
P15
P16
P17
P18
P19
P20
P21
P22
P23
P24
P25
P26
P27
P28
Values of the parameter pre- and post-surgery for patient 01 to 28

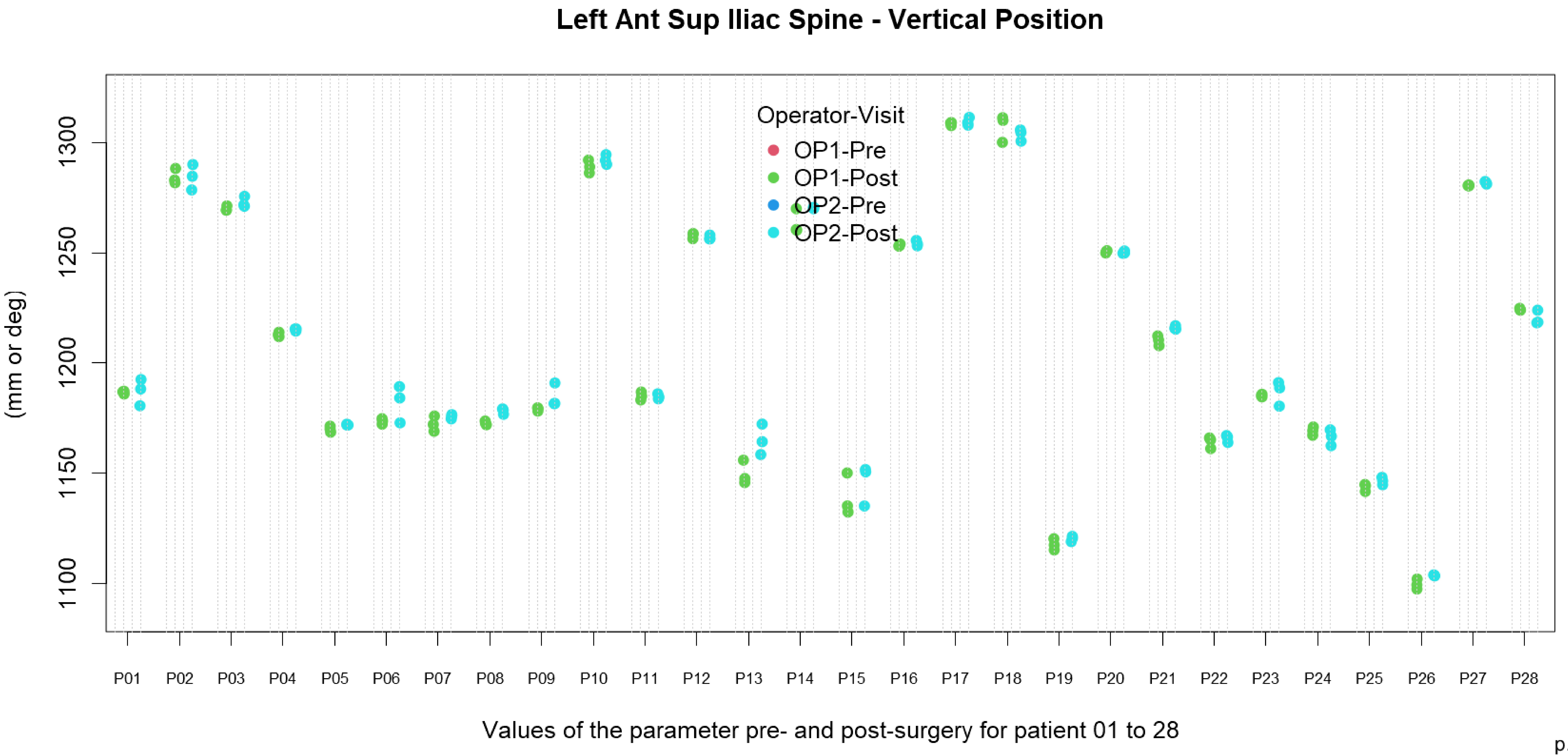

Left Ant Sup Iliac Spine - Vertical Position
(mm or deg)
1100
1150
1200
1250
1300
Operator-Visit
OP1-Pre
OP1-Post
OP2-Pre
OP2-Post
P01
P02
P03
P04
P05
P06
P07
P08
P09
P10
P11
P12
P13
P14
P15
P16
P17
P18
P19
P20
P21
P22
P23
P24
P25
P26
P27
P28
Values of the parameter pre- and post-surgery for patient 01 to 28

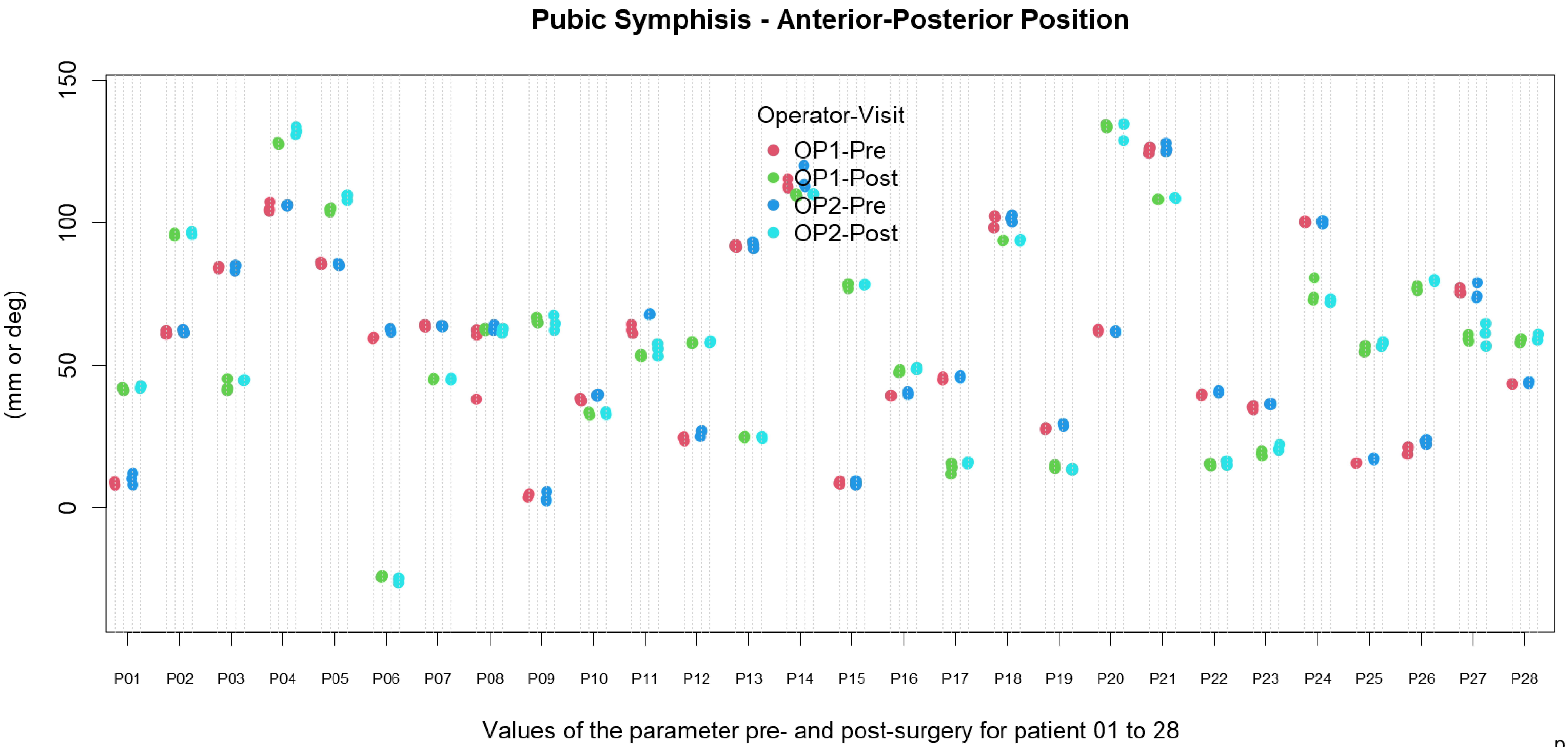

Pubic Symphisis - Anterior-Posterior Position
Operator-Visit
OP1-Pre
OP1-Post
OP2-Pre
OP2-Post
(mm or deg)
150
100
50
0
P01
P02
P03
P04
P05
P06
P07
P08
P09
P10
P11
P12
P13
P14
P15
P16
P17
P18
P19
P20
P21
P22
P23
P24
P25
P26
P27
P28
Values of the parameter pre- and post-surgery for patient 01 to 28

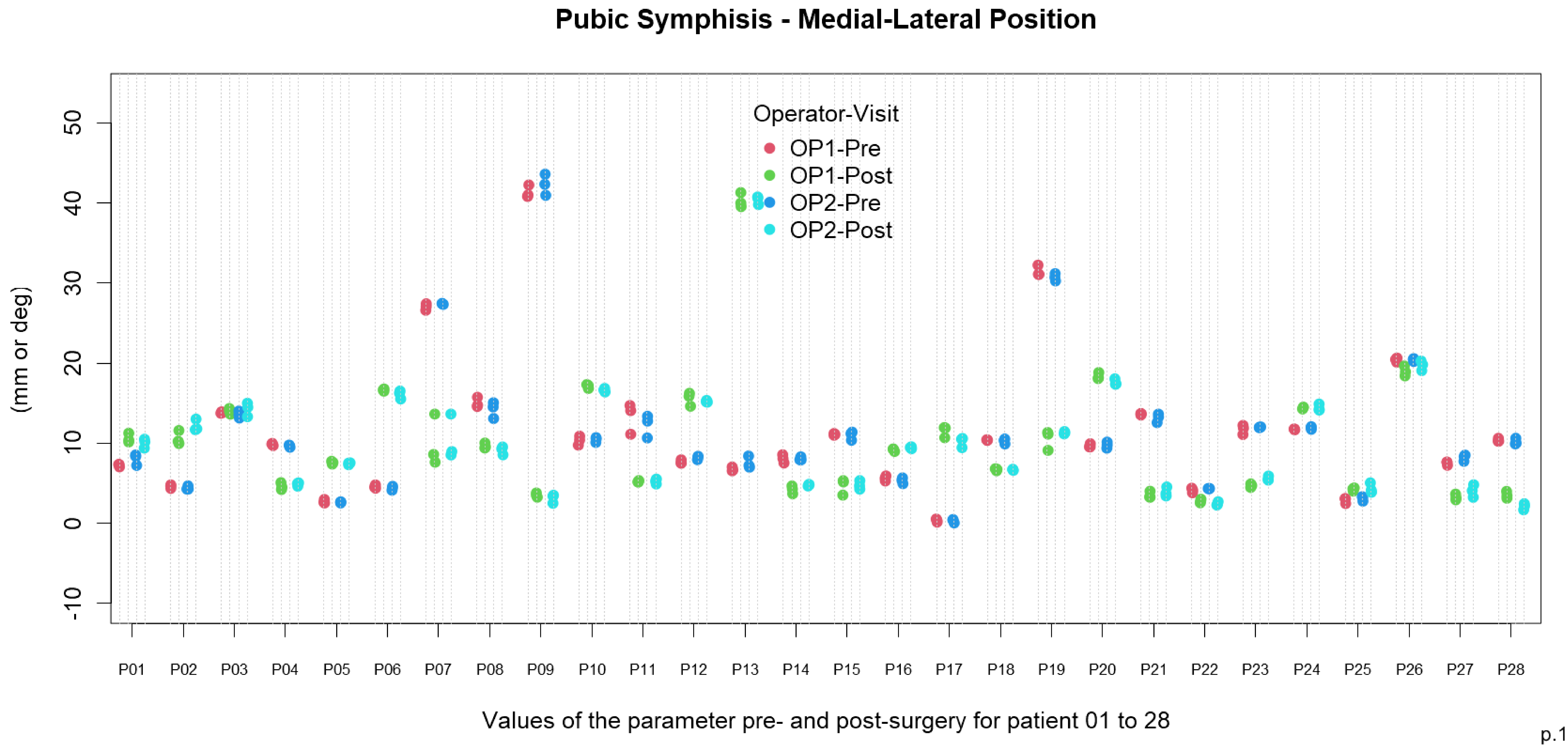
Pubic Symphisis - Medial-Lateral Position
Operator-Visit
OP1-Pre
OP1-Post
OP2-Pre
OP2-Post
(mm or deg)
-10
0
10
20
30
40
50
P01
P02
P03
P04
P05
P06
P07
P08
P09
P10
P11
P12
P13
P14
P15
P16
P17
P18
P19
P20
P21
P22
P23
P24
P25
P26
P27
P28
Values of the parameter pre- and post-surgery for patient 01 to 28

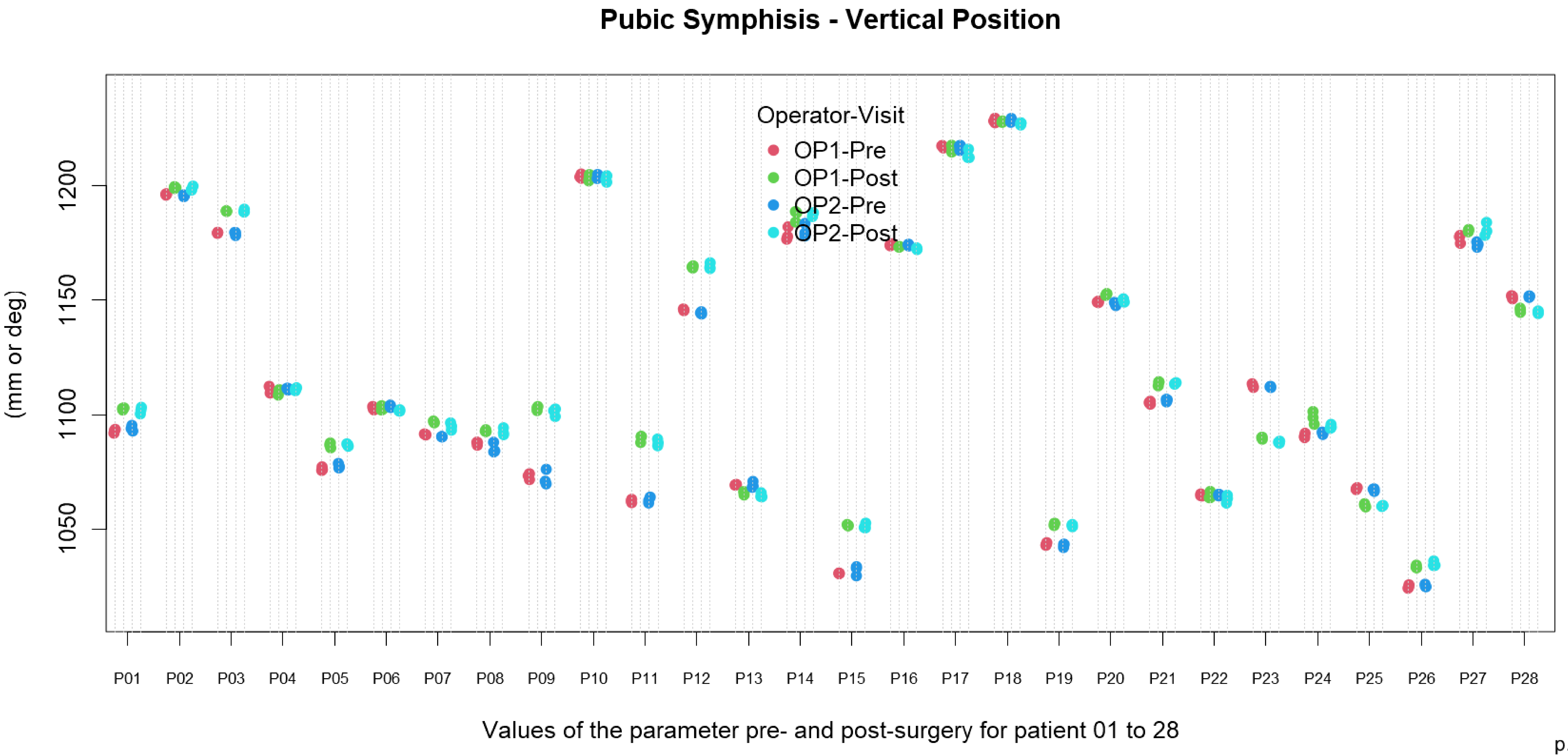

Pubic Symphisis - Vertical Position
Operator-Visit
OP1-Pre
OP1-Post
OP2-Pre
OP2-Post
(mm or deg)
1050
1100
1150
1200
P01
P02
P03
P04
P05
P06
P07
P08
P09
P10
P11
P12
P13
P14
P15
P16
P17
P18
P19
P20
P21
P22
P23
P24
P25
P26
P27
P28
Values of the parameter pre- and post-surgery for patient 01 to 28

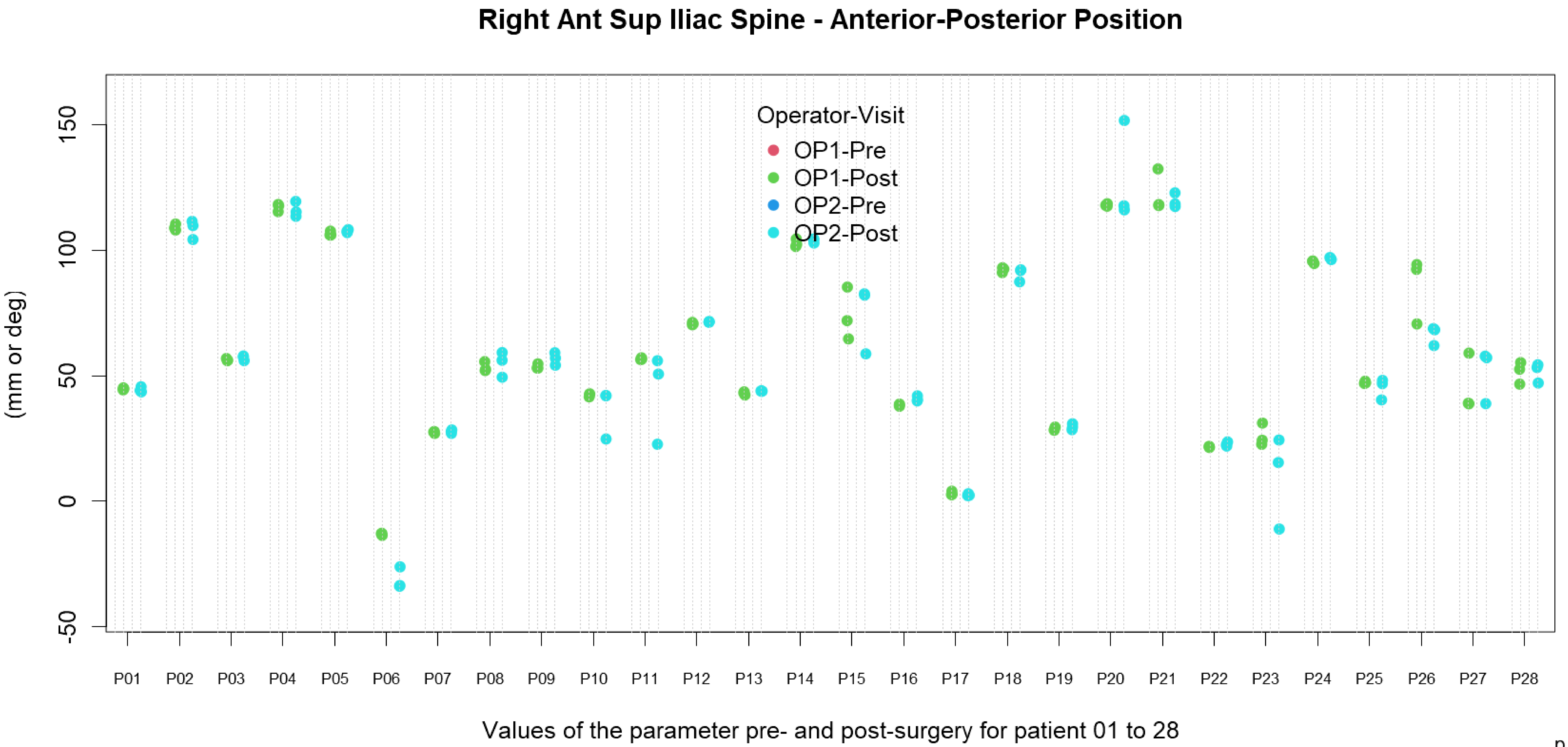
Right Ant Sup Iliac Spine - Anterior-Posterior Position
Operator-Visit
OP1-Pre
OP1-Post
OP2-Pre
OP2-Post
(mm or deg)
150
100
50
0
-50
P01
P02
P03
P04
P05
P06
P07
P08
P09
P10
P11
P12
P13
P14
P15
P16
P17
P18
P19
P20
P21
P22
P23
P24
P25
P26
P27
P28
Values of the parameter pre- and post-surgery for patient 01 to 28

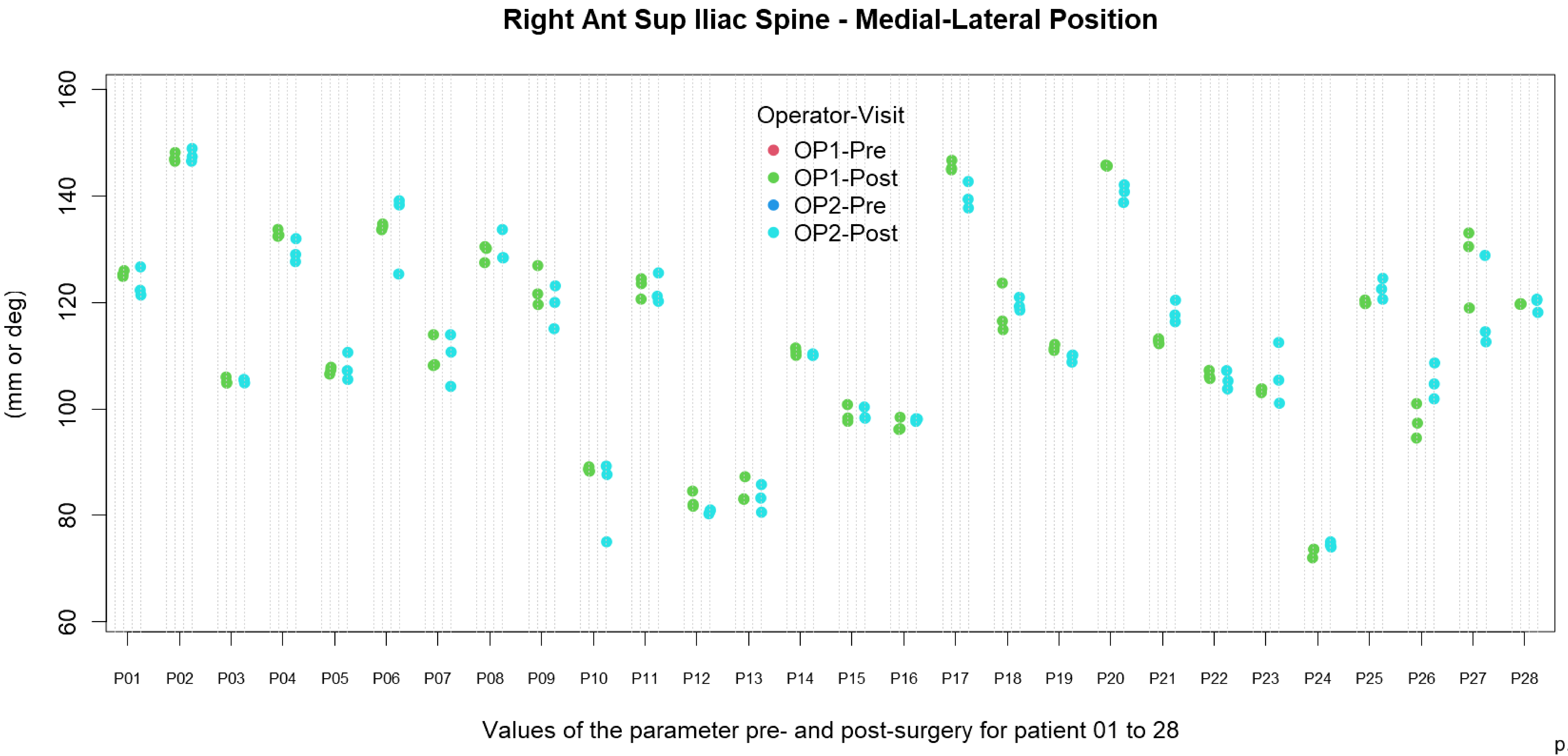

Right Ant Sup Iliac Spine - Medial-Lateral Position
Operator-Visit
OP1-Pre
OP1-Post
OP2-Pre
OP2-Post
(mm or deg)
60
80
100
120
140
160
P01
P02
P03
P04
P05
P06
P07
P08
P09
P10
P11
P12
P13
P14
P15
P16
P17
P18
P19
P20
P21
P22
P23
P24
P25
P26
P27
P28
Values of the parameter pre- and post-surgery for patient 01 to 28

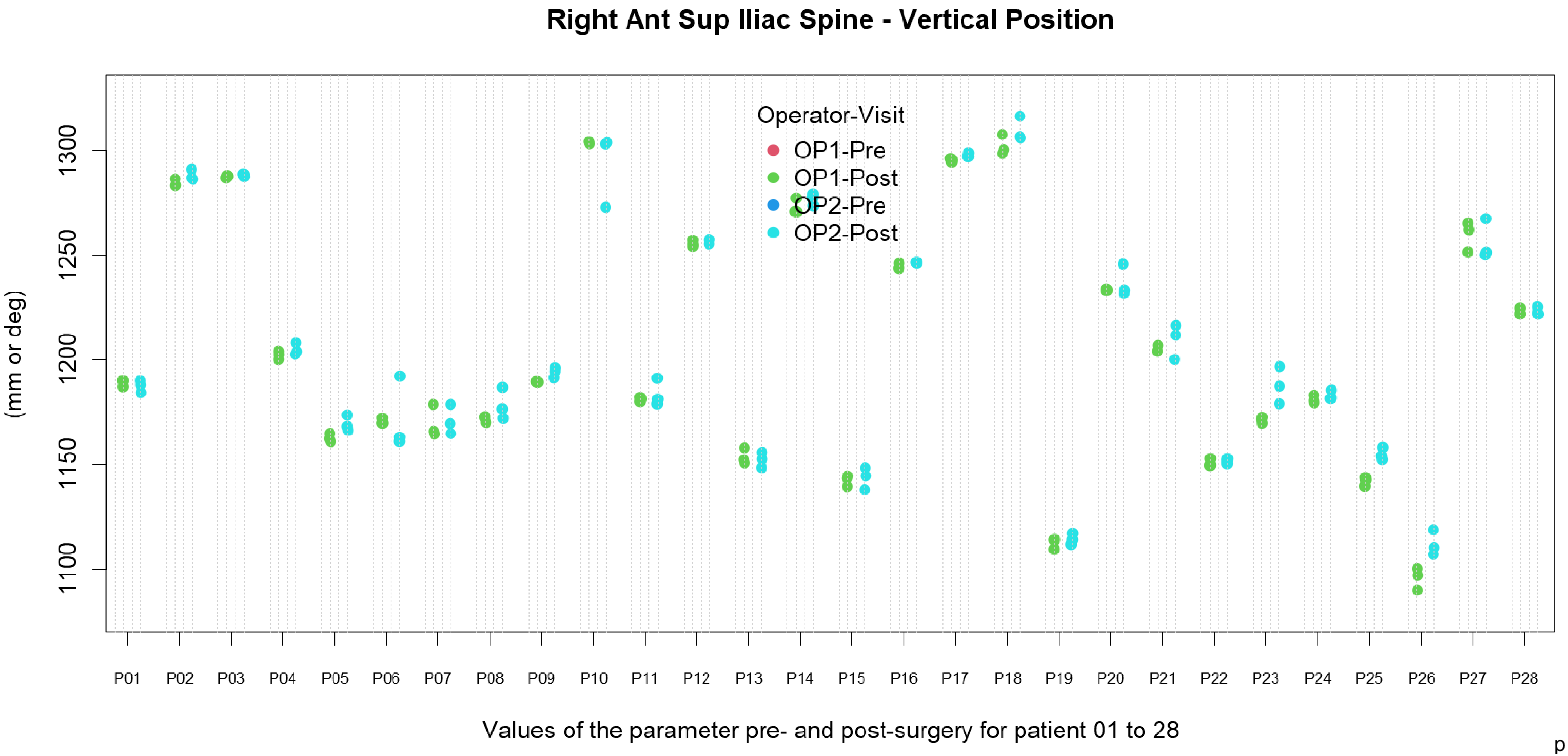
Right Ant Sup Iliac Spine - Vertical Position
Operator-Visit
OP1-Pre
OP1-Post
OP2-Pre
OP2-Post
(mm or deg)
1100
1150
1200
1250
1300
P01
P02
P03
P04
P05
P06
P07
P08
P09
P10
P11
P12
P13
P14
P15
P16
P17
P18
P19
P20
P21
P22
P23
P24
P25
P26
P27
P28
Values of the parameter pre- and post-surgery for patient 01 to 28
