# Supplementary material for: Reliability of the pelvis and femur anatomical landmarks and geometry with the EOS system before and after total hip arthroplasty
Source: Sci Rep. 2022 Dec 11;12:21420. doi: 10.1038/s41598-022-25997-3 (PMC9742167; doi:10.1038/s41598-022-25997-3)
Supplement: Supplementary file 5 — Supplementary Information 5. [file 41598_2022_25997_MOESM5_ESM.pdf]

Test-retests features of the Femur

- **Cervico-Diaphyseal Angle Contralateral** (p.2)
- **Femoral Neck Length Contralateral** (p.3)
- **Femoral Offset Contralateral** (p.4)
- **Femoral Torsion Contralateral** (p.5)
- **Femoral Head Diameter Contralateral** (p.6)
- **Femoral Length Contralateral** (p.7)
- **Hip Knee Shaft Angle Contralateral** (p.8)
- **Mechanical Angle Contralateral** (p.9)

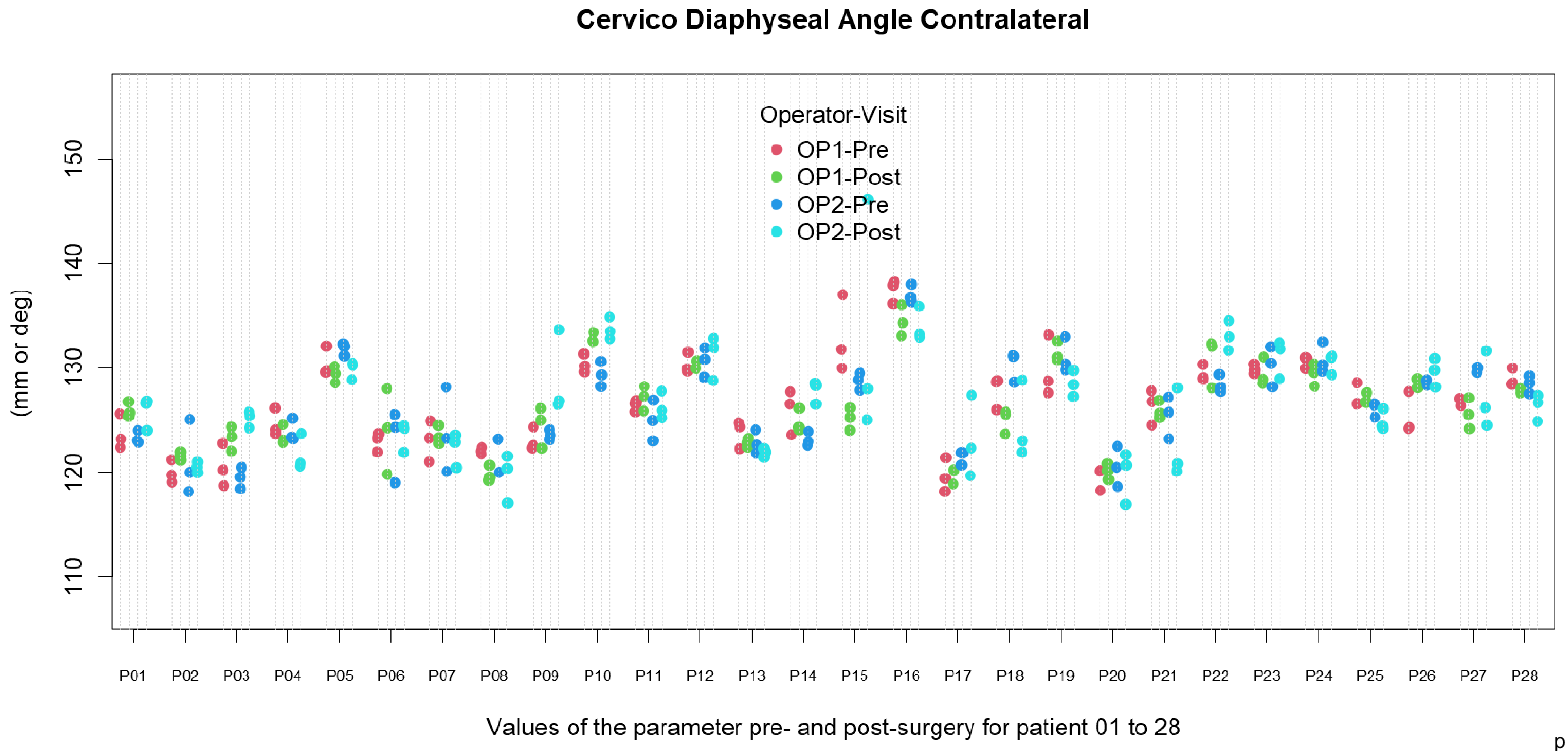
Cervico Diaphyseal Angle Contralateral
Operator-Visit
OP1-Pre
OP1-Post
OP2-Pre
OP2-Post
(mm or deg)
110
120
130
140
150
P01 P02 P03 P04 P05 P06 P07 P08 P09 P10 P11 P12 P13 P14 P15 P16 P17 P18 P19 P20 P21 P22 P23 P24 P25 P26 P27 P28
Values of the parameter pre- and post-surgery for patient 01 to 28

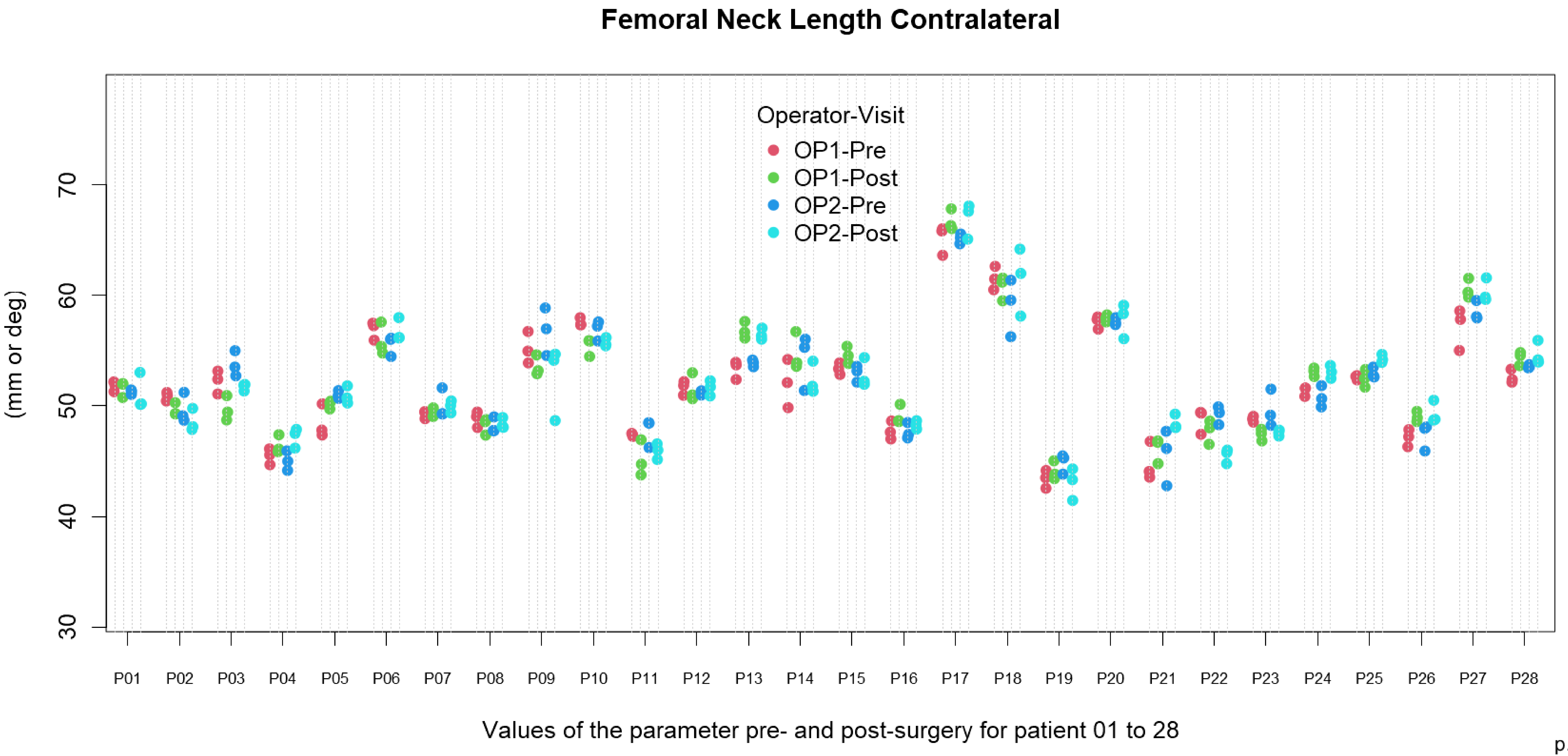
Femoral Neck Length Contralateral
Operator-Visit
OP1-Pre
OP1-Post
OP2-Pre
OP2-Post
(mm or deg)
30
40
50
60
70
P01
P02
P03
P04
P05
P06
P07
P08
P09
P10
P11
P12
P13
P14
P15
P16
P17
P18
P19
P20
P21
P22
P23
P24
P25
P26
P27
P28
Values of the parameter pre- and post-surgery for patient 01 to 28

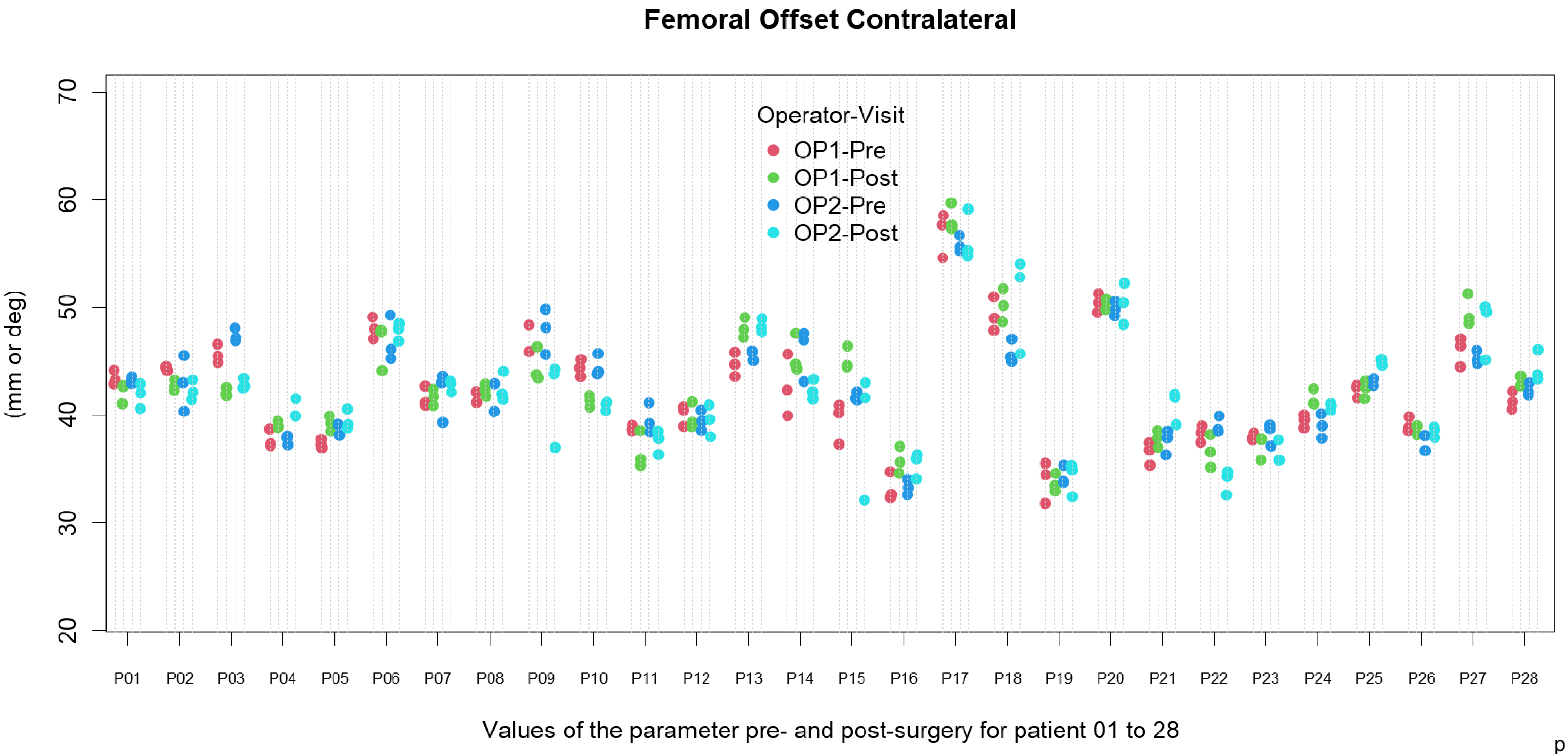

Femoral Offset Contralateral
Operator-Visit
OP1-Pre
OP1-Post
OP2-Pre
OP2-Post
(mm or deg)
20
30
40
50
60
70
P01 P02 P03 P04 P05 P06 P07 P08 P09 P10 P11 P12 P13 P14 P15 P16 P17 P18 P19 P20 P21 P22 P23 P24 P25 P26 P27 P28
Values of the parameter pre- and post-surgery for patient 01 to 28

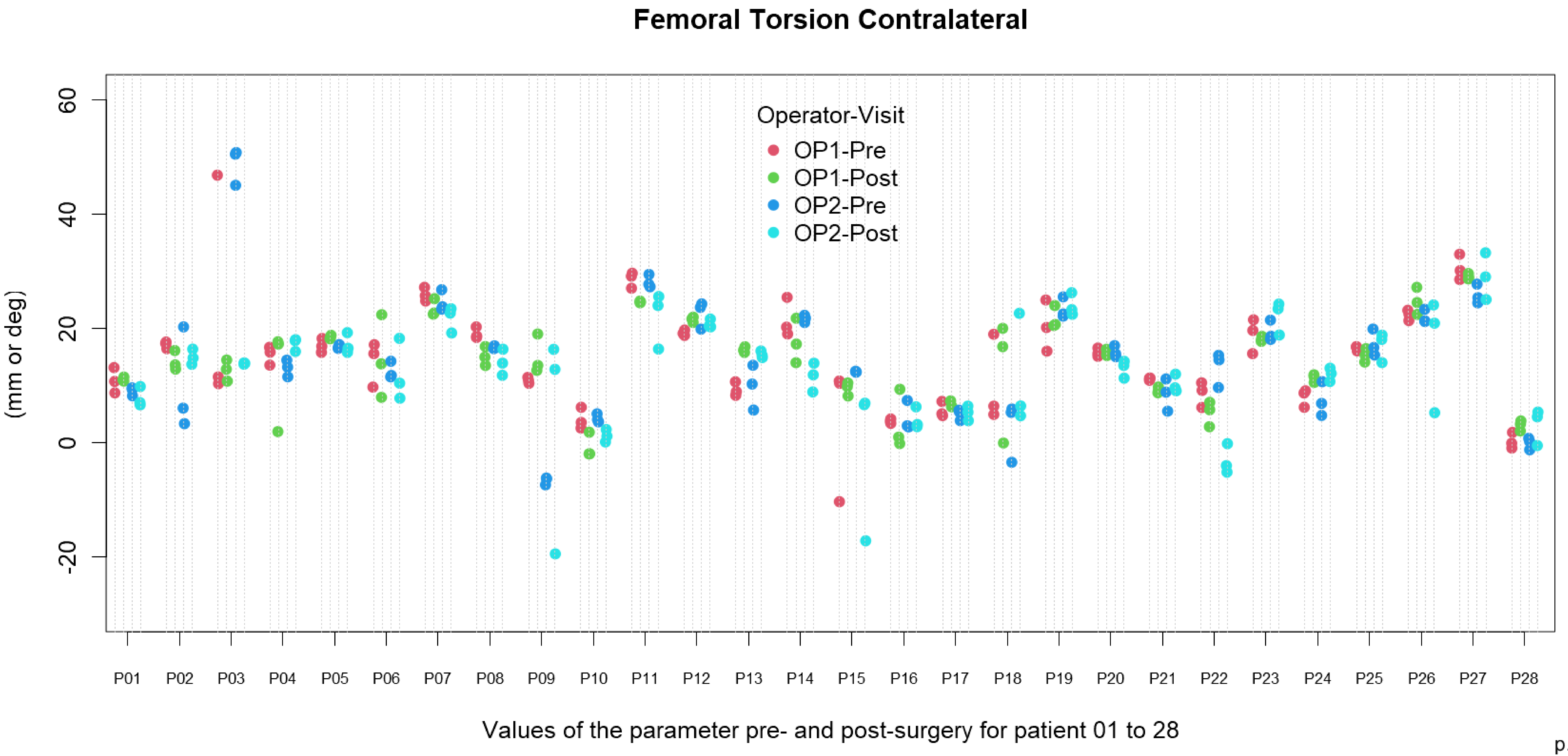
Femoral Torsion Contralateral
Operator-Visit
OP1-Pre
OP1-Post
OP2-Pre
OP2-Post
(mm or deg)
60
40
20
0
-20
P01 P02 P03 P04 P05 P06 P07 P08 P09 P10 P11 P12 P13 P14 P15 P16 P17 P18 P19 P20 P21 P22 P23 P24 P25 P26 P27 P28
Values of the parameter pre- and post-surgery for patient 01 to 28

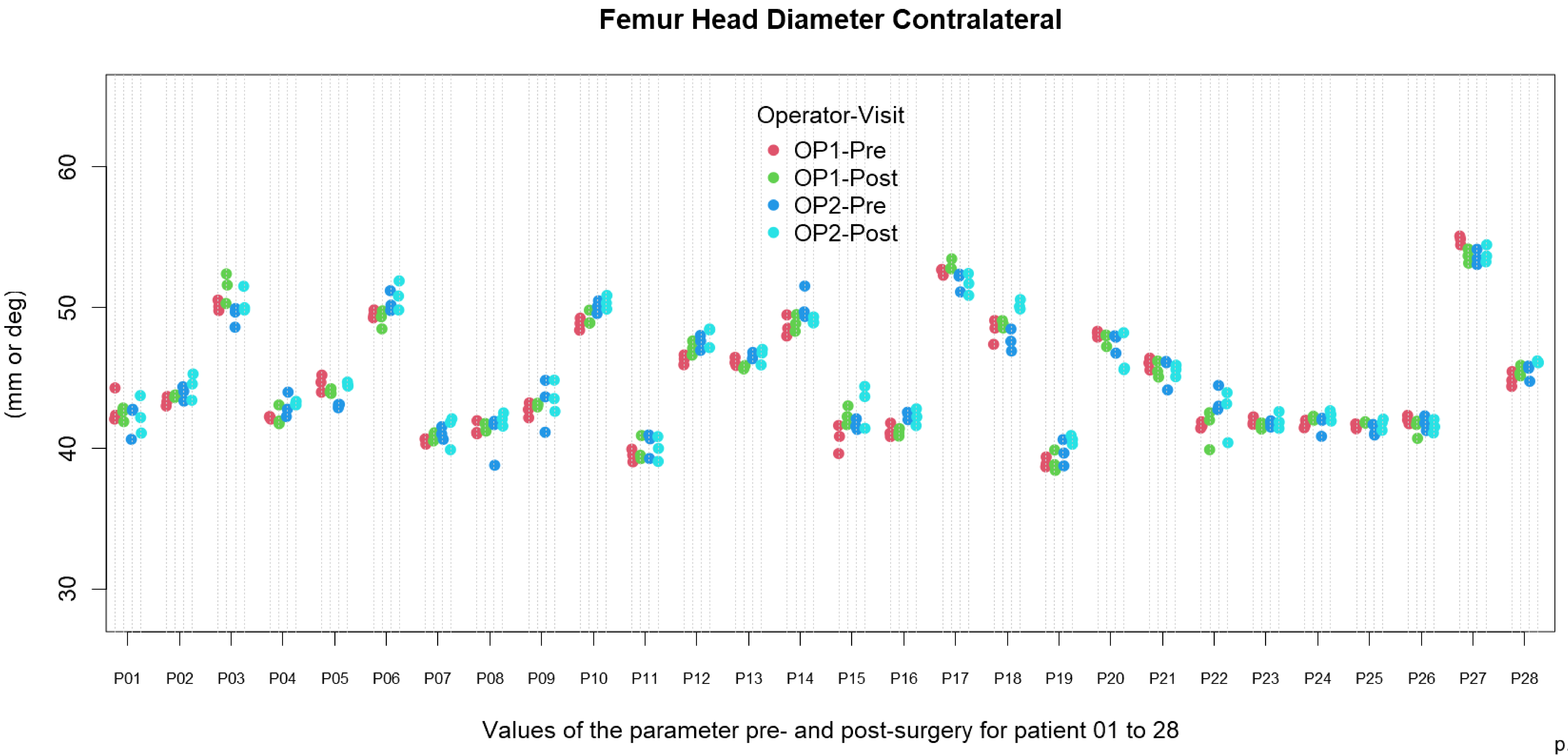

Femur Head Diameter Contralateral
Operator-Visit
OP1-Pre
OP1-Post
OP2-Pre
OP2-Post
(mm or deg)
30
40
50
60
P01
P02
P03
P04
P05
P06
P07
P08
P09
P10
P11
P12
P13
P14
P15
P16
P17
P18
P19
P20
P21
P22
P23
P24
P25
P26
P27
P28
Values of the parameter pre- and post-surgery for patient 01 to 28

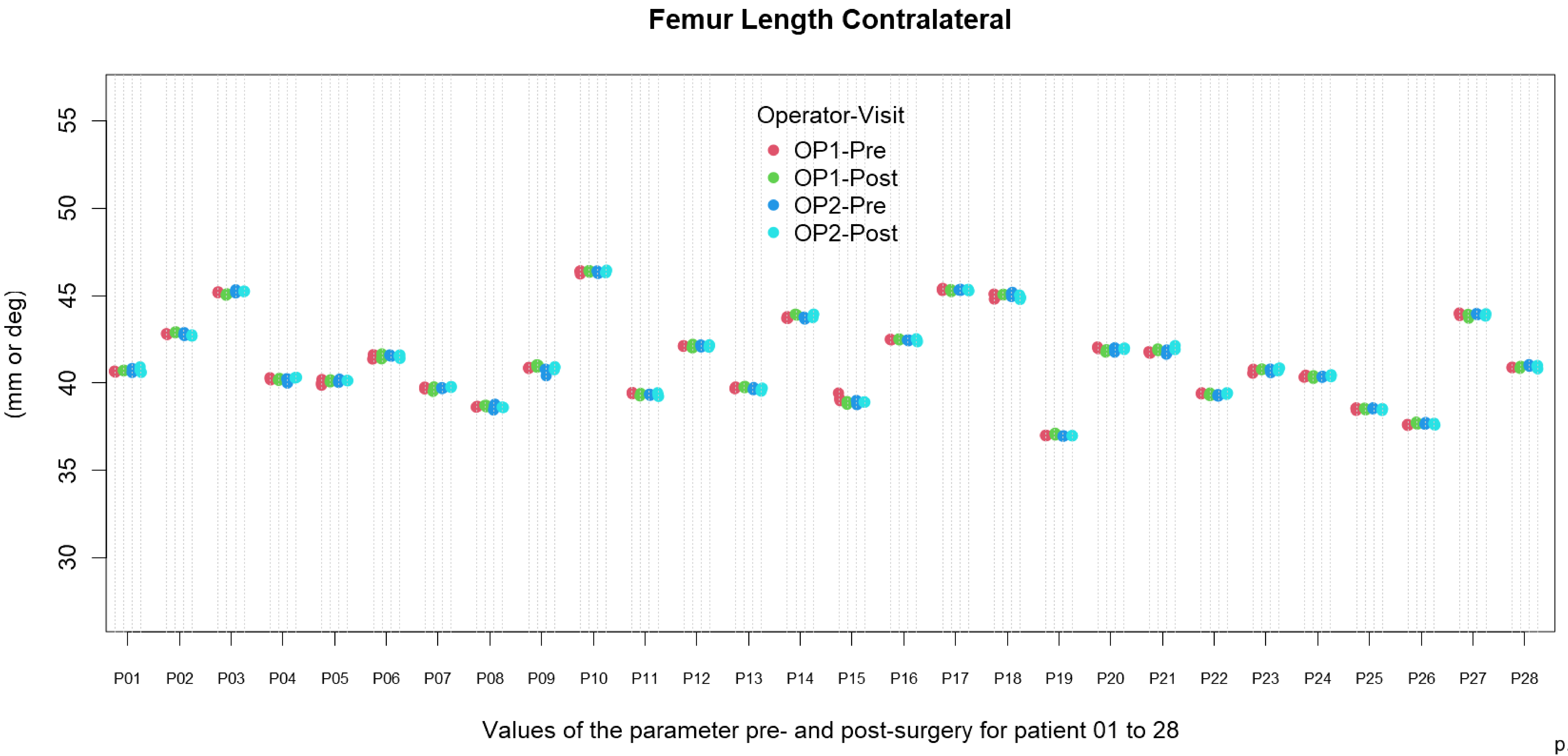
Femur Length Contralateral
Operator-Visit
OP1-Pre
OP1-Post
OP2-Pre
OP2-Post
(mm or deg)
30
35
40
45
50
55
P01
P02
P03
P04
P05
P06
P07
P08
P09
P10
P11
P12
P13
P14
P15
P16
P17
P18
P19
P20
P21
P22
P23
P24
P25
P26
P27
P28
Values of the parameter pre- and post-surgery for patient 01 to 28

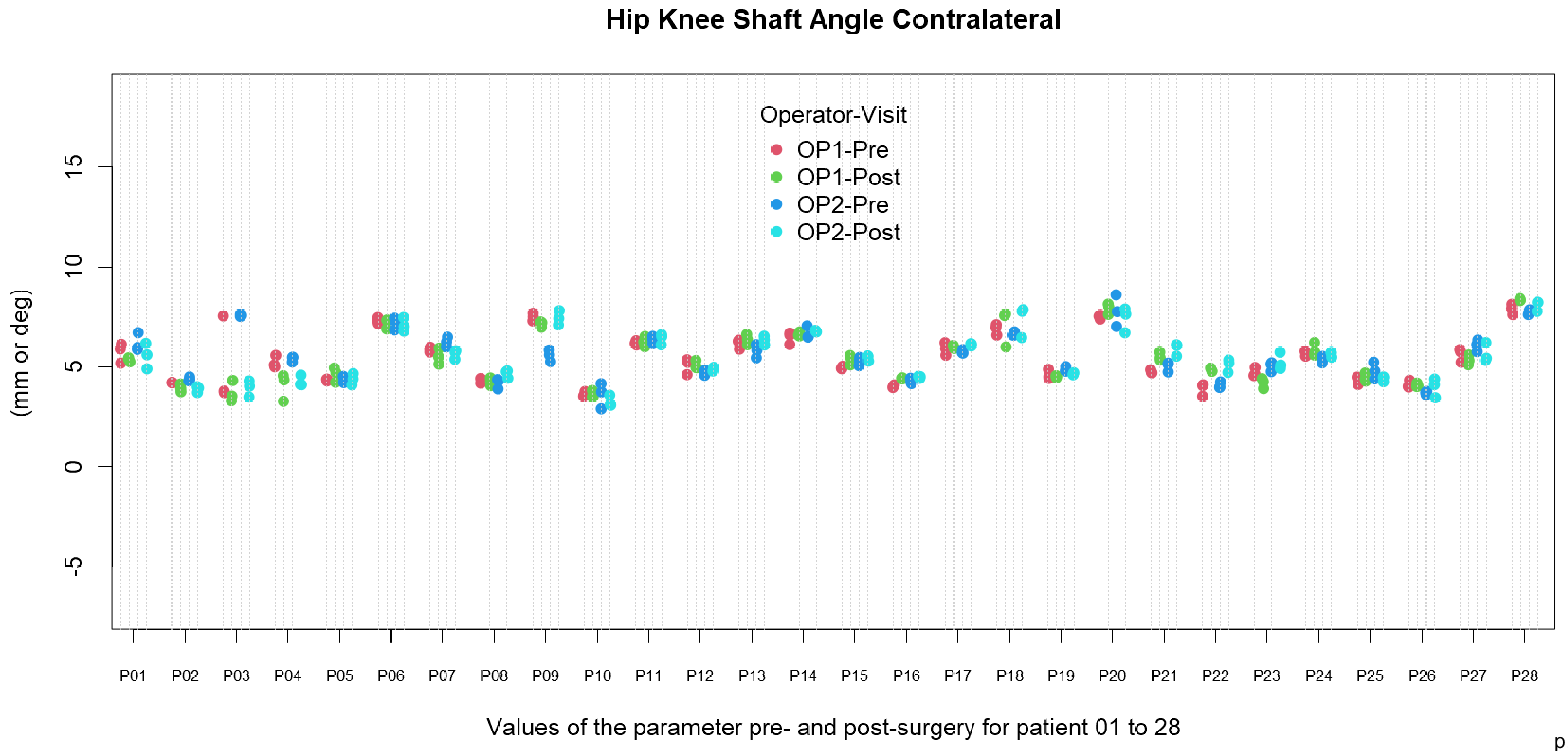

Hip Knee Shaft Angle Contralateral
Operator-Visit
OP1-Pre
OP1-Post
OP2-Pre
OP2-Post
(mm or deg)
-5
0
5
10
15
P01
P02
P03
P04
P05
P06
P07
P08
P09
P10
P11
P12
P13
P14
P15
P16
P17
P18
P19
P20
P21
P22
P23
P24
P25
P26
P27
P28
Values of the parameter pre- and post-surgery for patient 01 to 28

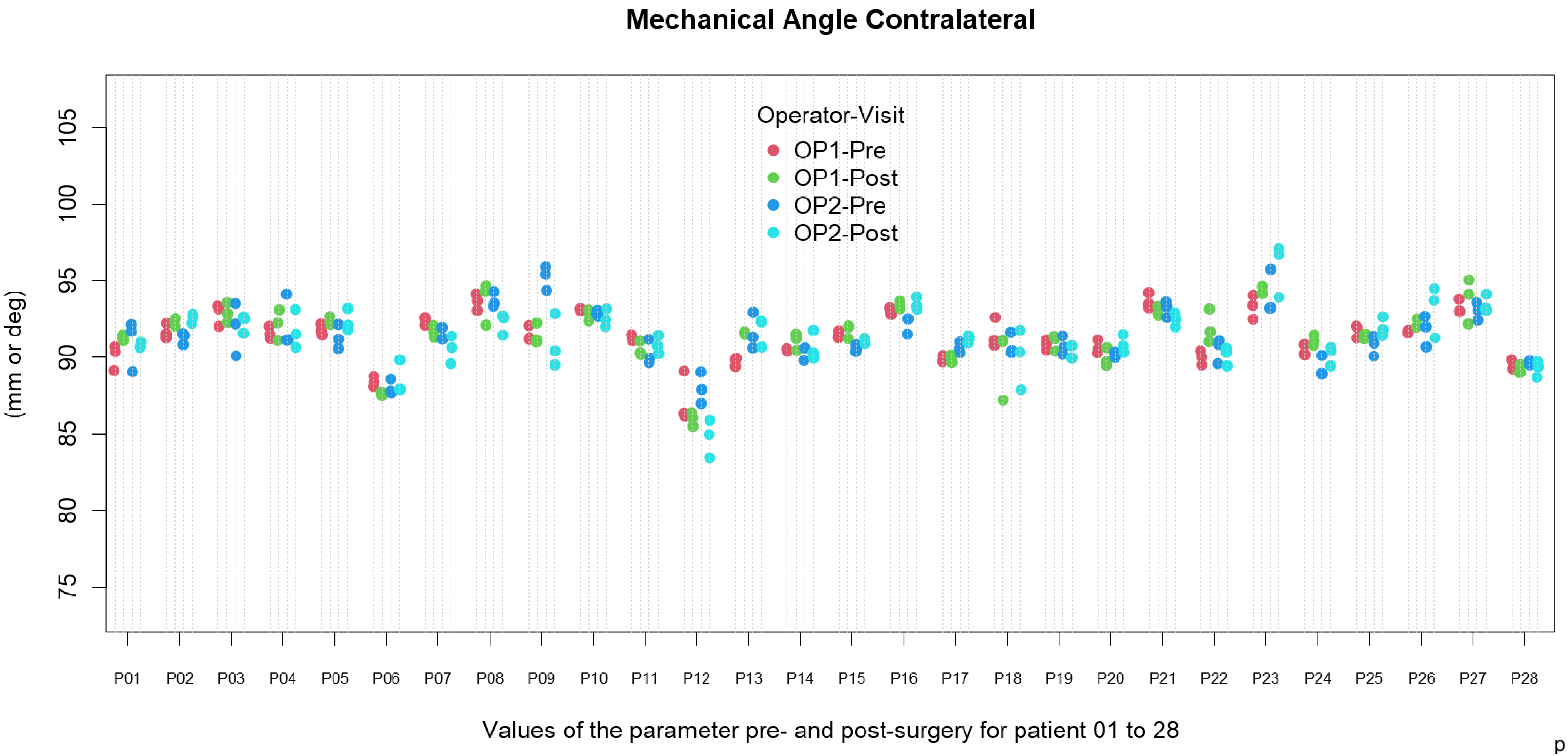

Mechanical Angle Contralateral
Operator-Visit
OP1-Pre
OP1-Post
OP2-Pre
OP2-Post
(mm or deg)
75
80
85
90
95
100
105
P01
P02
P03
P04
P05
P06
P07
P08
P09
P10
P11
P12
P13
P14
P15
P16
P17
P18
P19
P20
P21
P22
P23
P24
P25
P26
P27
P28
Values of the parameter pre- and post-surgery for patient 01 to 28
