# Supplementary material for: Reliability of the pelvis and femur anatomical landmarks and geometry with the EOS system before and after total hip arthroplasty
Source: Sci Rep. 2022 Dec 11;12:21420. doi: 10.1038/s41598-022-25997-3 (PMC9742167; doi:10.1038/s41598-022-25997-3)
Supplement: Supplementary file 6 — Supplementary Information 6. [file 41598_2022_25997_MOESM6_ESM.pdf]

Pelvis features dependent of posture and/or surgery

- **APP Inclination** (p.2)
- **Axial Rotation Pelvis** (p.3)
- **Obliquity Pelvis** (p.4)
- **Pelvic Incidence** (p.5)
- **Pelvic Version** (p.6)
- **Sacral Slope** (p.7)

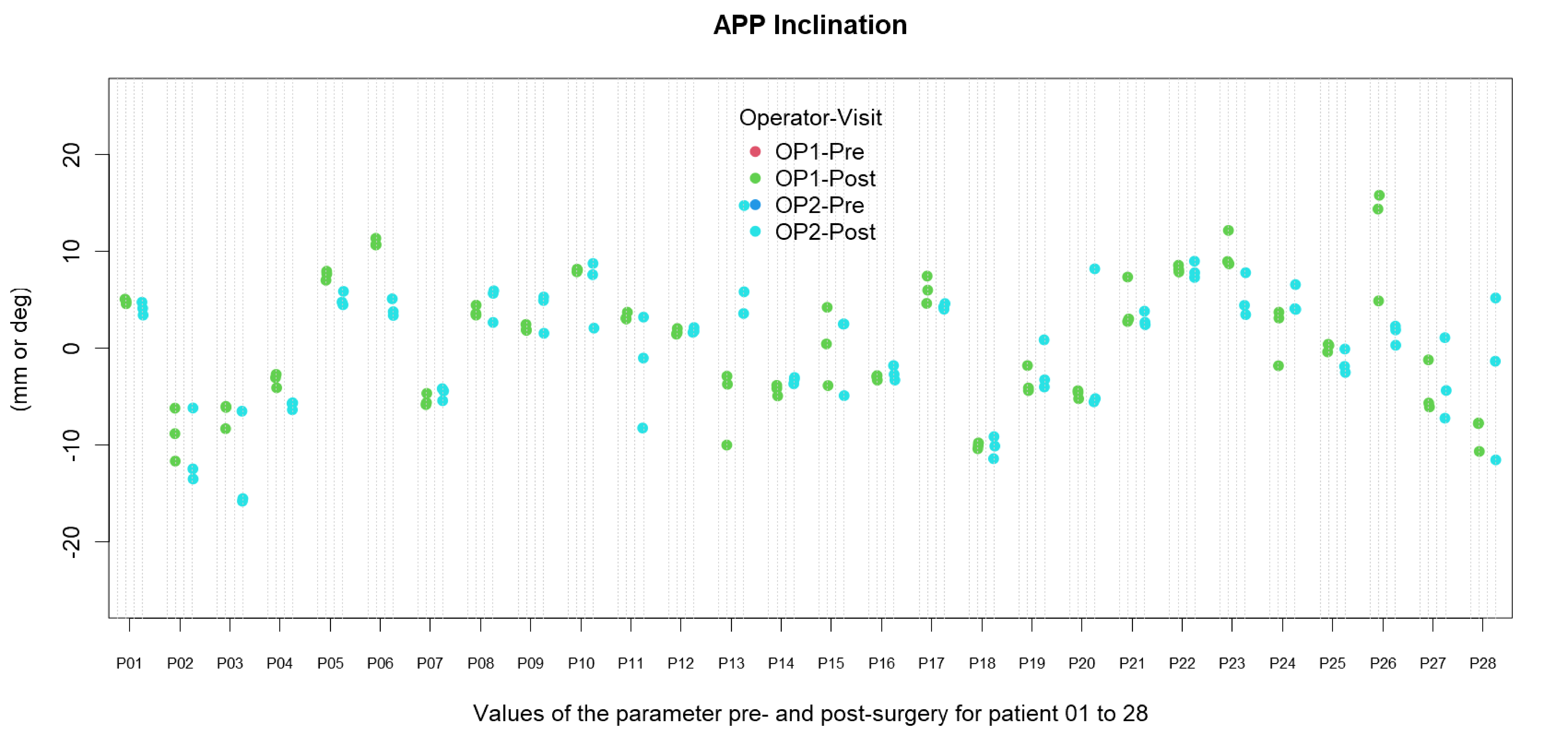
APP Inclination
Operator-Visit
OP1-Pre
OP1-Post
OP2-Pre
OP2-Post
(mm or deg)
20
10
0
-10
-20
P01
P02
P03
P04
P05
P06
P07
P08
P09
P10
P11
P12
P13
P14
P15
P16
P17
P18
P19
P20
P21
P22
P23
P24
P25
P26
P27
P28
Values of the parameter pre- and post-surgery for patient 01 to 28

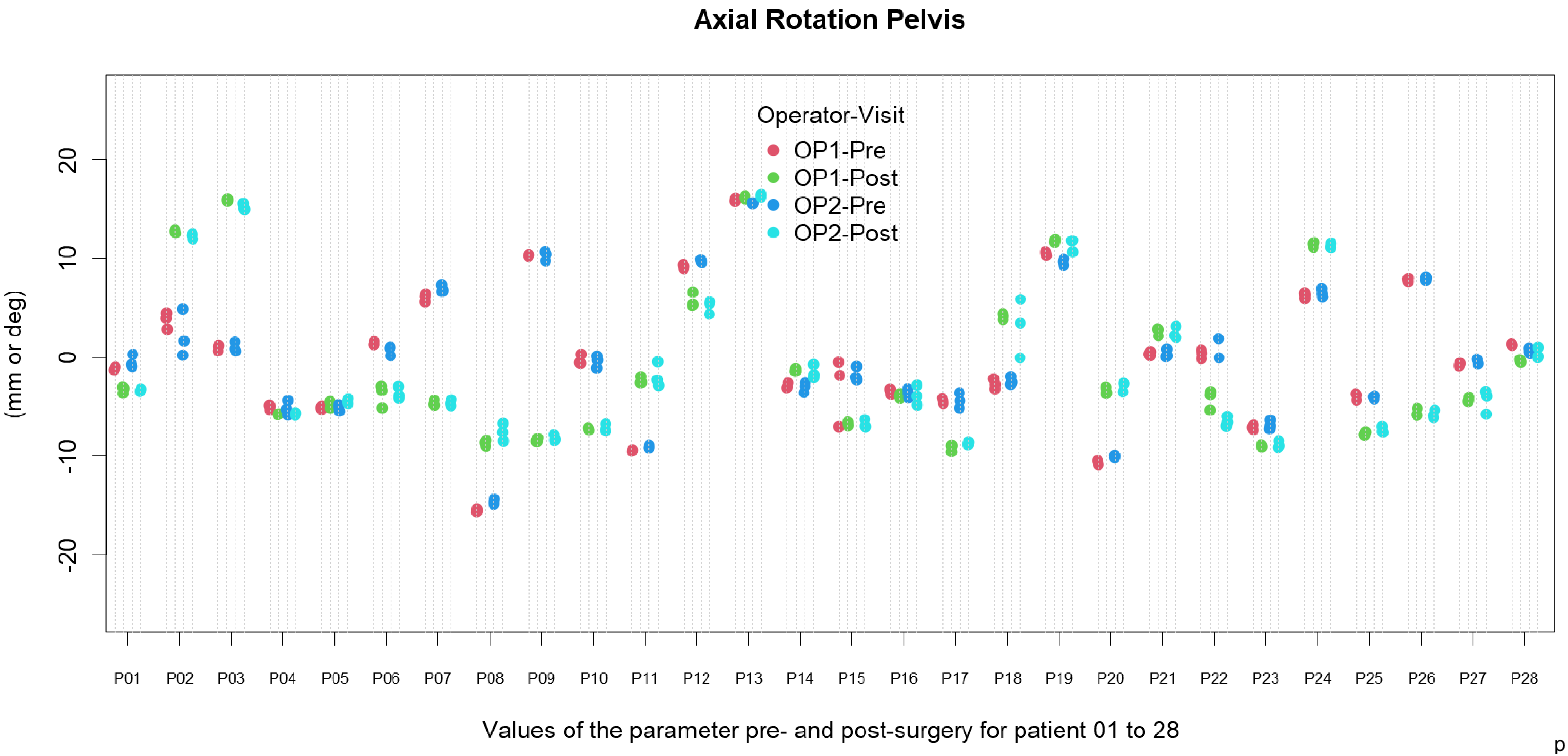

Axial Rotation Pelvis
Operator-Visit
OP1-Pre
OP1-Post
OP2-Pre
OP2-Post
(mm or deg)
20
10
0
-10
-20
P01
P02
P03
P04
P05
P06
P07
P08
P09
P10
P11
P12
P13
P14
P15
P16
P17
P18
P19
P20
P21
P22
P23
P24
P25
P26
P27
P28
Values of the parameter pre- and post-surgery for patient 01 to 28

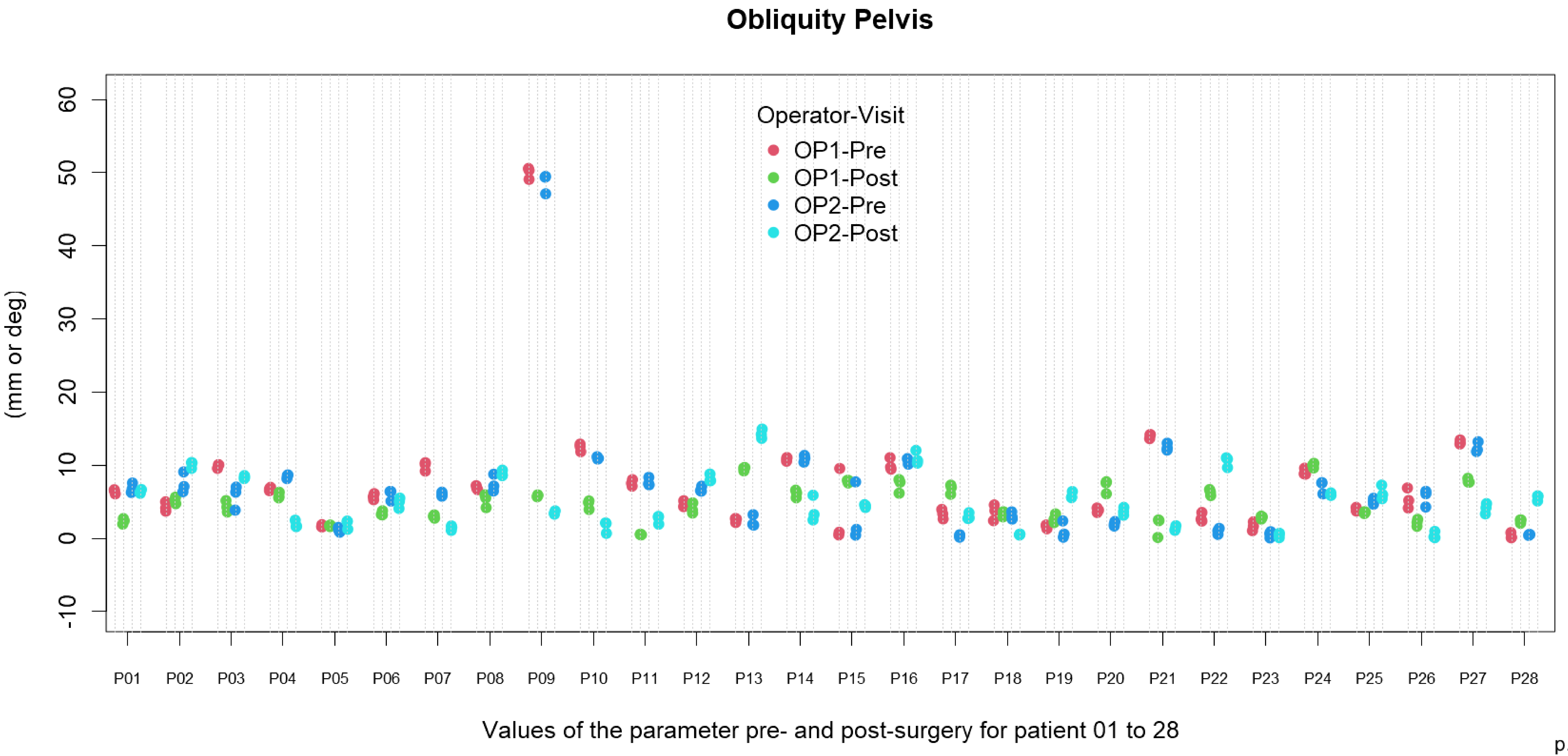

Obliquity Pelvis
Operator-Visit
OP1-Pre
OP1-Post
OP2-Pre
OP2-Post
(mm or deg)
60
50
40
30
20
10
0
-10
P01
P02
P03
P04
P05
P06
P07
P08
P09
P10
P11
P12
P13
P14
P15
P16
P17
P18
P19
P20
P21
P22
P23
P24
P25
P26
P27
P28
Values of the parameter pre- and post-surgery for patient 01 to 28

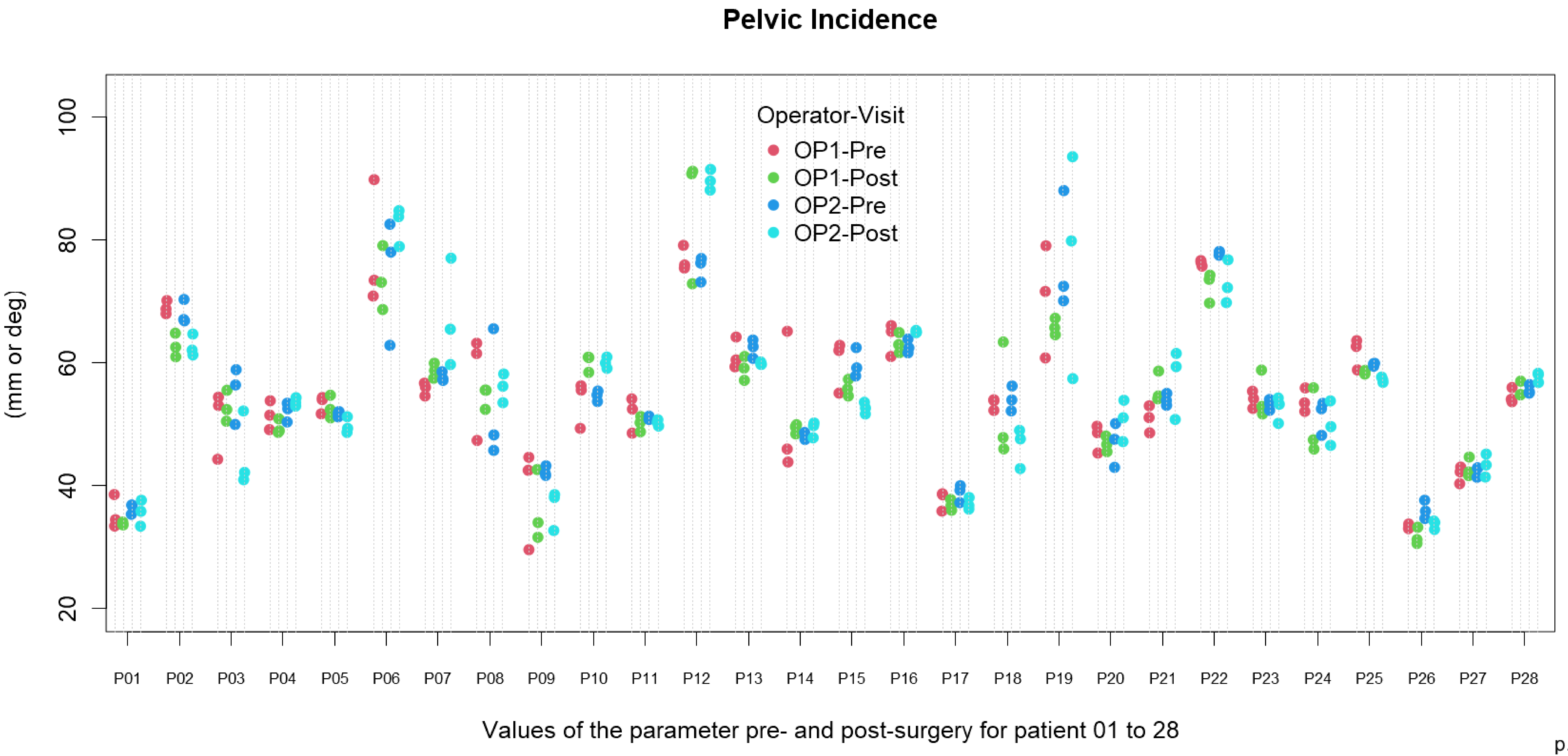

Pelvic Incidence
Operator-Visit
OP1-Pre
OP1-Post
OP2-Pre
OP2-Post
(mm or deg)
20
40
60
80
100
P01
P02
P03
P04
P05
P06
P07
P08
P09
P10
P11
P12
P13
P14
P15
P16
P17
P18
P19
P20
P21
P22
P23
P24
P25
P26
P27
P28
Values of the parameter pre- and post-surgery for patient 01 to 28

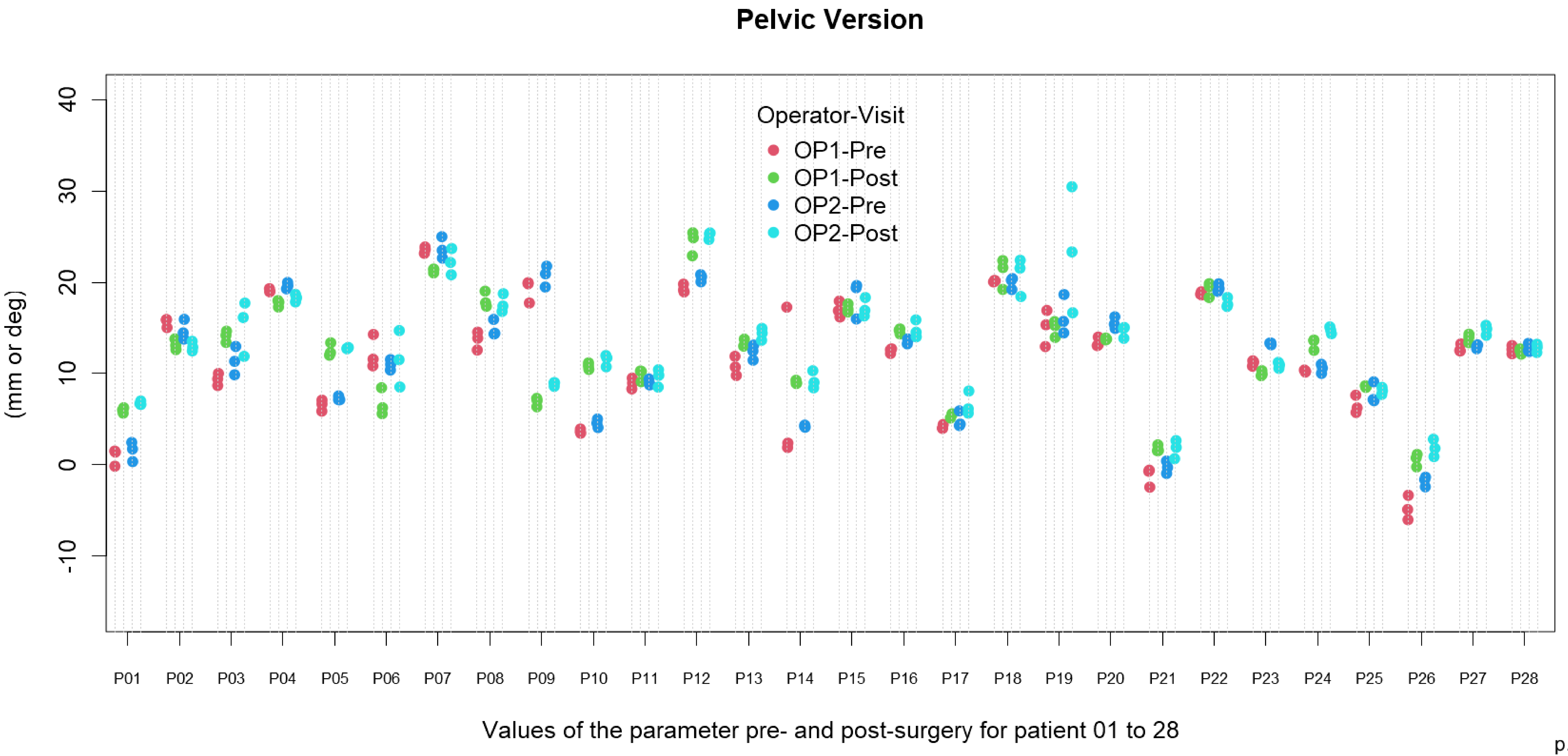

Pelvic Version
Operator-Visit
OP1-Pre
OP1-Post
OP2-Pre
OP2-Post
(mm or deg)
-10
0
10
20
30
40
P01
P02
P03
P04
P05
P06
P07
P08
P09
P10
P11
P12
P13
P14
P15
P16
P17
P18
P19
P20
P21
P22
P23
P24
P25
P26
P27
P28
Values of the parameter pre- and post-surgery for patient 01 to 28

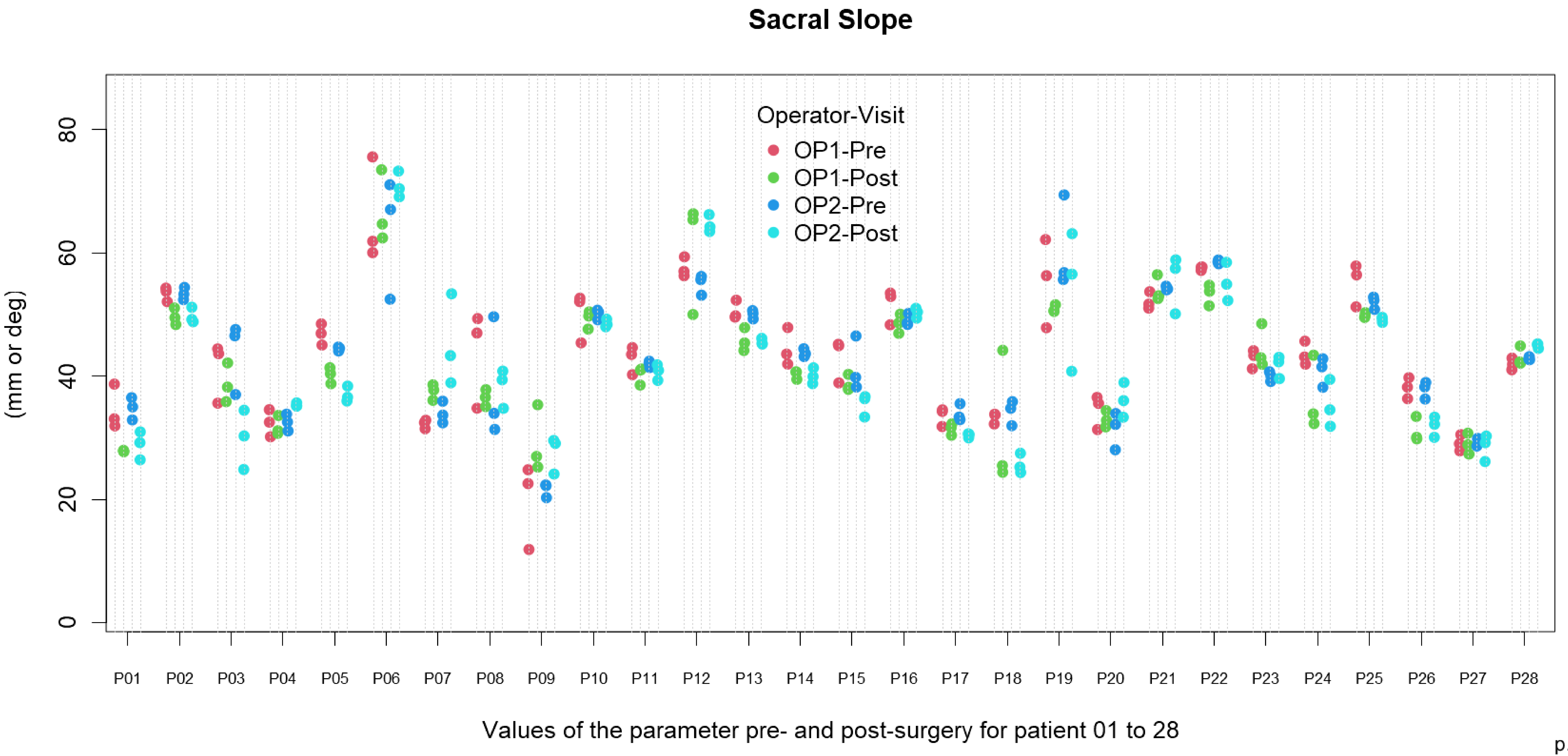

Sacral Slope
Operator-Visit
OP1-Pre
OP1-Post
OP2-Pre
OP2-Post
(mm or deg)
0
20
40
60
80
P01
P02
P03
P04
P05
P06
P07
P08
P09
P10
P11
P12
P13
P14
P15
P16
P17
P18
P19
P20
P21
P22
P23
P24
P25
P26
P27
P28
Values of the parameter pre- and post-surgery for patient 01 to 28
