# Supplementary material for: Reliability of the pelvis and femur anatomical landmarks and geometry with the EOS system before and after total hip arthroplasty
Source: Sci Rep. 2022 Dec 11;12:21420. doi: 10.1038/s41598-022-25997-3 (PMC9742167; doi:10.1038/s41598-022-25997-3)
Supplement: Supplementary file 9 — Supplementary Information 9. [file 41598_2022_25997_MOESM9_ESM.pdf]

Table 1: Intra and inter-operator smallest detectable change (SDC) of the position of anatomical points (in mm). *AP*: anterior-posterior direction, *ML*: medio-lateral direction, *V*: vertical direction, *R*: radius, *Ant*: anterior, *Sup*: superior, *Homo*: homolateral side, *Contra*: contralateral side, *Acet*: acetabulum, *Fem*: femoral, *THA*: Total Hip Arthroplasty, *Gr. Troch*: Greater Trochanter.

	**Intra-operator**				**Inter-operator**			
**Pelvis**	AP	ML	V	R	AP	ML	V	R
Pubic Symphysis	5.0	1.8	3.2	-	5.5	1.8	3.4	-
Centre Sacral Slope	6.3	5.9	4.9	-	7.1	5.9	5.0	-
Left Ant. Sup. Iliac Spine	15.3	6.2	9.5	-	21.5	9.6	11.8	-
Right Ant. Sup. Iliac Spine	17.2	8.3	14.4	-	19.1	8.9	16.0	-
Homo. Acetabulum	3.6	3.2	3.0	2.4	3.7	4.9	2.8	5.4
Contra. Acetabulum	4.8	2.5	2.6	2.3	4.8	3.4	4.2	4.6
Contra. Acet. pre-THA	4.1	4.1	2.3	2.0	4.8	3.2	3.6	5.0
Contra. Acet. post-THA	5.0	2.7	2.6	2.4	5.1	3.5	4.8	4.1
**Femur**	AP	ML	V	R	AP	ML	V	R
Homo. Fem. Head pre-THA	4.7	1.9	2.5	1.9	4.5	2.5	2.8	2.3
Contra. Fem. Head	4.6	1.2	1.5	1.4	4.7	1.4	1.7	1.5
Contra. Fem. Head pre-THA	4.6	1.2	1.4	1.4	4.7	1.4	1.6	1.4
Contra. Fem. Head post-THA	4.6	1.2	1.5	1.4	4.8	1.6	1.8	1.6
Left Gr. Troch. pre-THA	6.0	4.4	2.6	-	5.2	5.0	2.9	-
Right Gr. Troch. pre-THA	5.2	4.5	2.8	-	6.0	4.5	2.9	-
Contra. Gr. Troch. post-THA	15.1	4.2	2.6	-	16.9	4.4	2.4	-
Contra. Lateral Condyle	4.1	3.3	1.2	1.8	4.7	3.7	1.5	2.0
Contra. Medial Condyle	4.2	2.9	1.3	1.9	5.0	4.2	1.5	2.6
Homo. Lateral Condyle	4.5	4.0	1.3	1.8	5.3	4.3	1.4	1.9
Homo. Medial Condyle	5.5	3.0	1.3	1.7	5.9	3.5	1.3	2.4
**Implant**	AP	ML	V	R	AP	ML	V	R
Acetabular Cup	1.4	1.5	1.5	1.2	1.7	1.6	1.5	1.7
Stem Femoral Head	0.8	0.8	0.8	0.7	0.7	0.8	0.9	0.7

**Legend**			
Excellent (<3mm)	Good (3 to 5 mm)	Moderate (5 to 10mm)	Poor (>10mm)

Table 2: Intra and inter-operator intra-class correlation (ICC) of the position of anatomical points. *AP*: anterior-posterior direction, *ML*: medio-lateral direction, *V*: vertical direction, *R*: radius, *Ant*: anterior, *Sup*: superior, *Homo*: homolateral side, *Contra*: contralateral side, *Acet*: acetabulum, *Fem*: femoral, *THA*: Total Hip Arthroplasty, *Gr. Troch*: Greater Trochanter.

	**Intra-operator**				**Inter-operator**			
**Pelvis**	AP	ML	V	R	AP	ML	V	R
Pubic Symphysis	0.997	0.994	1.000	-	0.997	0.994	1.000	-
Centre Sacral Slope	0.996	0.953	0.999	-	0.995	0.953	0.999	-
Left Ant. Sup. Iliac Spine	0.979	0.976	0.997	-	0.959	0.940	0.995	-
Right Ant. Sup. Iliac Spine	0.973	0.975	0.992	-	0.967	0.972	0.990	-
Homo. Acetabulum	0.999	0.996	1.000	0.879	0.998	0.990	1.000	0.354
Contra. Acetabulum	0.998	0.995	1.000	0.860	0.998	0.991	0.999	0.468
Contra. Acet. pre-THA	0.998	0.998	1.000	0.900	0.997	0.993	0.999	0.365
Contra. Acet. post-THA	0.997	0.993	1.000	0.841	0.997	0.988	0.999	0.556
**Femur**	AP	ML	V	R	AP	ML	V	R
Homo. Fem. Head pre-THA	0.998	0.998	1.000	0.909	0.998	0.997	1.000	0.872
Contra. Fem. Head	0.998	0.999	1.000	0.937	0.998	0.998	1.000	0.929
Contra. Fem. Head Pre-THA	0.998	0.999	1.000	0.942	0.998	0.999	1.000	0.937
Contra. Fem. Head Post-THA	0.998	0.999	1.000	0.939	0.998	0.998	1.000	0.913
Left Gr. Troch. pre-THA	0.997	0.994	1.000	-	0.998	0.992	1.000	-
Right Gr. Troch. pre-THA	0.997	0.990	1.000	-	0.995	0.990	1.000	-
Contra. Gr. Troch. post-THA	0.979	0.986	1.000	-	0.974	0.984	1.000	-
Contra. Lateral Condyle	0.998	0.995	1.000	0.861	0.997	0.993	1.000	0.826
Contra. Medial Condyle	0.998	0.996	1.000	0.873	0.997	0.992	1.000	0.743
Homo. Lateral Condyle	0.998	0.994	1.000	0.830	0.997	0.992	1.000	0.810
Homo. Medial Condyle	0.997	0.996	1.000	0.879	0.997	0.995	1.000	0.755
**Implant**	AP	ML	V	R	AP	ML	V	R
Acetabular Cup	1.000	0.998	1.000	0.929	1.000	0.998	1.000	0.851
Stem Femoral Head	1.000	1.000	1.000	0.955	1.000	1.000	1.000	0.950

**Legend**			
Excellent (>0.9)	Good (0.75 to 0.9)	Moderate (0.5 to 0.75)	Poor (<0.5)

Table 4: Intra-operator (Intra), intra-operator (Inter) and test-retest (TRtest) ICC and SDC of pelvis and femur geometrical parameters independent of posture and surgery. *Symph*: Symphysis, *Contra*: Contralateral, *Acet*: Acetabulum.

	**ICC**			**SDC**		
**Pelvis**	**Intra**	**Inter**	**TRtest**	**Intra**	**Inter**	**TRtest**
Diameter Acetabulum Contralateral (mm)	0.860	0.468	0.843	2.3	4.6	2.5
Distance Pubic Symph. to Contra. Acet. (mm)	0.946	0.941	0.887	4.1	4.3	5.9
Distance Centre Sacral Slope to Contra. Acet. (mm)	0.932	0.894	0.916	6.4	8.0	7.1
Distance Centre Sacral Slope to Pubic Symp. (mm)	0.966	0.957	0.949	5.5	6.2	6.7
**Femur**	**Intra**	**Inter**	**TRtest**	**Intra**	**Inter**	**TRtest**
Femur Length Contralateral (mm)	0.999	0.999	0.998	2.4	2.5	2.6
Femur Head Diameter Contralateral (mm)	0.969	0.958	0.965	2.0	2.3	2.1
Femoral Neck Length Contralateral (mm)	0.950	0.947	0.911	3.2	3.3	4.3
Femoral Offset Contralateral (mm)	0.916	0.915	0.853	4.3	4.3	5.7
Hip Knee Shaft Angle Contralateral. (°)	0.904	0.879	0.801	1.1	1.2	1.6
Cervico Diaphyseal Angle Contralateral (°)	0.784	0.797	0.734	5.8	5.7	6.5
Mechanical Angle Contralateral (°)	0.804	0.779	0.727	2.3	2.4	2.7
Femoral Torsion Contralateral (°)	0.744	0.696	0.618	13.1	14.2	15.9

Legend

Excellent	Good	Moderate	Poor

Table 5: Intra-operator (Intra) and inter-operator (Inter) ICC and SDC of pelvis, femur and implant geometrical parameters dependent of posture and/or surgery. *APP*: anterior pelvic plane, *Homo*: Homolateral side, *Fem*: Femoral, *THA*: Total Hip Arthroplasty, *Diaph*: Diaphiseal, *Contra*: Contralateral, *w.r.t*: with respect to.

	ICC		SDC	
**Pelvis**	**Intra**	**Inter**	**Intra**	**Inter**
Axial Rotation (°)	0.992	0.991	1.9	1.9
Obliquity (°)	0.968	0.928	3.4	5.1
Sacral Slope (°)	0.896	0.904	9.8	9.4
Pelvic Version (°)	0.950	0.936	4.0	4.5
Pelvic Incidence (°)	0.894	0.900	11.5	11.2
APP Inclination (°)	0.809	0.658	7.7	10.4
**Femur**	**Intra**	**Inter**	**Intra**	**Inter**
Femur Length Homolateral (mm)	0.998	0.998	2.7	2.8
Femoral Offset Homo. Pre-THA (mm)	0.926	0.912	4.1	4.5
Femoral Offset Homo. Post-THA (mm)	0.981	0.981	2.0	2.0
Fem. Neck Length Homo. Pre-THA (mm)	0.943	0.925	3.3	3.8
Hip Knee Shaft Angle Homolateral (°)	0.904	0.890	1.4	1.5
Cervico Diaph. Angle Homolateral (°)	0.836	0.826	5.7	5.9
Femoral Torsion Contra. Pre-THA (°)	0.690	0.678	13.2	13.4
Femoral Torsion Contra. Post-THA (°)	0.833	0.665	11.3	16.1
**Implant**	**Intra**	**Inter**	**Intra**	**Inter**
Cup Anteversion w.r.t APP (°)	0.860	0.774	9.9	12.5
Cup Anteversion w.r.t cabin (°)	0.873	0.852	8.2	8.9
Cup Inclination w.r.t APP (°)	0.725	0.603	6.0	7.2
Cup Inclination w.r.t cabin (°)	0.884	0.866	3.8	4.0
Antetorsion Stem (°)	0.880	0.841	10.7	12.3
**Legend**				
Excellent	Good	Moderate	Poor	
