# Supplementary material for: Reliability of the pelvis and femur anatomical landmarks and geometry with the EOS system before and after total hip arthroplasty
Source: Sci Rep. 2022 Dec 11;12:21420. doi: 10.1038/s41598-022-25997-3 (PMC9742167; doi:10.1038/s41598-022-25997-3)
Supplement: Supplementary file 10 — Supplementary Information 10. [file 41598_2022_25997_MOESM10_ESM.pdf]

Overview Poor SDC > 10 mm/°

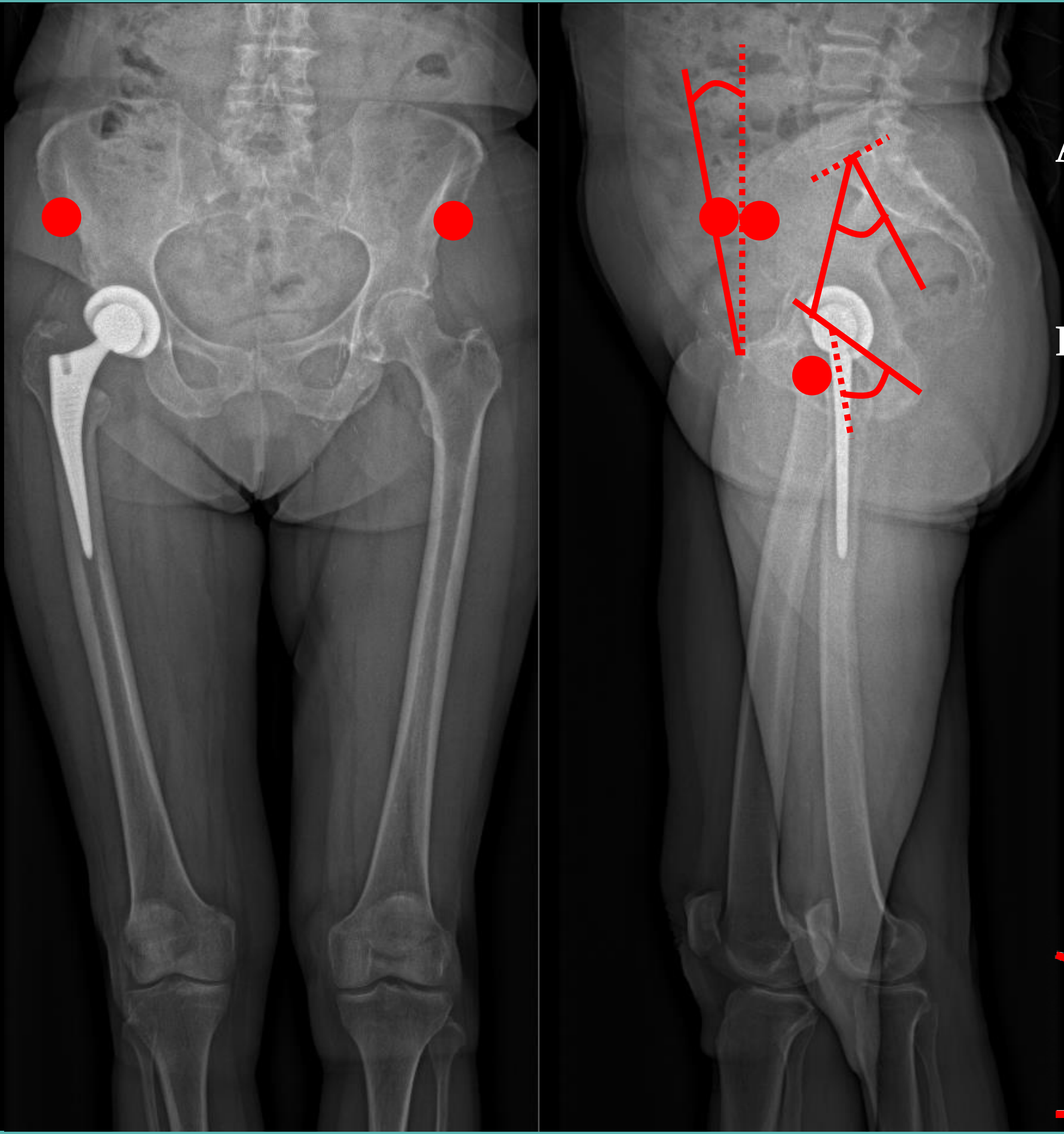

Anatomical Landmarks
- Anterior Superior Iliac Spines
- Contra Gr. Troch. post-THA (AP)

Pelvis and Femur Geom
- Pelvic incidence
- APP Inclination (*Inter*)
- Cup anteversion w.r.t APP (*Inter*)
- Femoral torsion (*Intra*, *TRtest*)
- Femoral torsion contra post THA
- Antetorsion Stem (*Inter*)

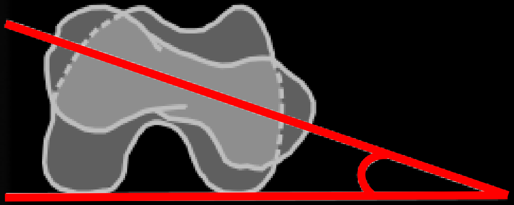

Overview Moderate SDC [5 -10 mm] – P & L

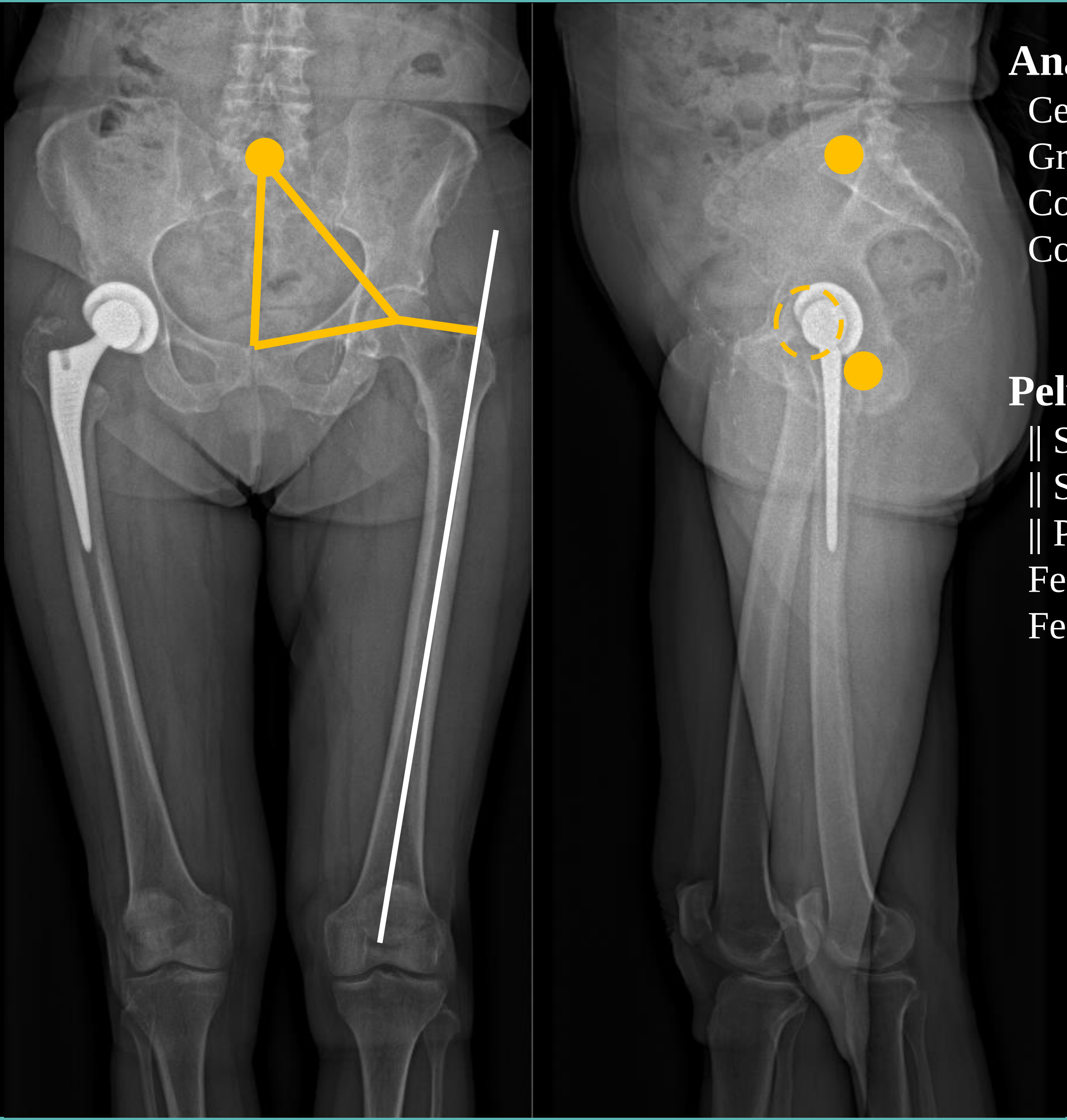

Anatomical Landmarks

Centre Sacral Slope
Greater Trochanter pre-THA - AP
Contra. Acetabulum post-THA - AP
Condyles - AP

Pelvis and Femur Geom

|| SSC – PSYM ||
|| SSC – Conta. Acet. ||
|| PSYM – Conta. Acet. || (*TRtest*)
Fem Offset Contra. (*TRtest*)
Fem Offset Contra. Pre-THA

Overview moderate SDC [5 -10°] – Angles

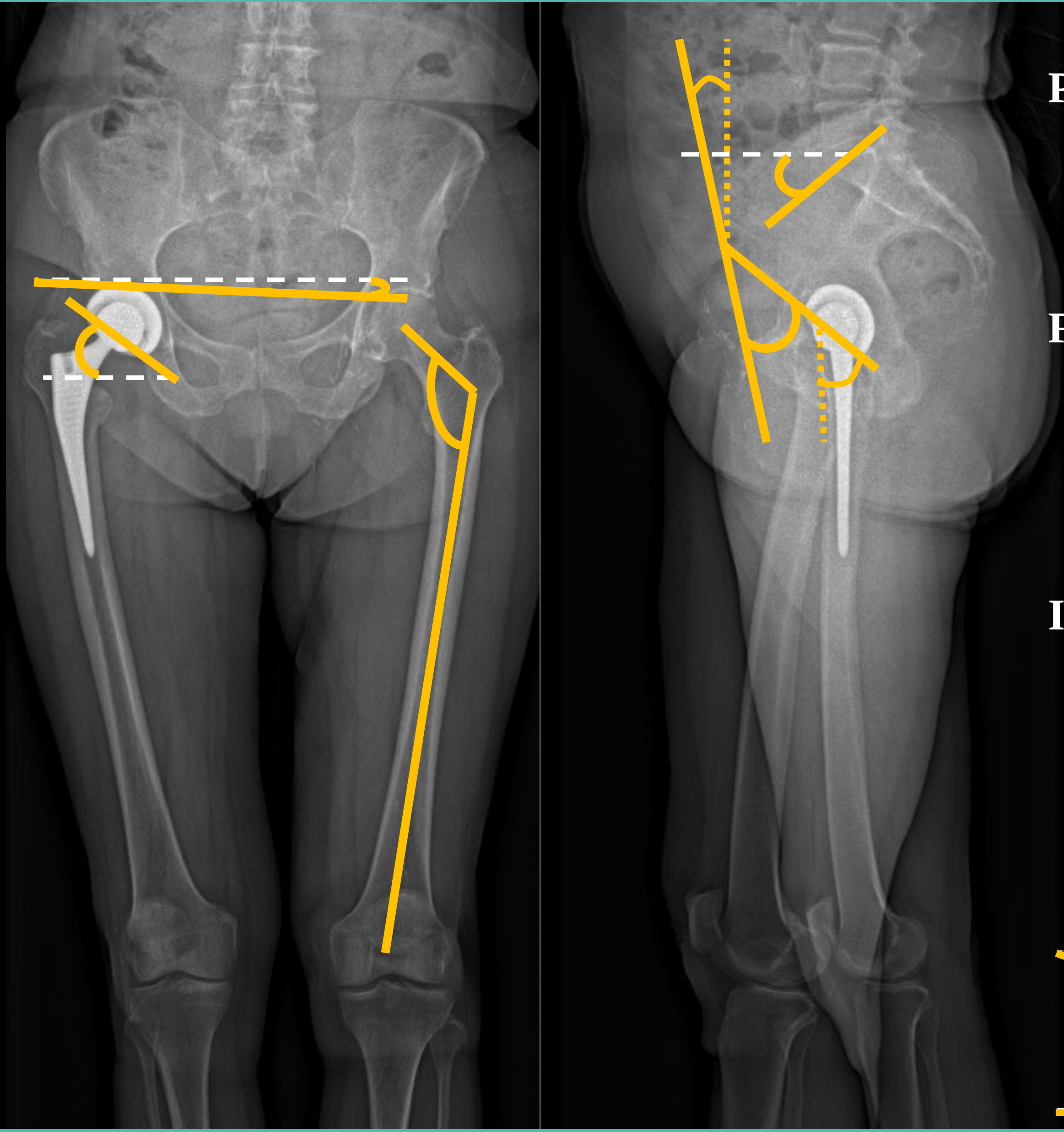

Pelvis
- Sacral Slope
- Pelvis Obliquity
- APP Inclination (*Intra*)

Femur
- Cervico Diaphyseal angle
- Femoral Torsion (*Inter*)
- Antetorsion Stem (*Intra*)

Implant
- Cup Anteversion w.r.t cabin
- Cup Anteversion w.r.t APP (Intra)
- Cup Inclination w.r.t APP

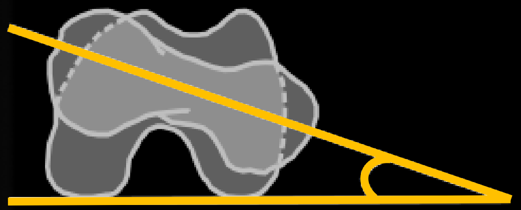

Overview good to high SDC < 5 mm – P & L

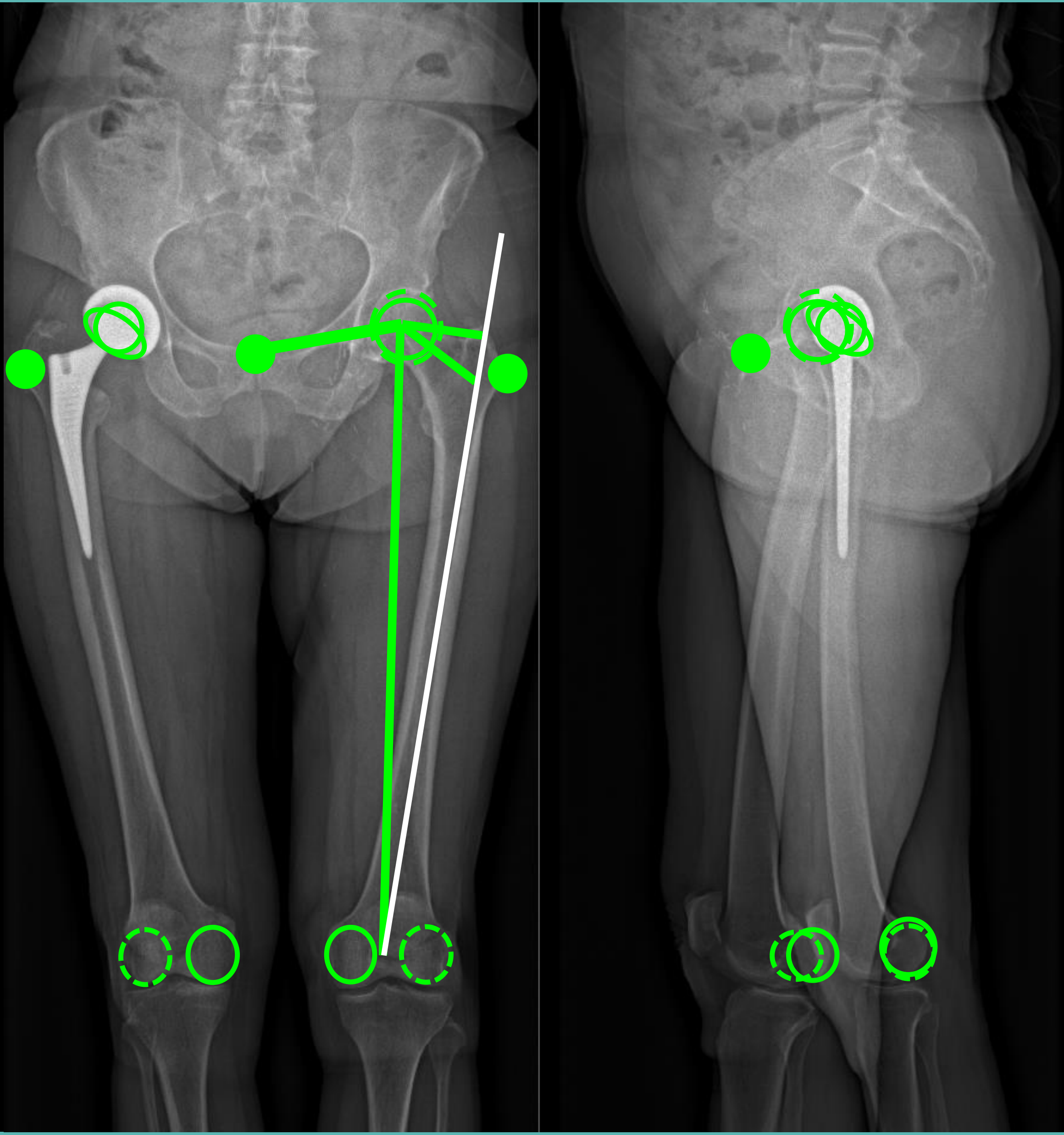

Pelvis
- Pubic Symphysis Pos.
- || PSYM – Contra. Acet. ||
- Acetabulum Pos. & Diam.

Femur
- Femoral Length
- Femoral Offset
- Femoral Neck Length
- Femoral Head Pos. & Diam.
- GT Pos. on frontal view
- Femoral Condyles Pos. & Diam.

Implant
- Stem Head & Diameter
- Cup Position & Diameter

Overview good to high SDC < 5° – Angles

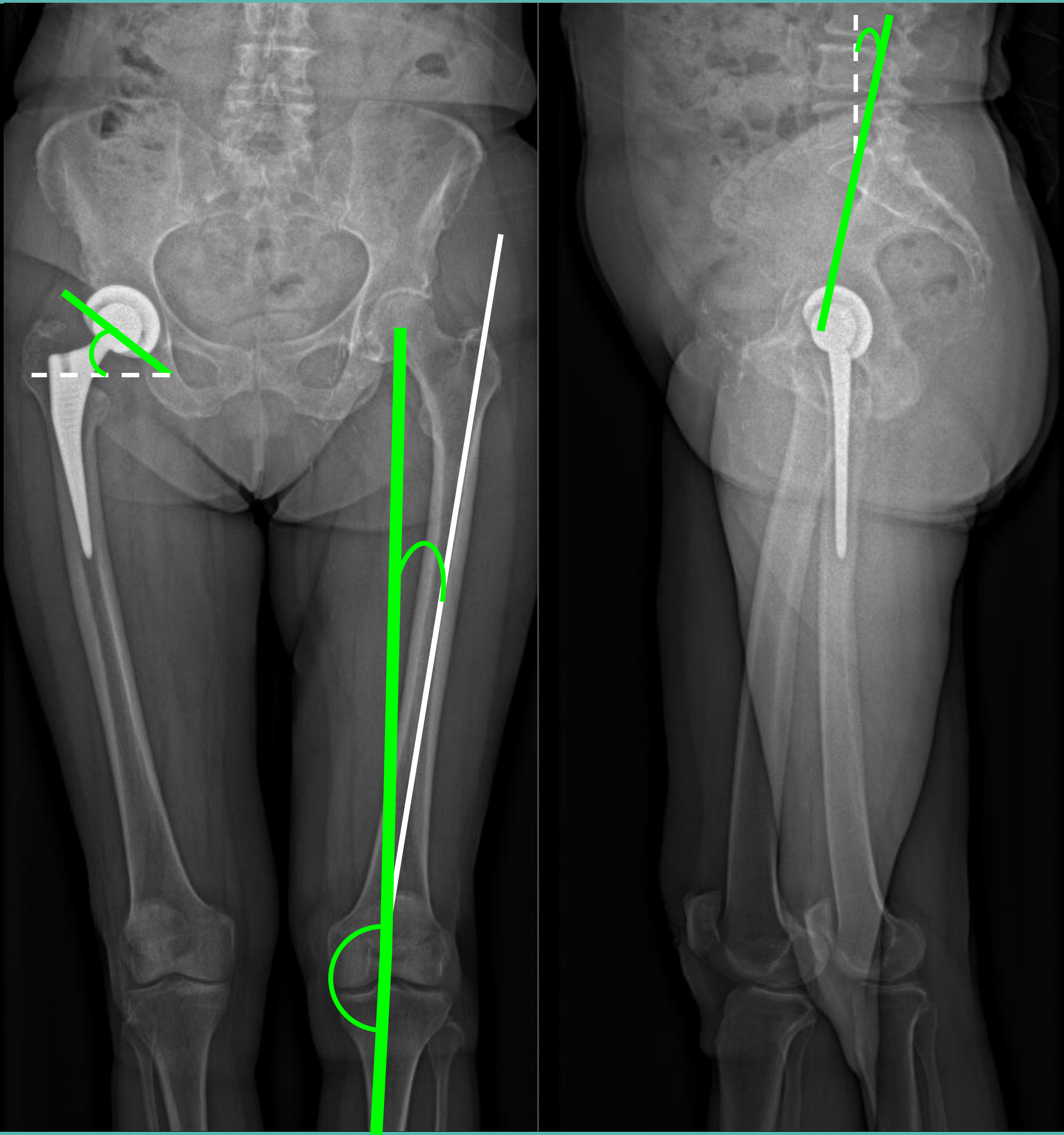

Pelvis
Pelvic Version

Femur
Mechanical Axis
Hip Knee Shaft Angle

Implant
Cup Inclination w.r.t cabin
