# Supplementary material for: Reliability of the pelvis and femur anatomical landmarks and geometry with the EOS system before and after total hip arthroplasty
Source: Sci Rep. 2022 Dec 11;12:21420. doi: 10.1038/s41598-022-25997-3 (PMC9742167; doi:10.1038/s41598-022-25997-3)
Supplement: Supplementary file 11 — Supplementary Information 11. [file 41598_2022_25997_MOESM11_ESM.pdf]

Table 1: Intra and inter-operator intra-class correlation (ICC) and smallest detectable change (SDC in mm) of the position of anatomical points for the left and right sides. *AP*: anterior-posterior direction, *ML*: medio-lateral direction, *V*: vertical direction, *R*: radius, *Ant*: anterior, *Sup*: superior, *Fem*: femoral, *Troch*: Trochanter.

		ICC				SDC (mm)			
		Intra-Operator		Inter-Operator		Intra-Operator		Inter-Operator	
**Pelvis**		Left	Right	Left	Right	Left	Right	Left	Right
Acetabulum :	AP	0.999	0.998	0.999	0.997	3.2	4.3	3.2	5.2
	ML	0.995	0.997	0.991	0.991	3.1	2.4	4.4	3.9
	V	1.000	1.000	1.000	0.999	3.0	2.3	3.0	3.5
	R	0.866	0.913	0.406	0.306	2.5	1.9	5.2	5.3
Ant. Sup. Iliac Spine	AP	0.979	0.973	0.959	0.967	15.3	17.2	21.5	19.1
	ML	0.976	0.975	0.940	0.972	6.2	8.3	9.6	8.9
	V	0.997	0.992	0.995	0.990	9.5	14.4	11.8	16.0
**Femur**		Left	Right	Left	Right	Left	Right	Left	Right
Fem. Head :	AP	0.999	0.997	0.999	0.997	3.5	5.3	5.4	3.5
	ML	0.999	0.998	0.999	0.997	1.5	1.7	2.2	1.8
	V	1.000	1.000	1.000	1.000	2.2	1.9	2.2	2.3
	R	0.933	0.913	0.908	0.893	1.6	1.7	1.9	1.9
Greater Troch. :	AP	0.997	0.997	0.998	0.995	6.0	5.2	6.0	5.2
	ML	0.994	0.990	0.992	0.990	4.4	4.5	4.5	5.0
	V	1.000	1.000	1.000	1.000	2.6	2.8	2.9	2.9
Lat. Condyle :	AP	0.999	0.999	0.999	0.999	2.8	2.9	4.5	3.8
	ML	0.993	0.991	0.985	0.990	3.2	4.3	4.4	4.5
	V	1.000	1.000	1.000	1.000	1.2	1.1	1.6	1.4
	R	0.900	0.837	0.830	0.788	1.5	2.0	2.2	1.9
Med. Condyle :	AP	1.000	0.999	0.999	0.999	2.6	3.3	3.9	3.7
	ML	0.993	0.997	0.988	0.994	3.2	2.4	3.5	4.3
	V	1.000	1.000	1.000	1.000	1.2	1.1	1.2	1.3
	R	0.895	0.890	0.777	0.788	1.6	1.7	2.4	2.3
**p-value**		**Intra**:	0.294	**Inter**:	0.098	**Intra**:	0.231	**Inter**:	0.374
**Legend**									
Excellent		Good			Moderate			Poor	

Table 2: Intra and inter-operator intra-class correlation (ICC) and smallest detectable change (SDC in mm or °) of femur geometrical parameters for the left and right sides. *Fem.:* Femoral, *Diaph*: Diaphiseal.

	**ICC**				**SDC (mm or °)**			
	**Intra-Operator**		**Inter-Operator**		**Intra-Operator**		**Inter-Operator**	
**Parameters**	Left	Right	Left	Right	Left	Right	Left	Right
Length Fem. Neck (mm)	0.950	0.942	0.937	0.932	3.1	3.3	3.7	3.5
Diameter Fem. Head (mm)	0.965	0.958	0.953	0.959	2.2	2.2	2.4	2.3
Length Fem. Neck (mm)	0.950	0.942	0.937	0.932	3.1	3.3	3.7	3.5
Fem. Offset (mm)	0.942	0.929	0.909	0.944	3.6	3.9	3.5	4.5
Hip-Knee-Shaft Angle (°)	0.910	0.883	0.798	0.873	1.3	1.3	1.5	1.7
Cervico Diaph. Angle (°)	0.837	0.884	0.850	0.834	5.5	4.7	5.6	5.3
Mechanical Angle (°)	0.802	0.845	0.756	0.783	2.2	2.1	2.3	2.7
Fem. Torsion (°)	0.802	0.800	0.658	0.741	10.8	13.3	12.4	17.4
**p-value**	**Intra:**	0.374	**Inter:**	0.051	**Intra:**	0.208	**Inter:**	0.143

**Legend**			
Excellent	Good	Moderate	Poor
